# Shedding Light on Photochemical Activation and Catalytic Mechanism of Cobalt-Catalyzed Alkene Hydroaminocarbonylation

**DOI:** 10.1021/acscatal.5c05161

**Published:** 2025-09-15

**Authors:** Sofia Lerda, Ahmet Altun, Mason S. Faculak, Erik J. Alexanian, Giovanni Bistoni

**Affiliations:** † Department of Chemistry, Biology and Biotechnology, University of Perugia, Perugia 06123, Italy; ‡ Max-Planck-Institut für Kohlenforschung, Kaiser-Wilhelm-Platz 1, Mülheim an der Ruhr D-45470, Germany; § Department of Chemistry, University of North Carolina at Chapel Hill, Chapel Hill, North Carolina 27599, United States

**Keywords:** photocatalysis, density functional theory, earth-abundant metals, carbonylation, amide synthesis

## Abstract

In a recent experimental study, a cobalt-catalyzed, light-induced synthesis of amides, achieving 100% atom economy by coupling amines and alkenes under 390 nm LED illumination, was reported. Despite its practical appeal, important mechanistic questions remain, particularly regarding the photoinduced generation of the active catalyst and the subsequent catalytic cycle. Herein, we shed light onto the mechanism of this transformation. Our results revealed how vibronic and spin–orbit coupling effects synergistically enhance the photoabsorption of the precatalyst at 390 nm, driving the formation of the active species, [Co­(CO)_3_]^−^. These insights may extend to a broader range of cobalt-catalyzed transformations where photochemical activation follows a similar pathway. Furthermore, we identify an amine-assisted nucleophilic substitution as the most favorable pathway for amide formation over a reductive elimination pathway, which involves prohibitively high activation barriers. We thus propose nucleophilic substitution as a general mechanistic motif for product formation and catalyst regeneration in other hydrocarbonylations catalyzed by [Co­(CO)_3_]^−^.

## Graphical abstract

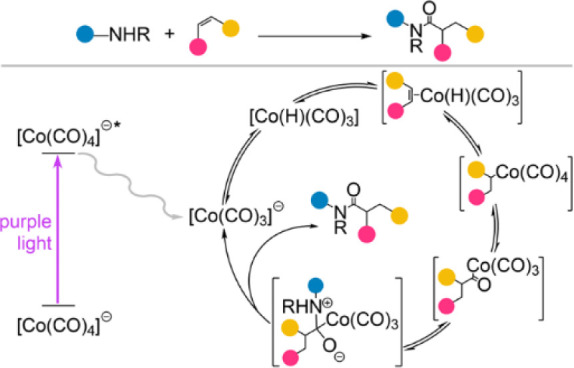

## 1 Introduction

Efficient and atom-economical methods for amide synthesis remain a significant challenge in synthetic chemistry, despite their critical importance in the pharmaceutical industry. Large-scale production often relies on stoichiometric quantities of coupling agents,^1^ leading to poor atom economy, higher costs and significant environmental impact. Recent progress has primarily centered on direct hydroaminocarbonylation catalyzed by precious metals like palladium^2–6^ and rhodium.^7^ However, these methods typically require harsh conditions, including high pressures and temperatures, while strategies employing earth-abundant metals under mild conditions remain underexplored.^8,9^

In 2024, Alexanian and coworkers reported^10^ a cobalt-catalyzed, light-induced alkene hydroaminocarbonylation that achieved 100% atom economy under mild conditions, utilizing purple light, low CO pressures (2 atm), and [Co_2_(CO)_8_] as the catalyst (Figure 1a). This method demonstrated high yields and broad substrate scope across diverse alkene and amine partners, providing a sustainable approach to amide synthesis. However, important mechanistic questions remain to fully understand the catalytic process. The proposed mechanism, presented in ref.,^10^ is shown in Figure 1b.

**Figure 1:**
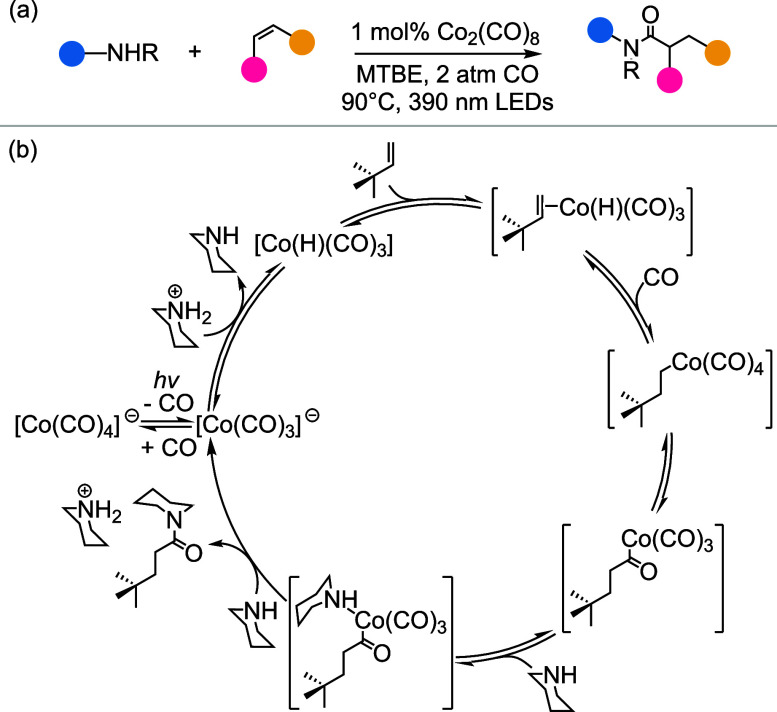
(a) Cobalt-catalyzed synthesis of amides by hydroaminocarbonylation under mild conditions promoted by light and (b) its proposed mechanism in ref. 10. Modified from Faculak et al., Cobalt-catalyzed synthesis of amides from alkenes and amines promoted by light, 10.1126/science.adk2312 [2024], AAAS. Copyright 2024, AAAS.

The involvement of 390 nm light in the reaction mechanism was hypothesized to facilitate the formation of the active species [Co­(CO)_3_]^−^ by first being absorbed by the [Co­(CO)_4_]^−^ complex present in the reaction mixture.^11^ The light-absorption was proposed to induce ligand dissociation, therefore leading to the formation of the tricarbonyl cobaltate catalyst. This hypothesis was also proposed in several previous studies,^12–15^ suggesting that the generation of [Co­(CO)_3_]^−^ from the precatalyst occurs through dissociation of one of the CO ligands. Despite the widespread proposal of [Co­(CO)_3_]^−^ formation in various cobalt-catalyzed reactions, the precise mechanism by which light facilitates its generation remains unclear. This gap in understanding hinders the rational design of new catalytic cycles that could harness this photochemical activation pathway. A deeper mechanistic insight into how light drives the formation of [Co­(CO)_3_]^−^ is therefore crucial for expanding the scope of cobalt-mediated transformations and optimizing their efficiency.

For the hydroaminocarbonylation studied here, the proposed mechanism involved light-induced formation of the active catalyst, with the formation of the amide product involving either a concerted addition and/or final reductive elimination step that also regenerates the catalyst. However, no comprehensive computational study has been conducted to validate this pathway. In this work, we provide a detailed investigation of the reaction mechanism, elucidating the role of light in catalyst activation and the subsequent steps of the catalytic cycle.

## 2 Computational Details

All the calculations were performed using the ORCA^16^ quantum chemistry program package.

For the calculation of the electronic absorption spectra of [Co­(CO)_4_]^−^, the [Co­(CO)_4_]^−^ structure was optimized using the B3LYP^17–20^ -D3­(BJ)^21,22^ /ma-def2-QZVP^23,24^ level of theory, with solvent effects (THF: tetrahydrofuran; MTBE: methyl *tert*-butyl ether)^25^ accounted implicitly via the Conductor-like Polarizable Continuum Model (CPCM).^26^ Electronic absorption spectra were then calculated at the resulting optimized geometries using the TD-B3LYP-D3­(BJ)/ma-def2-QZVP level of theory with the CPCM model, both with and without spin–orbit coupling. Oscillator strengths (f values) were all reported in the length formalism but velocity formalism provides nearly the same f values for the present molecules. The Voigt profile^27^ was used to convolve the computed line spectra into continuous spectra with Gaussian and Lorentzian fwhm values of 10 nm, accounting for both Doppler and collision broadening. This computational protocol was validated by comparing the absorption spectrum of [Co­(CO)_4_]^−^, simulated using different functionals, against experimental data^10^ and high-level CASPT2 ^28^ calculations (Figures S1 and S2). The key spectral features, including low-energy triplet-state excitations near the experimental wavelength, are well reproduced by both CASPT2 and TD-DFT. For consistency with the methods used in the mechanistic study (see below), only TD-B3LYP results are shown and discussed in the main manuscript. Additionally, the influence of the Tamm-Dancoff Approximation (TDA)^29^ was evaluated by computing the absorption spectrum with and without its application (Figure S3) and was ultimately deemed negligible.

In calculating the reaction pathways, the structures were optimized at the B3LYP-D3­(BJ)/def2-TZVP­(−f)^30^ level of theory, with solvent (MTBE) effects accounted implicitly via CPCM.^26^ The enthalpy and entropy corrections were computed at the same computational level. Electronic energies at the resulting optimized geometries were then refined at the B3LYP-D3­(BJ)/ma-def2-QZVP level with the CPCM model. A systematic comparison of activation barriers using multiple classes of exchange–correlation functionals—hybrid GGA, hybrid meta-GGA, meta-GGA, and range-separated hybrids—is reported in Tables S1 and S2. All functionals consistently identify the amine-assisted nucleophilic substitution as the most favorable pathway (see below), indicating that the main mechanistic conclusions are robust with respect to the choice of functional.

## 3 Results and Discussion

### 3.1 Precatalyst Photodissociation

Traditionally, photodissociation of a CO ligand from a metal complex has been described through two different mechanisms.^31^ For metals with partially filled d orbitals, it was attributed to a ligand field transition, in which an electron is promoted from a nonbonding metal d-orbital to an antibonding d-orbital with M-CO character. This excitation weakens the metal-CO bond, facilitating CO dissociation. More recently, however, the commonly accepted mechanism involves a metal-to-ligand charge transfer (MLCT) transition, where an electron is promoted to a π* orbital of the CO ligand. The resulting excited state relaxes toward a new equilibrium geometry, typically featuring elongated metal–ligand bonds and a reduced dissociation barrier.

In the original experimental study,^10^ [Co­(CO)_3_]^−^ was proposed as the active catalyst of the transformation, presumed to originate from [Co­(CO)_4_]^−^ via photoinduced ligand dissociation in the presence of 390 nm light. This species is likely to form in the reaction conditions from the [Co_2_(CO)_8_] precursor.^11,12^ To elucidate whether the formation of [Co­(CO)_3_]^−^ from [Co­(CO)_4_]^−^ and light at 390 nm is possible under the reaction conditions, we initially carried out an in-depth analysis of the geometric and electronic structure of [Co­(CO)_4_]^−^.

The singlet tetrahedral configuration is 49.23 (49.14) kcal/mol more stable than the triplet structure (Figure 2a) in THF (MTBE). The electronic structure of the singlet ground state was initially analyzed by examining its molecular orbitals (Figure 2c). The local electronic configuration at the metal can be described as Co 3d^10^. As expected for the tetrahedral [Co­(CO)_4_]^−^, the d orbitals exhibited typical crystal field splitting (Figure 2b). Five nearly degenerate LUMOs were found, each of them exhibiting antibonding character between the metal center and the ligands. This finding is in agreement with the hypothesis that the light-induced promotion of a d electron to an antibonding LUMO would weaken the Co–C bond, thereby favoring the dissociation of a CO ligand and the formation of [Co­(CO)_3_]^−^.

**Figure 2:**
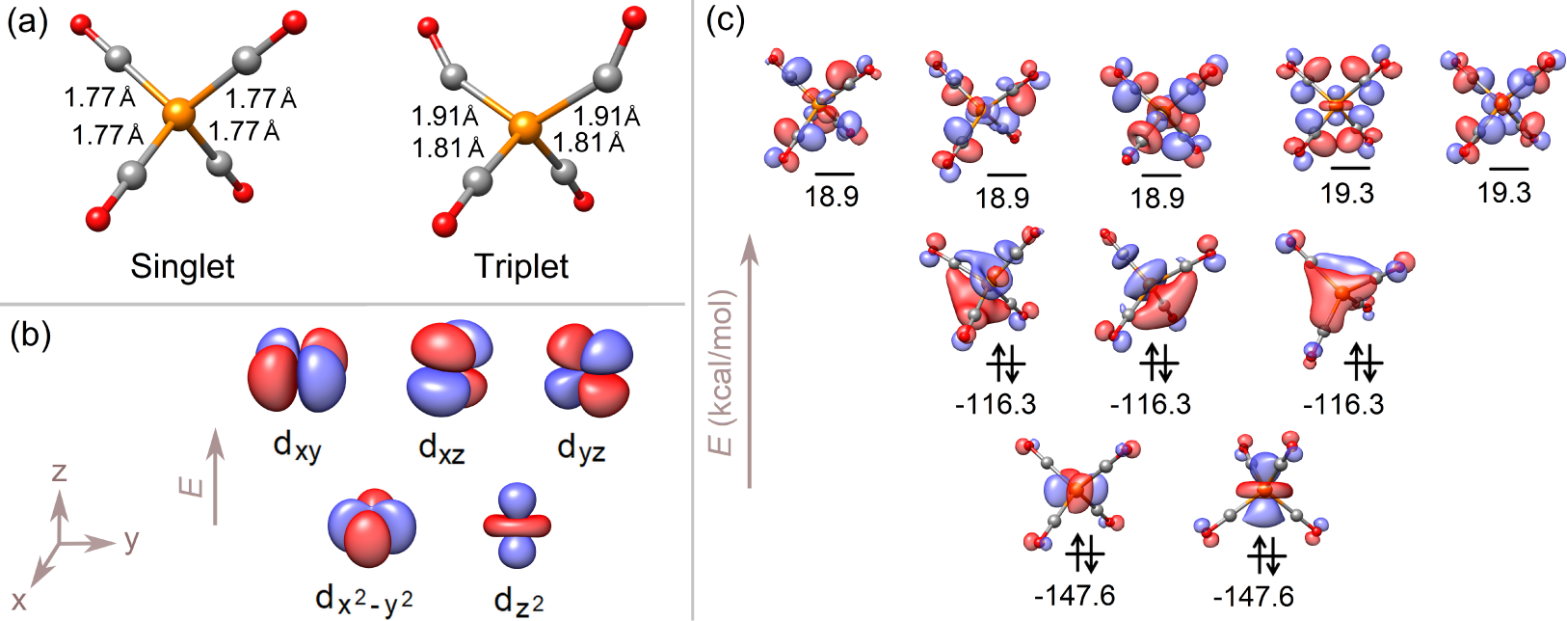
(a) Computed structure of [Co­(CO)_4_]^−^ in the singlet ground and excited triplet states - identical in THF and MTBE (within two decimal digits). (b) Crystal-field splitting of d-orbitals under *T*_d_ symmetry. (c) Frontier molecular orbitals of singlet [Co­(CO)_4_]^−^, presenting typical crystal field splitting of d orbitals: two MOs (e terms), corresponding to the $d_{x^{2}-y^{2}}$ and $d_{z^{2}}$ orbitals of cobalt, were found at lower energy, while three orbitals (2 t_2_ terms), associated with the d_*xz*_, d_*yz*_, and d_*xy*_ orbitals of the metal, appear at higher energy. Reported orbital energies (kcal/mol) are obtained in THF. In MTBE, the qualitative picture remains the same.

The spectroscopic properties of the tetracarbonyl cobaltate were analyzed by means of TD-DFT. Since the unoccupied frontier orbitals show stronger metal–ligand mixing than the occupied frontier orbitals (Figure 2c), all excitations between 260 and 500 nm involve noticeable MLCT character (Figure S5). This observation is consistent with the second dissociation pathway described above, where an MLCT transition promotes an electron from a metal-centered d-orbital to an antibonding orbital associated with the CO ligand. This excitation then leads the system to relax into a distorted geometry characterized by elongated M–CO bonds (see for example the triplet state equilibrium geometry in Figure 2a), which are more prone to dissociation.

Energy-wise, the strongest absorption in the experimental spectrum is the 280 nm region. Our TD-DFT results revealed that several excitations contribute to this band. These excitations could potentially lead to promotion to an excited state in which the dissociation of a ligand to form the tricarbonyl complex is possible; existing literature on related systems such as [Fe­(CO)_5_],^32^ [Co­(CO)_3_NO],^33,34^ and the isoelectronic [Ni­(CO)_4_][Bibr ref35] suggests that the energy associated with these transitions could be large enough to lead to successive ligand dissociations.

Conversely, lower-energy transitions within the experimental range (∼390 nm) could potentially facilitate the dissociation of a single ligand, leading to the formation of the [Co­(CO)_3_]^−^ species of interest.^33,36^ While the experimental spectrum exhibits very weak absorption at 390 nm, in the computed spectrum no excitations appear in this region when spin–orbit coupling (SOC) effects are neglected (see the red line in the inset of Figure 3a, which is along the baseline, indicating negligible absorption in this region).

**Figure 3:**
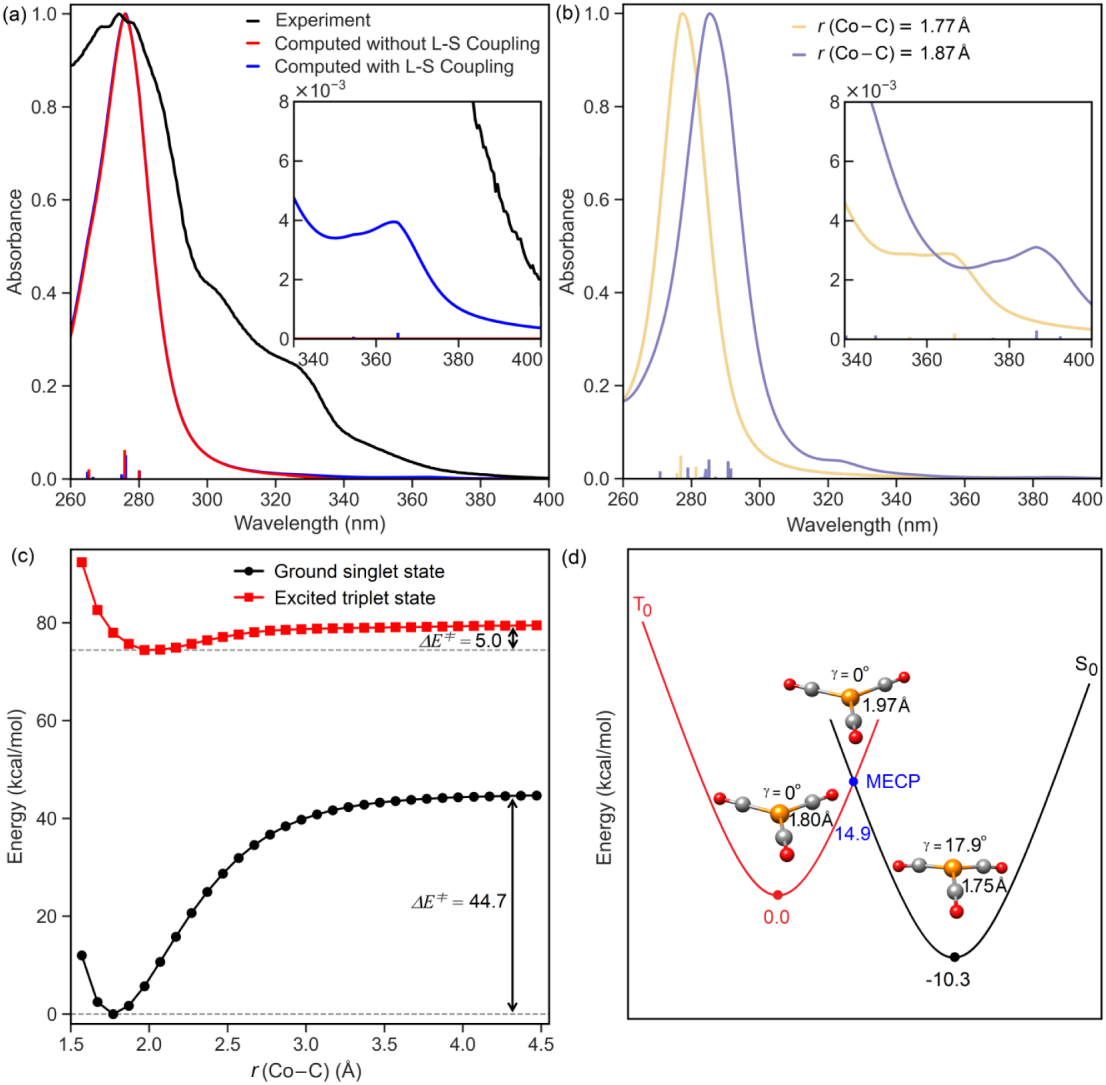
(a) TD-DFT absorption spectra of [Co­(CO)_4_]^−^ with and without spin–orbit (L-S) coupling in THF, compared to the experimental UV–vis spectrum of K­[Co­(CO)_4_] in THF. The inset highlights the weak 340–400 nm absorption, visible only with L-S coupling. (b) TD-DFT spectra of [Co­(CO)_4_]^−^ with L-S coupling in MTBE at equilibrium and with one Co–C bond elongated by 0.1 Å. (c) Relaxed PES scan of ground singlet and triplet states of [Co­(CO)_4_]^−^ in MTBE along a Co–C bond stretch. (d) Structures and relative energies of ground singlet and triplet states of [Co­(CO)_3_]^−^ in MTBE, including their minimum energy crossing point, Co–C distances, and Co out-of-plane angles.

Remarkably enough, when SOC effects are included, however, certain triplet excitations become allowed, manifesting as a weak band in the spectrum (Figure 3a).^37^ It is worth noting that this excitation shifts to longer wavelengths and increases in intensity when the Co–C bond is slightly elongated, suggesting that thermal vibrational effects naturally occurring in solution under the experimental conditions could enhance absorption into a triplet state at the experimental wavelength (Figure 3b and Supporting Information).

As shown in Figure 3c, ligand dissociation in the ground-state [Co­(CO)_4_]^−^ is not feasible under the experimental conditions due to the prohibitively high barrier (44.7 kcal/mol), effectively ruling out thermal dissociation in the singlet state. In contrast, in the triplet state, the ligand dissociation barrier (5.0 kcal/mol) is significantly lower and can be easily overcome. Importantly, the energy gap between the triplet and singlet potential energy surfaces decreases with Co–C bond elongation, from ∼ 80 kcal/mol at the singlet equilibrium geometry to ∼ 40 kcal/mol near the dissociation limit. This trend aligns with the earlier observation that Co–C elongation facilitates the key excitation promoting ligand dissociation at lower energies.

Once triplet [Co­(CO)_3_]^−^ is formed, the minimum energy crossing point (MECP) at 14.9 kcal/mol enables efficient intersystem crossing between the triplet and singlet potential energy surfaces (Figure 3d), ultimately yielding the active catalyst, singlet [Co­(CO)_3_]^−^. Similar cases have been reported,^37^ in which the intermediate formed after CO dissociation was assigned a triplet spin state, while both the reactants and final products were in singlet states.

In summary, our calculations ruled out the possibility of thermal dissociation of a single CO ligand in the ground state singlet. In contrast, under the experimental conditions, it was found that 390 nm light can indeed induce the dissociation of a single CO ligand. Specifically, SOC and vibronic effects enable an otherwise forbidden transition to a triplet state, where the ligand dissociation barrier is low. Subsequent intersystem crossing yields the active catalyst in the singlet state. These findings are consistent with the reported high quantum yield,^10^ suggesting multiple turnovers per photon and supporting the hypothesis that light promotes catalyst activation rather than directly participating in the catalytic cycle.

### 3.2 Mechanistic Study

Due to their high experimental yields,^10^ piperidine and 3,3-dimethyl-1-butene were chosen as the amine and alkene partners, respectively. The reaction mechanism first begins with the protonation of tricarbonyl cobaltate. The newly formed hydride coordinates the alkene partner, and the resulting species then undergoes hydrocobaltation, leading preferentially to the formation of the linear isomer. The insertion of a CO ligand between the metal center and the carbon chain then takes place, leading to acylcobalt. At this point, two mechanistic possibilities to form the amide product can be envisioned involving either a reductive elimination or nucleophilic acyl substitution pathway.^10^

For all intermediates, calculations on the singlet, triplet and quintet spin states (Figure S8) showed no crossing between potential energy surfaces. Hence, our study focused on the lowest energy singlet state exclusively. As depicted in Figure 4, a detailed exploration of the singlet potential energy surface revealed the reaction to be thermodynamically favored.

**Figure 4:**
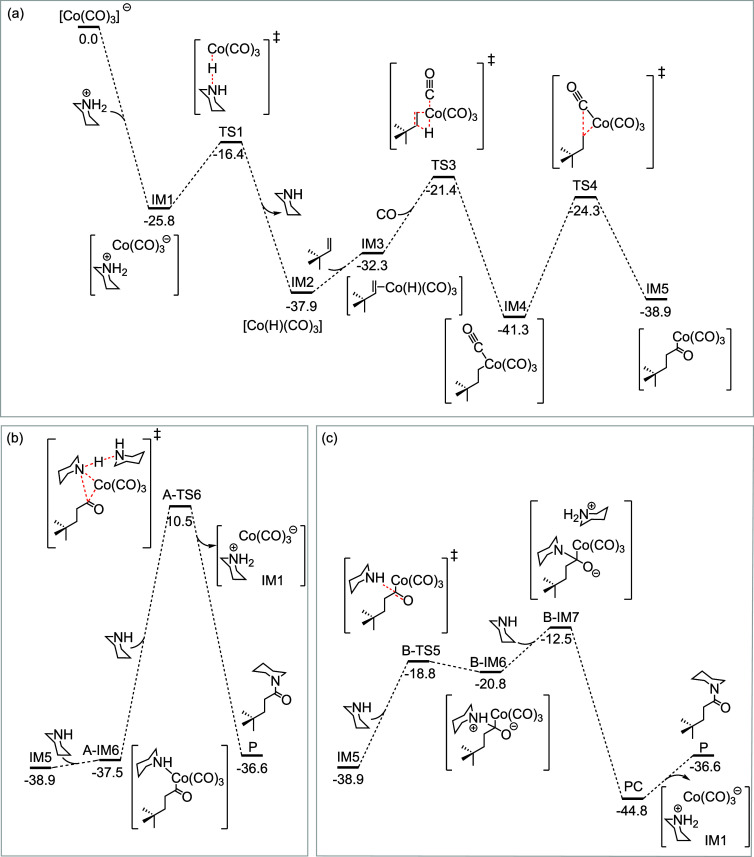
Detailed free energy pathway (a) up to acylcobalt IM5 formation (b) from IM5 to the amide product via reductive elimination. (c) from IM5 to the amide product via amine-assisted nucleophilic substitution. All energies are given in kcal/mol relative to the first point on the potential energy surface.

The steps leading from IM2 to IM3 and from IM5 to A-IM6 were found to be barrierless, as they consist in simple coordination events (note that this result is based on an implicit solvent model and does not account for entropy or explicit solvent effects, which could introduce small barriers in reality). Once the product P is formed, the cycle restarts from IM1. While the kinetic barriers associated with most steps were found to be reasonable, the formation of the final product via reductive elimination causes the overall energy barrier to rise to the prohibitively high value of 51.8 kcal/mol (Figure 4b). Alternative pathways involving nucleophilic substitution instead of reductive elimination were therefore considered.

Among the alternative routes explored (Figures S10 and S11), an amine-assisted nucleophilic substitution emerged as the most favorable mechanism (Figure 4c). The final catalytic cycle supported by our calculations is depicted in Figure 5.

**Figure 5:**
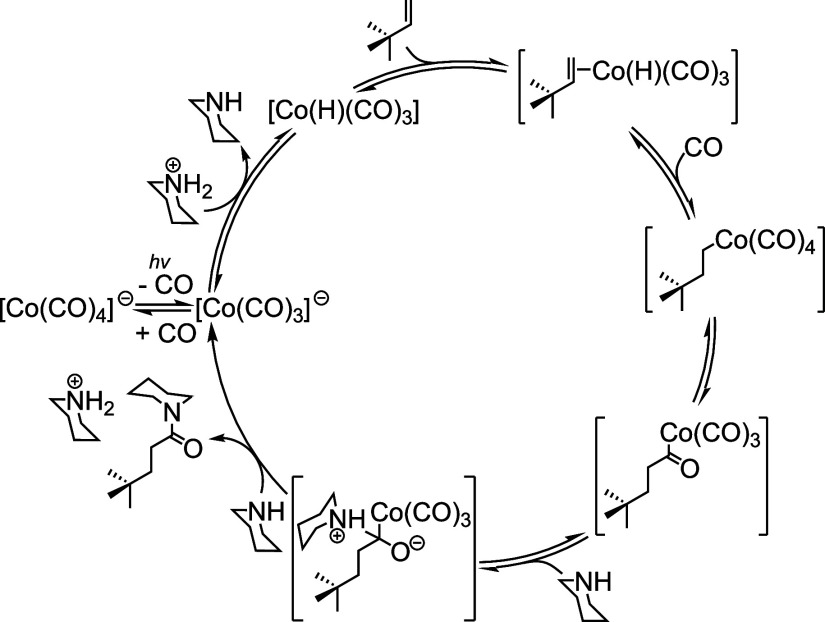
Nucleophilic substitution facilitated by the amine cocatalyst, as supported by our computational results.

In this pathway, an additional amine facilitates the process and lowers the energy barrier by accepting a hydrogen atom from the nitrogen in intermediate B-IM6, thereby prompting the release of the catalyst. Notably, both the proton transfer from B-IM6 to the amine and the subsequent release of the cobalt species were found to proceed without any energy barrier. Hence, the overall energy barrier for this route, 28.8 kcal/mol, which was computed as the difference between the energy of B-IM7 and IM-4, was found to be consistent with the experimental reaction time frame.

Very recently, this catalytic platform was also successfully applied to alkene alkoxy- and hydroxycarbonylations.^38^ The acyl nucleophilic substitution pathway is also preferred in these reactions (Figure 6).^38^

**Figure 6:**
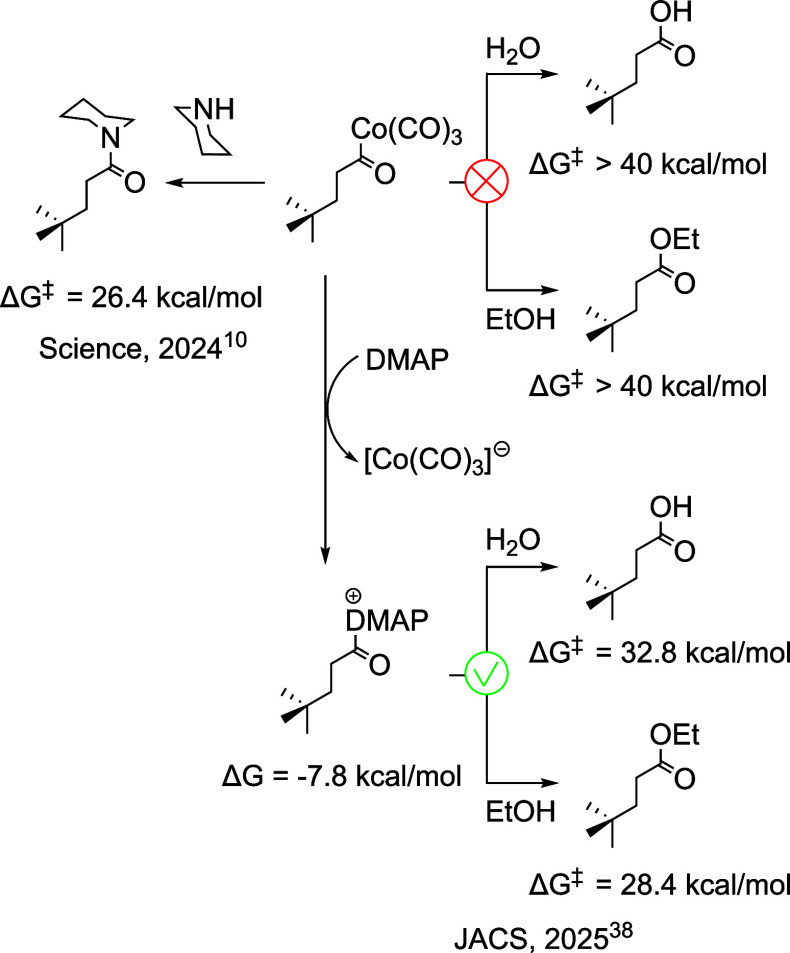
Schematic representation of the potential products accessible via the cobalt-catalyzed mechanism by varying the nucleophile. The figure also illustrates that, in the absence of the DMAP cocatalyst, the reaction is kinetically unfeasible.

In the reported alkene alkoxycarbonylation and hydroxycarbonylation reactions, 4-dimethylaminopyridine (DMAP) was included as a cocatalyst. The authors proposed that DMAP facilitates the acyl transfer step from intermediate IM5. Our calculations support the intermediacy of an acylpyridinium formed from IM5 and DMAP and subsequent nucleophilic attack by either H_2_O or EtOH to deliver the corresponding carboxylic acid or ester. In the absence of DMAP, the overall reaction would not be kinetically favorable.

## 4 Conclusions

This study sheds light into the mechanism that drives cobalt-catalyzed, light-induced hydroaminocarbonylation reactions. We have elucidated the role of spin–orbit and vibronic coupling effects in enabling the activation of the precatalyst under 390 nm illumination, thereby providing a clear mechanistic basis for the generation of the active [Co­(CO)_3_]^−^ species. Our findings highlight how forbidden electronic transitions can be enhanced by vibrational motion and spin–orbit interactions, ultimately facilitating ligand dissociation under experimental conditions.

Our exploration of the catalytic cycle reveals that, among the pathways previously proposed for closely related cobalt chemistries—namely, reductive elimination and nucleophilic substitution—the latter is clearly favored. In particular, an amine-assisted nucleophilic substitution pathway emerges as the most viable route for amide formation from the acyl-cobalt intermediate. Our computational analysis shows that the energy barrier for reductive elimination is significantly higher than that for nucleophilic substitution, making the latter the preferred mechanism under the reaction conditions.

Beyond the specific case of cobalt-catalyzed amide synthesis, the mechanistic principles elucidated in this work—particularly the role of spin–orbit and vibronic coupling in promoting photodissociation, and the identification of nucleophilic substitution as a key step in product formation—offer broader insight into the reactivity of photogenerated [Co­(CO)_3_]^−^ complexes. These findings suggest that similar mechanistic pathways—featuring photoinduced ligand dissociation and nucleophile-assisted substitution—may be operative in a broad range of previously reported cobalt-mediated, light-driven transformations. As such, this study offers a general framework for understanding and rationally expanding the scope of photochemical transition metal catalysis.

## Supplementary Material



## Notes

Cartesian coordinates of the structures as well as additional data for the absorption spectra and reaction mechanisms (PDF)
