# Supplementary material for: Shedding Light on Photochemical Activation and Catalytic Mechanism of Cobalt-Catalyzed Alkene Hydroaminocarbonylation
Source: ACS Catal. 2025 Sep 15;15(19):16506–12. doi: 10.1021/acscatal.5c05161 (PMC12502662; doi:10.1021/acscatal.5c05161)
Supplement: Supplementary file 1 [file cs5c05161_si_001.pdf]

Supporting Information for

Shedding Light on Photochemical Activation and Catalytic Mechanism of Cobalt-Catalyzed Alkene Hydroaminocarbonylation

Sofia Lerda,[a] Ahmet Altun,[b] Mason S. Faculak,[c] Erik J. Alexanian,[c] Giovanni Bistoni[a,*]

[a] *Department of Chemistry, Biology and Biotechnology, University of Perugia, 06123 Perugia, Italy*

[b] *Max-Planck-Institut für Kohlenforschung, Kaiser-Wilhelm-Platz 1, D-45470 Mülheim an der Ruhr, Germany*

[c] *Department of Chemistry, University of North Carolina at Chapel Hill, Chapel Hill, NC 27599, USA.*

[*] Email: giovanni.bistoni@unipg.it

Contents

1. Electronic Absorption Spectrum of $[Co(CO)_4]^{-}$ 3

2. Reaction mechanisms 7

2.1. The Effect of Basis Set on the Detailed Singlet Reductive Elimination and Amine-Assisted Nucleophilic Substitution Pathways 7

2.2. Computational Protocol Validation 8

2.3. Reductive Elimination and Amine-Assisted Nucleophilic Substitution on Singlet, Triplet, and Quintet Surfaces 9

2.4. An Alternative Nucleophilic Substitution Mechanism 10

2.5. Nucleophilic Substitution with Different Substrates 13

2.6. Alternative Pathways to Catalyst Formation 14

2.6.1. Proton Transfer to $[Co(CO)_4]^{-}$ 14

2.6.2. Solvent Coordination to Metal Center 16

2.7. Optimized Cartesian Coordinates in Å 17

2.7.1. Equilibrium Geometries $[Co(CO_4)]^{-}$ in THF 17

2.7.2. Equilibrium Geometries $[Co(CO_4)]^{-}$ in MTBE 17

2.7.3. Singlet Potential Energy Surface of $[Co(CO_4)]^{-}$ in MTBE Along a Co–C Bond 17

2.7.4. Geometries $[Co(CO_3)]^{-}$ in MTBE 22

2.7.5. Reaction Mechanisms 22

2.7.6. Benchmark: Geometry of Key Intermediates 36

2.7.7. Proton Transfer to $[Co(CO)_4]^{-}$ 45

2.7.8. Solvent Coordination to Metal Center 46

References 47

1. Electronic Absorption Spectrum of $[Co(CO)_4]^-$

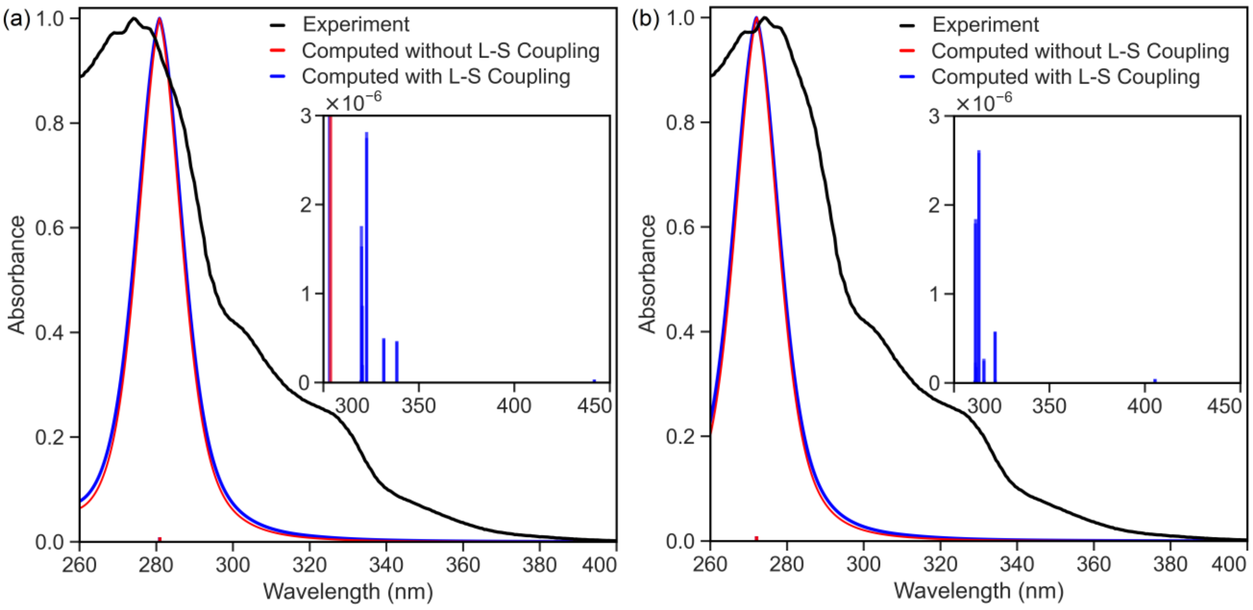

Figure S1. State-averaged fully internally contracted CASPT2(10,10)/def2-TZVP electronic absorption spectra of $[Co(CO)_4]^-$ in THF solvent (treated with CPCM), computed with and without spin-orbit (L-S) coupling. (a) A small imaginary shift of 0.03 E_h was applied to mitigate intruder states. (b) To assess the sensitivity of the results to this parameter, a larger shift of 0.1 E_h was also applied. The empirical IPEA shift was not applied. The calculations were performed at the B3LYP-D3(BJ)/ma-def2-QZVP optimized geometry and are based on a CASSCF(10,10) reference including 8 singlet and 6 triplet roots. The computed spectra are compared to the experimental UV-Vis spectrum of $K[Co(CO)_4]$ in THF.

In this study, CASPT2 was employed as the high-level method due to its proven efficacy in accurately describing the electronic structure of first-row transition-metal (TM) carbonyl complexes.[1,2] No IPEA shift was applied, as its general use is not supported by recent studies.[2,3] To mitigate potential intruder states, a small imaginary level shift of 0.03 E_h was applied (Figure S1a). A sensitivity analysis, employing a larger shift of 0.1 E_h (Figure S1b), revealed only minor variations in the computed spectra, affirming the robustness of our results. Although NEVPT2 inherently avoids intruder-state problems, it was not used here due to its limitations in describing the excited states of first-row TM complexes.[4]

Importantly, all key spectral features - most notably, the presence of triplet-state excitations near the experimental excitation wavelength - are consistently reproduced by both CASPT2 and TD-B3LYP calculations. This agreement supports the reliability of TD-B3LYP for describing the photophysical properties of the system. For consistency with the computational setup employed in the mechanistic investigation, only TD-B3LYP results are discussed in the main manuscript.

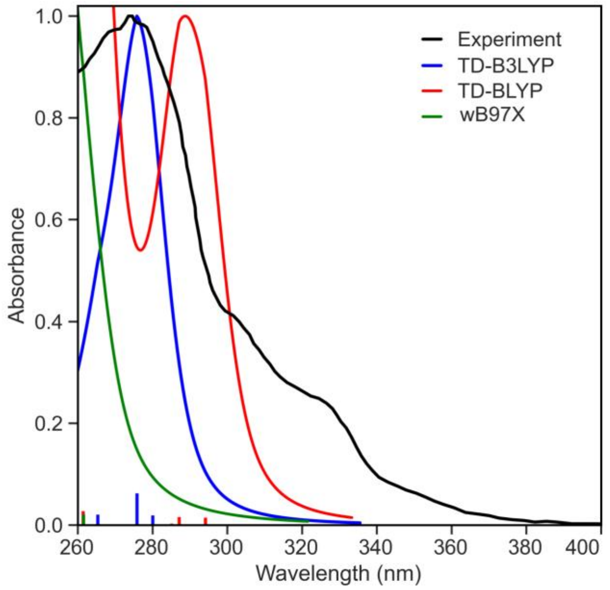

Figure S2. Computed TD-DFT/ma-def2-QZVP (DFT = B3LYP, BLYP[5,6], and ωB97X[7]) electronic absorption spectra of $[Co(CO)4]^{-}$ without spin-orbit (L-S) coupling in THF solvent (treated with CPCM) at the corresponding B3LYP-D3(BJ), BLYP2,3-D3(BJ), and ωB97X geometries in comparison with the experimental UV-Vis spectrum of K[Co(CO)4] in THF.

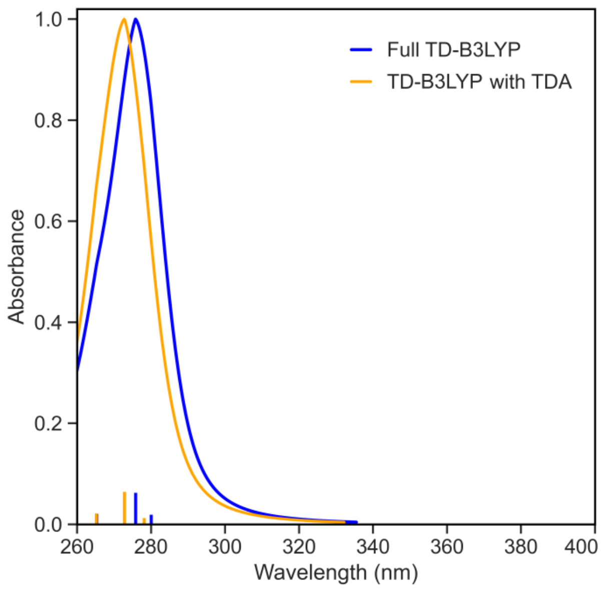

Figure S3. Computed TD-B3LYP/ma-def2-QZVP electronic absorption spectra of $[Co(CO)4]^{-}$ without spin-orbit (L-S) coupling in THF solvent (treated with CPCM) at the B3LYP-D3(BJ)/ma-def2-QZVP geometry with and without the Tamm-Dancoff Approximation (TDA).

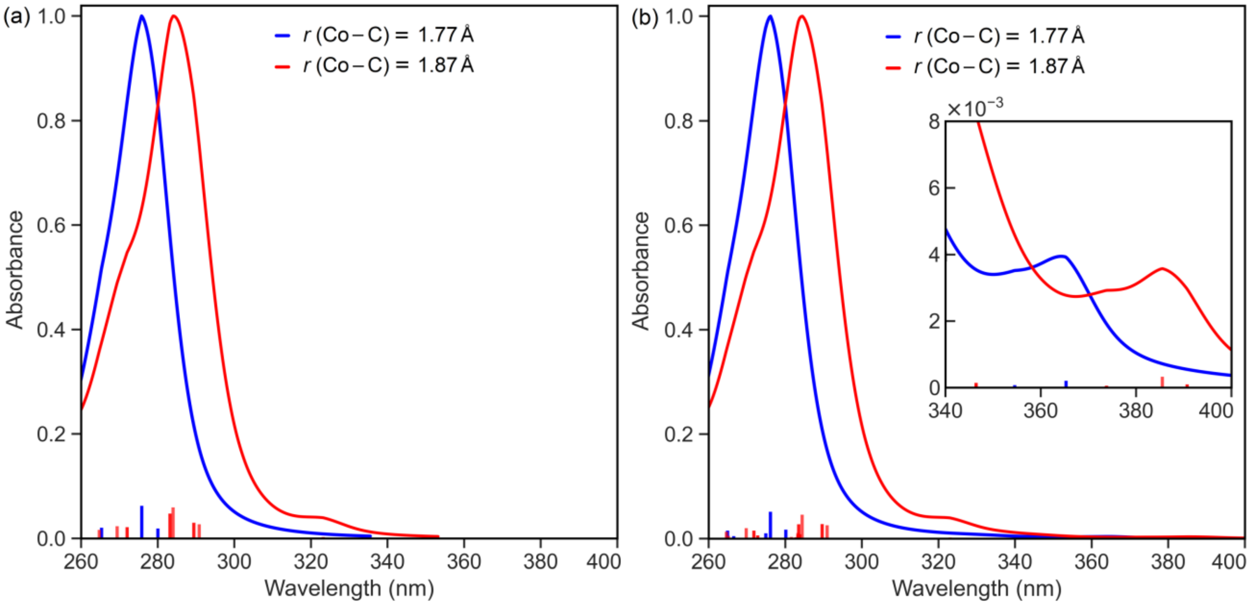

Figure S4. Computed electronic absorption spectra of [Co(CO)4]⁻ at the TD-B3LYP/ma-def2-QZVP level, incorporating THF solvent effect via CPCM (a) without L-S coupling and (b) with L-S coupling. The spectra were computed at the optimized B3LYP-D3(BJ)/ma-def2-QZVP equilibrium geometry [r(Co–C) = 1.77 Å] and at the geometry where one Co–C bond is elongated by 0.1 Å [r(Co–C) = 1.87 Å]. The inset highlights the weak absorption range from 340 to 400 nm in the presence of L-S coupling.

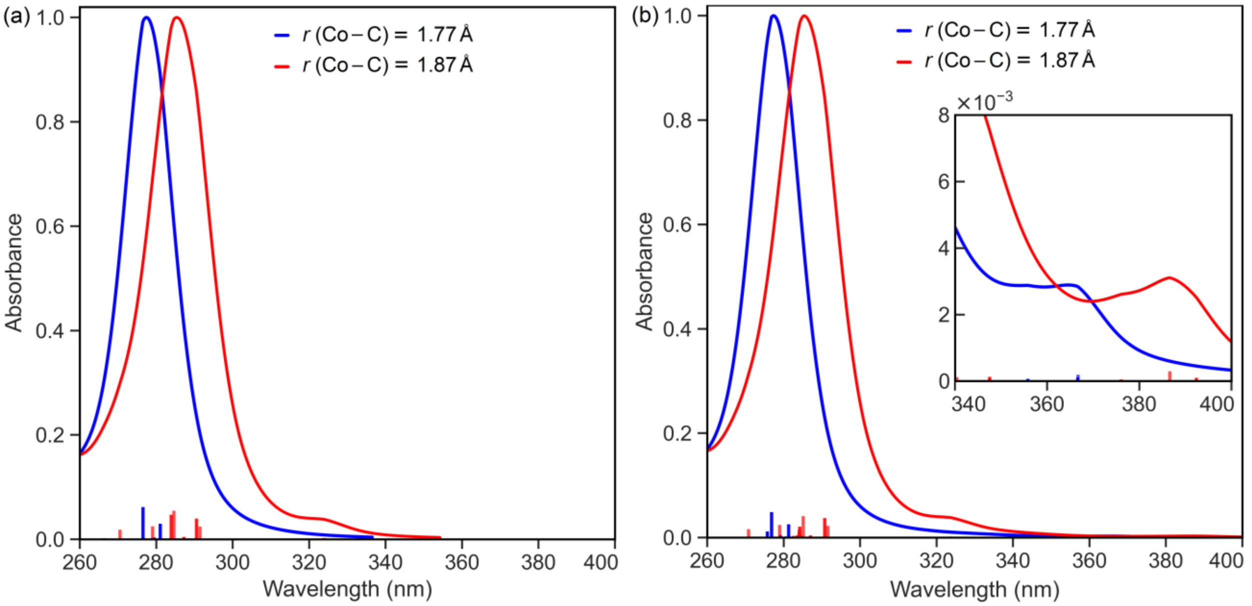

Figure S5. Computed electronic absorption spectra of [Co(CO)4]⁻ at the TD-B3LYP/ma-def2-QZVP level, incorporating MTBE solvent effect via CPCM (a) without L-S coupling and (b) with L-S coupling. The spectra were computed at the optimized B3LYP-D3(BJ)/ma-def2-QZVP equilibrium geometry [r(Co–C) = 1.77 Å] and at the geometry where one Co–C bond is elongated by 0.1 Å [r(Co–C) = 1.87 Å]. The inset highlights the weak absorption range from 340 to 400 nm in the presence of L-S coupling.

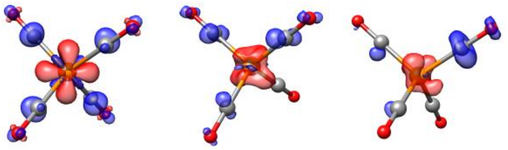

Figure S6. Difference electron densities of one of the most intense transitions around each band maximum of the computed electronic absorption spectrum of $[Co(CO)4]^{-}$ at the equilibrium geometry with L-S coupling at the TD-B3LYP/ma-def2-QZVP level, incorporating THF solvent effect via CPCM. Red corresponds to the regions where electron density decreases from the ground-state to the excited state, while blue corresponds to an increase in the electron density.

2. Reaction mechanisms

2.1. The Effect of Basis Set on the Detailed Singlet Reductive Elimination and Amine-Assisted Nucleophilic Substitution Pathways

Figure 4 of the main paper includes the results with ma-def2-QZVP. Figure S7 also includes the results with smaller def2-TZVP(-f) basis set. As seen in Figure S7, the results with smaller def2-TZVP(-f) (in parenthesis) and ma-def2-QZVP (out of parenthesis) are analogous, except A-TS6 that appears even higher in energy with the refined results using the large basis set. Therefore, in the following, unless stated otherwise, the results of preliminary calculations belong to those obtained with def2-TZVP(-f).

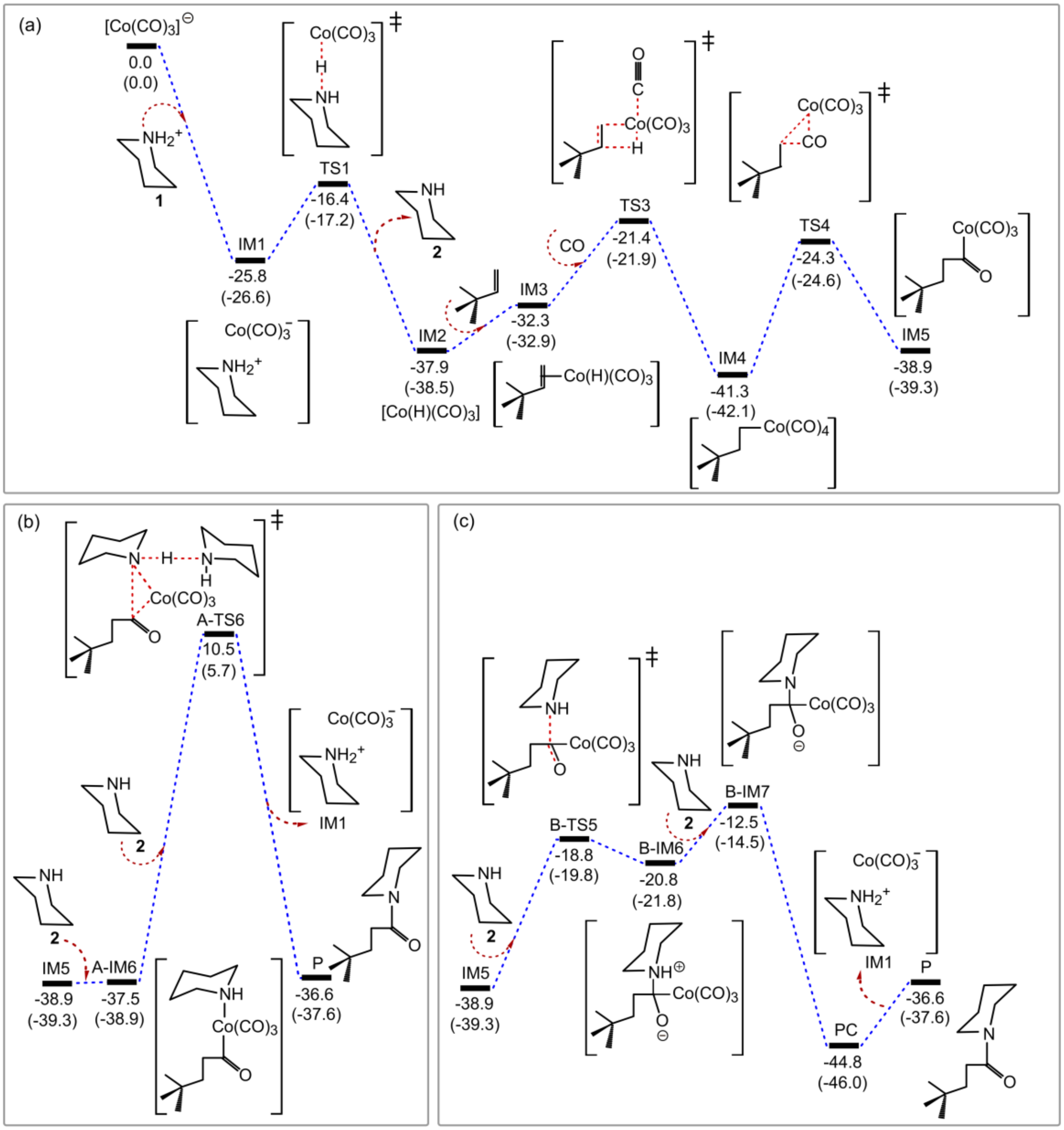

Figure S7. Detailed free energy pathway on the ground singlet surface at the B3LYP-D3(BJ)/ma-def2-QZVP (out of parenthesis) and B3LYP-D3(BJ)/def2-TZVP(-f) (in parenthesis) levels (a) up to IM5 formation (b) from IM5 through reductive elimination. (c) from IM5 through the amine-assisted nucleophilic substitution. All energies are given relative to the first point on the potential energy surface.

2.2. Computational Protocol Validation

In order to test the suitability of the B3LYP density functional to properly reproduce structures and energies of the molecules under study, we performed a series of both geometry optimizations and single point calculations using a variety of functionals paired with D3BJ dispersion corrections. Our results indicate that while different functionals may lead to variations in quantitative values, the qualitative and conceptual picture remains the same. The data in Table S1 were obtained from single-point energy calculations using the chosen method in combination with the ma-def2-QZVP basis set, performed on geometries optimized at the B3LYP-D3BJ/def2-TZVP(-f) level. When required, thermodynamic corrections were computed at the same level. Solvent effects (MTBE) were included implicitly using the C-PCM approach. Grimme's D3BJ dispersion correction was applied to all functionals except ωB97X-D3BJ, for which dispersion is already included through parametrization.

	B3LYP D3BJ	PBE0 D3BJ	TPSSh D3BJ	wB97X-D3BJ	R2SCAN D3BJ	r2SCANh D3BJ
Reductive Elimination Barrier $\Delta G^{\ddagger}$	51.83	51.59	52.13	61.91	48.05	50.90
Amine-facilitated SN Barrier $\Delta G^{\ddagger}$	28.85	23.57	25.85	32.12	21.57	23.14
$[Co(CO)_4]^-$ (S_0) CO dissociation ΔE	44.70	49.97	51.62	47.70	55.31	53.86
Vertical Excitation on Equilibrium $[Co(CO)_4]^-$ $\Delta E^{\ddagger}$	77.97	75.74	81.11	83.58	82.68	78.42

Table S1. Energy differences computed with different functionals at ma-def2-QZVP-CPCM(MTBE) on fixed B3LYP-D3BJ/def2-TZVP(-f) geometries. Values are reported in kcal/mol.

In addition to evaluating reaction energetics, the reliability of the B3LYP functional in reproducing molecular geometries was assessed by optimizing the intermediates corresponding to the lowest and highest energy points along the pathway. Geometry optimizations and thermodynamic corrections were performed at the def2-TZVP(-f) level, while electronic energies were refined using the ma-def2-QZVP basis set. Although the absolute values of the energy barriers vary across different computational methods, our results (Table S2) consistently support the main conclusion: the amine-assisted SN pathway presents a significantly lower barrier than the reductive elimination route, identifying it as the most favorable mechanism for product formation.

	B3LYP D3BJ	PBE0 D3BJ	TPSSh D3BJ	wB97X-D3BJ	R2SCAN D3BJ	r2SCANh D3BJ
Reductive Elimination Barrier $\Delta G^{\ddagger}$	51.83	43.81	43.67	54.37	40.37	43.23
Amine-facilitated SN Barrier $\Delta G^{\ddagger}$	28.85	16.12	18.44	11.17	22.86	15.58

Table S2. Energy differences computed with different functionals at ma-def2-QZVP-CPCM(MTBE) on reoptimized geometries using the corresponding functional and the def2-TZVP(-f) basis set. Solvent (MTBE) effects are included through the CPCM methods. Values are reported in kcal/mol.

2.3. Reductive Elimination and Amine-Assisted Nucleophilic Substitution on Singlet, Triplet, and Quintet Surfaces

As seen in Figure S8, the energies of intermediates on the catalytic cycle indicate no crossing among singlet, triplet, and quintet potential energies surfaces, and thus only the most stable singlet surface was explored in detail.

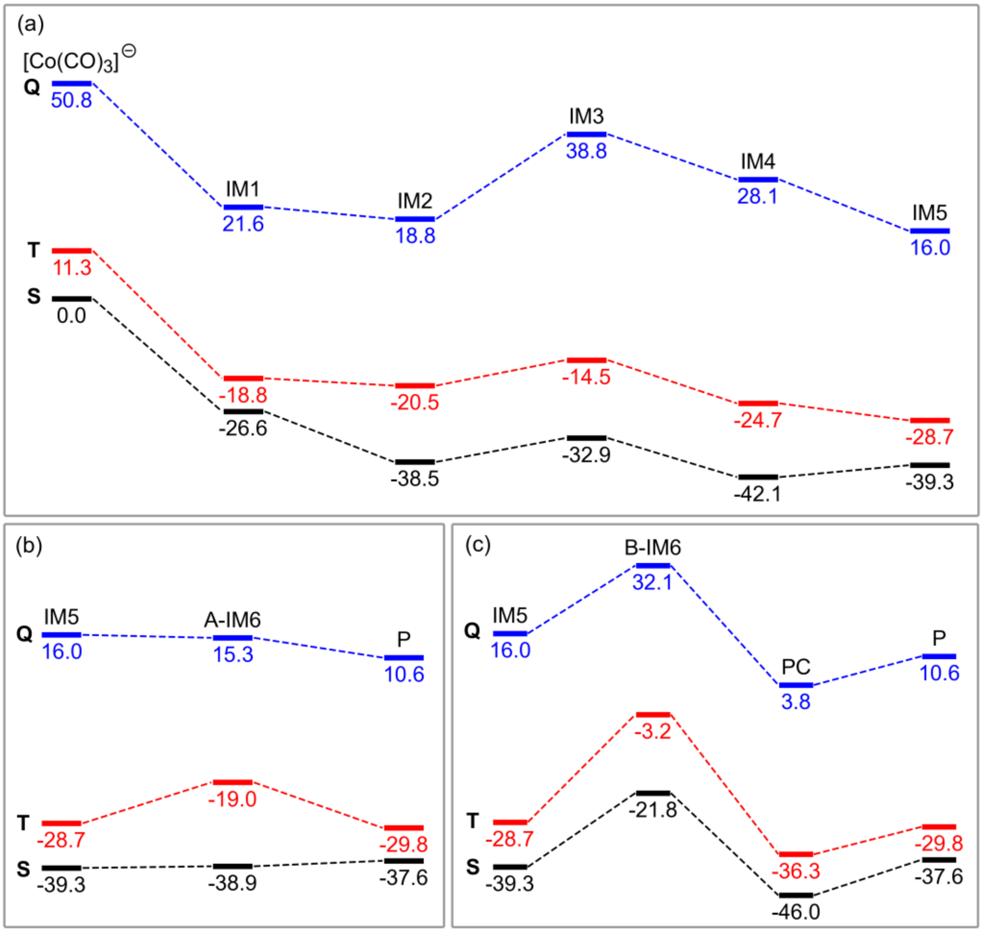

Figure S8. Free energy pathways on the singlet (S, black), triplet (T, red), and quintet (Q, blue) surfaces at the B3LYP-D3(BJ)/def2-TZVP(-f) level (a) up to IM5 formation (b) from IM5 through reductive elimination. (c) from IM5 through the amine-assisted nucleophilic substitution. All energies are given relative to the first point on the singlet potential energy surface. Chemical structures of intermediates are as shown on Figure S7.

2.4. An Alternative Nucleophilic Substitution Mechanism

As seen in Figure S9, the energies of the common intermediates for the catalytic cycle indicate no crossing among singlet, triplet, and quintet potential energies surfaces, and thus only the most stable singlet surface was explored in detail.

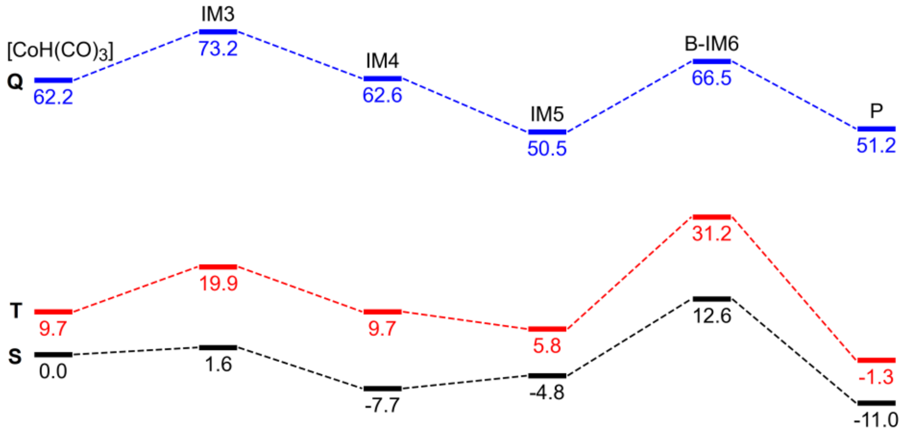

Figure S9. Free energy pathways on the singlet (S, black), triplet (T, red), and quintet (Q, blue) surfaces at the B3LYP-D3(BJ)/def2-TZVP(-f) level for an alternative nucleophilic substitution mechanism. All energies are given relative to the first point on the singlet potential energy surface. Chemical structures of intermediates are as shown on Figure S7.

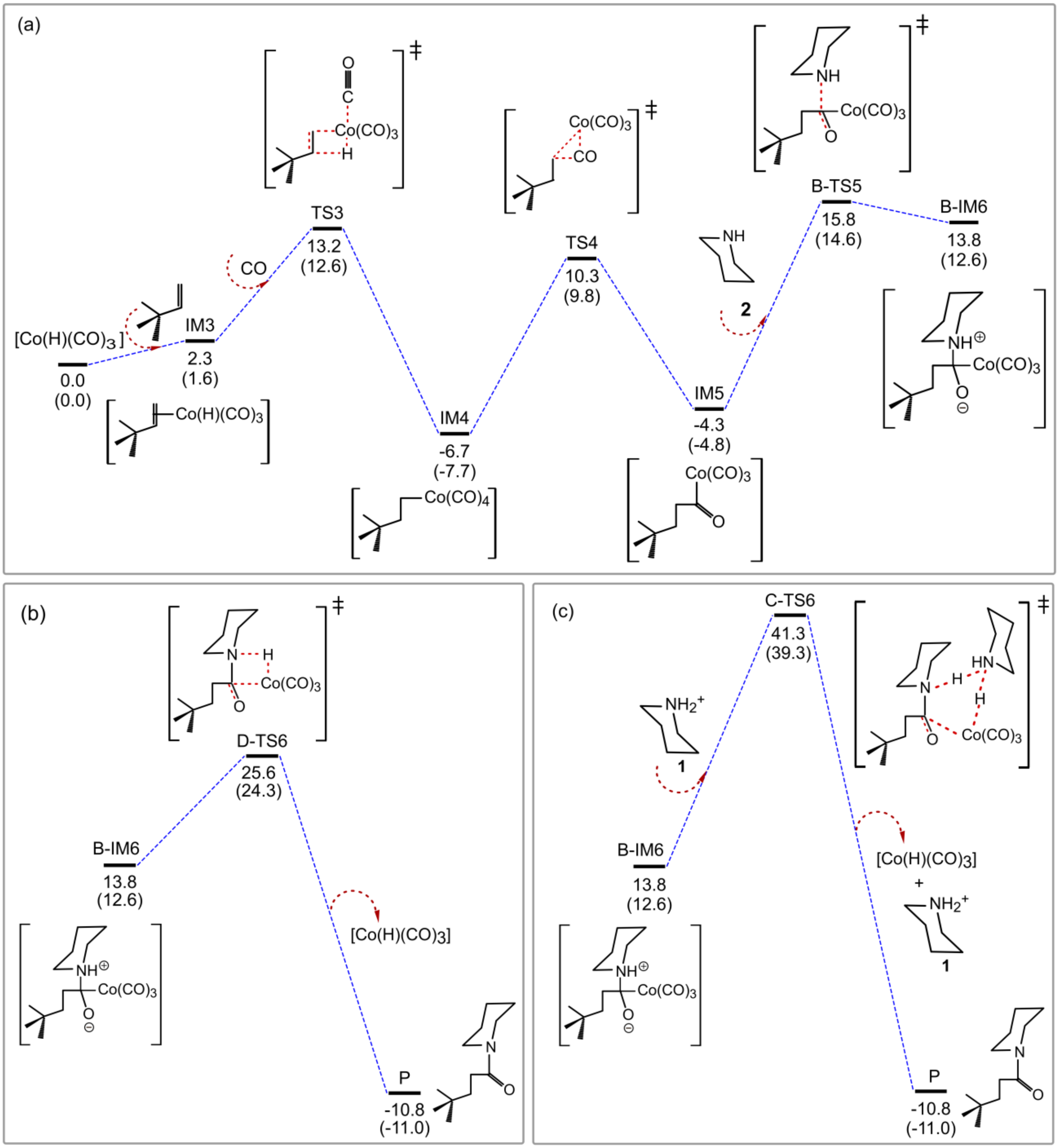

Figure S10. For the alternative mechanism, detailed free energy pathway on the ground singlet surface at the B3LYP-D3(BJ)/ma-def2-QZVP (out of parenthesis) and B3LYP-D3(BJ)/def2-TZVP(-f) (in parenthesis) levels (a) up to B-IM6 formation (b) from B-IM6 through direct nucleophilic substitution. (c) from B-IM6 through the protonated amine-assisted nucleophilic substitution. All energies are given relative to the first point on the potential energy surface.

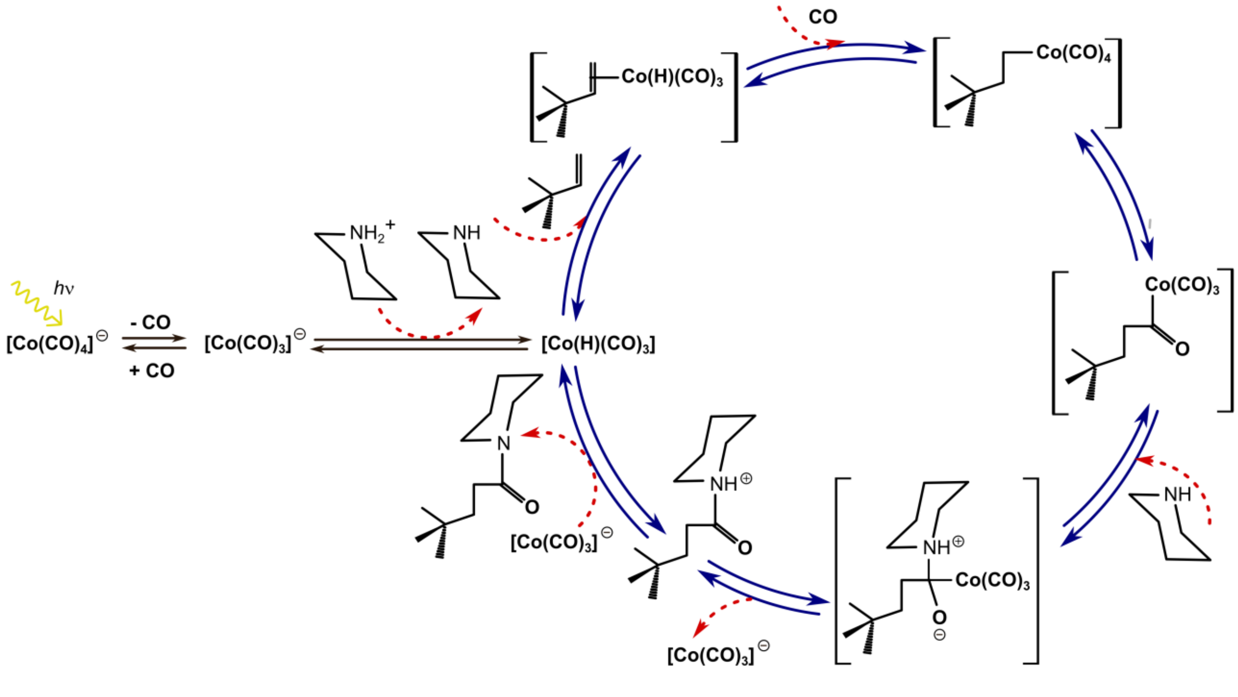

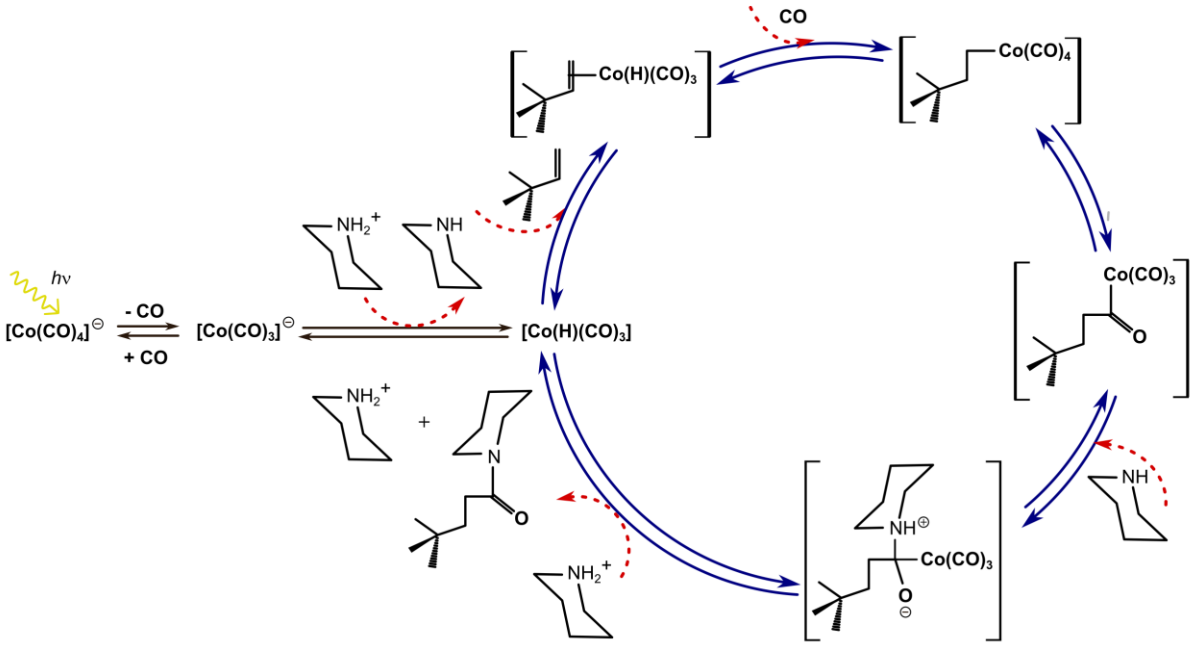

Figure S11. The alternative hydroaminocarbonylation catalytic cycle involving a SN catalyst release via (top) direct and (bottom) protonated amine-assisted nucleophilic substitution.

2.5. Nucleophilic Substitution with Different Substrates

Relaxed surface scans were carried out at the B3LYP-D3(BJ)/def2-TZVP(-f) level of theory to investigate the nucleophilic substitution from IM5 to the corresponding carboxylic acid or ester, using water or ethanol as the nucleophile. As shown in Figure S12 and Figure S13, the reaction is not favored in the absence of DMAP.

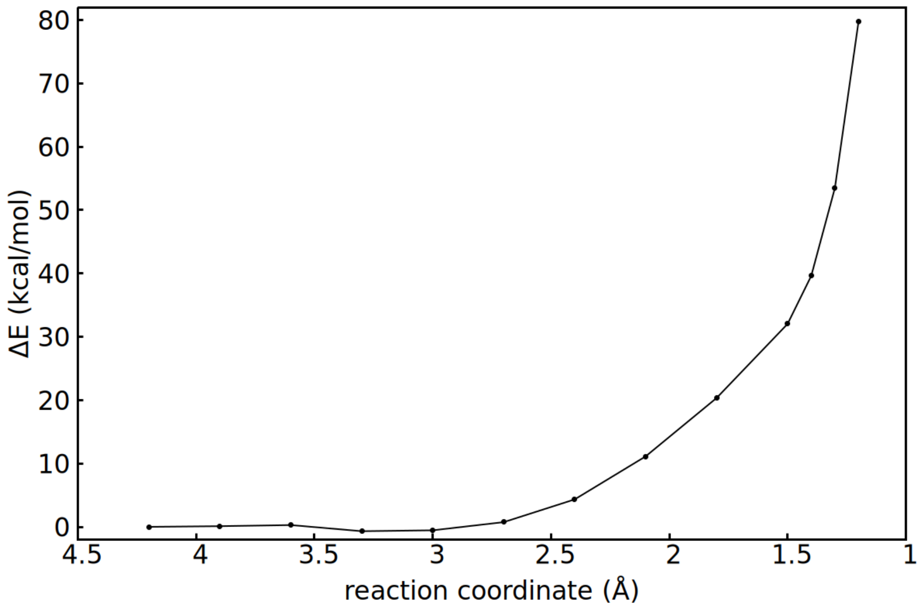

Figure S12. Relaxed surface scan along the O–C reaction coordinate, modeling the approach of water to the carbonyl carbon to form the corresponding bond.

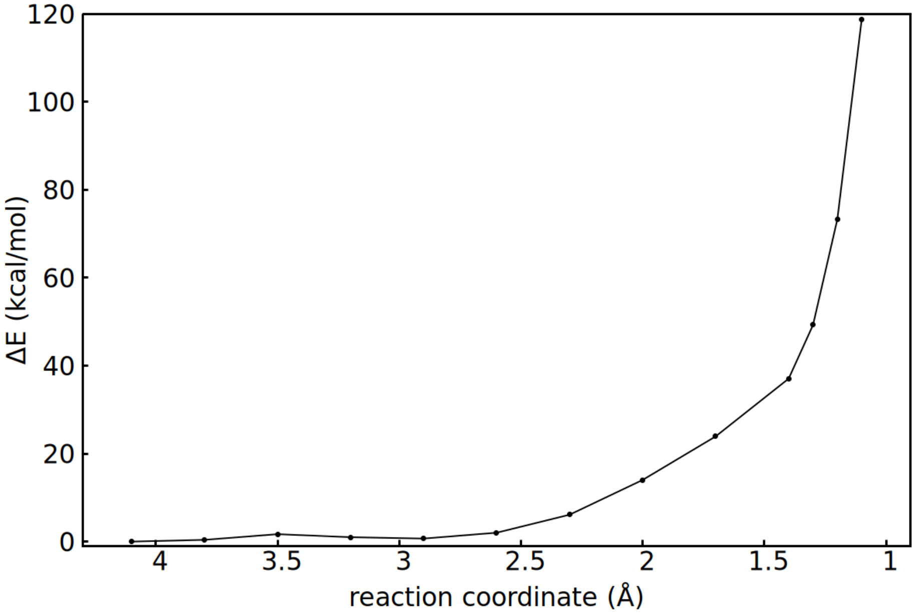

Figure S13. Relaxed surface scan along the O–C reaction coordinate, modeling the approach of EtOH to the carbonyl carbon to form the corresponding bond.

2.6. Alternative Pathways to Catalyst Formation

In addition to the originally proposed pathway, we explored the energetics of alternative mechanisms involving the pre-catalyst $[Co(CO)_4]^-$ to assess whether catalyst formation could proceed through a different route. In particular, we considered whether the tetracarbonyl complex might undergo proton transfer from a protonated amine prior to CO dissociation. We then considered whether coordination of an MTBE molecule might be thermodynamically favored, in which case attention should shift to the corresponding light-absorbing species as the most relevant intermediate.

2.6.1. Proton Transfer to $[Co(CO)_4]^-$

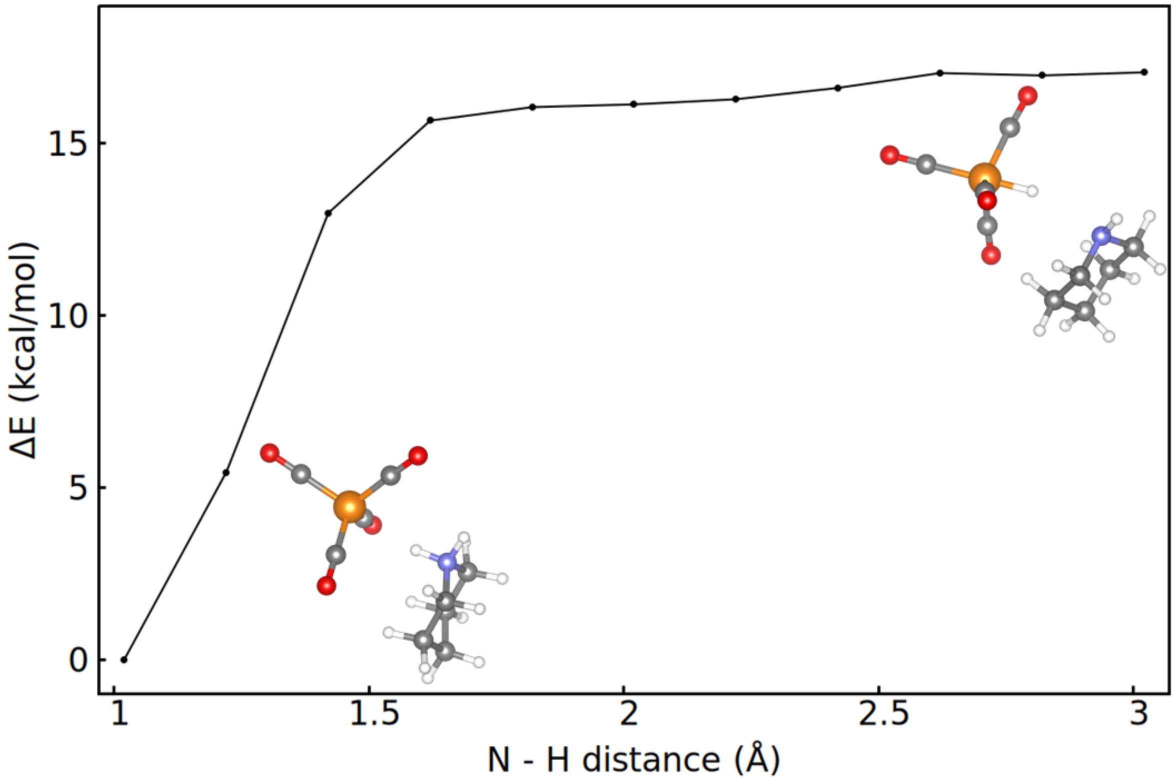

Figure S14. Relaxed surface scan performed on the N - H distance to model H transfer between the amine and the metal complex. Electronic energies were computed at B3LYP-D3(BJ)/ma-def2-QZVP-CPCM(MTBE) level of theory, while at each step the geometry was optimized at B3LYP-D3(BJ)/def2-TZVP(-f)-CPCM(MTBE) level. Energy values are reported in kcal/mol and are relative to the first structure.

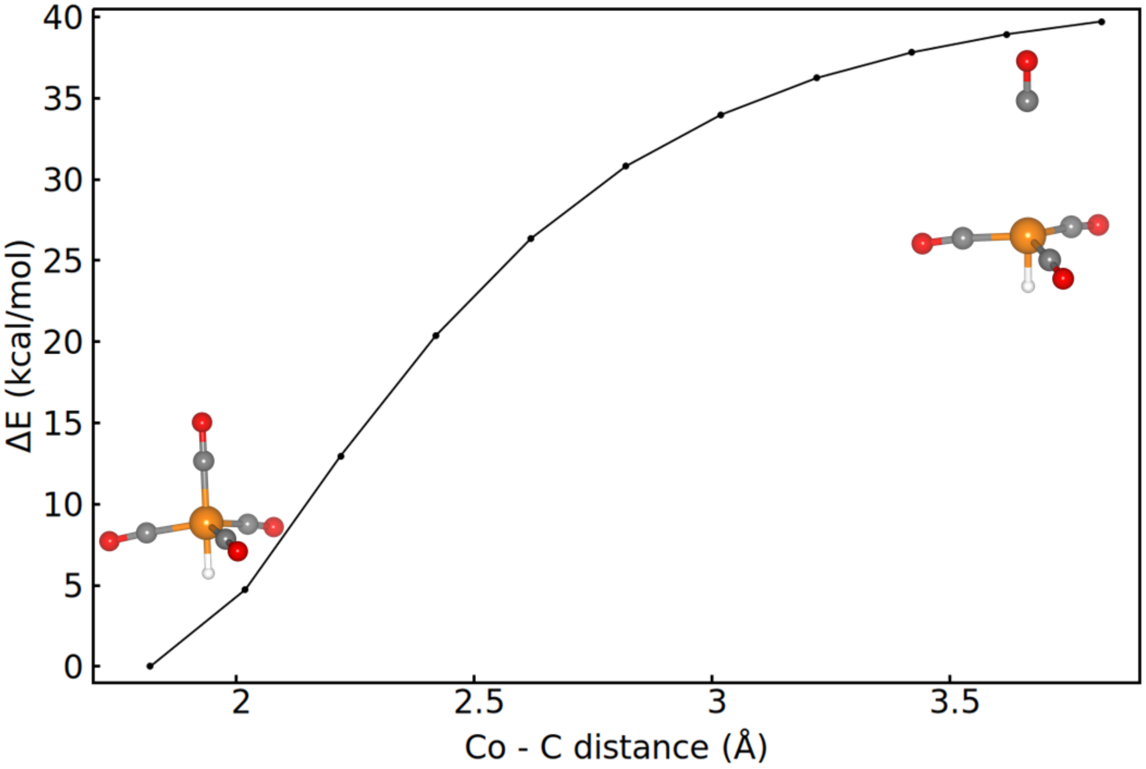

Figure S15. Relaxed surface scan performed on the Co - C distance to model thermal dissociation of CO from the metal complex. Electronic energies were computed at B3LYP-D3(BJ)/ma-def2-QZVP-CPCM(MTBE) level of theory, while at each step the geometry was optimized at B3LYP-D3(BJ)/def2-TZVP(-f)-CPCM(MTBE) level. Energy values are reported in kcal/mol and are relative to the first structure.

Figure S14 shows that, although not unfeasible, proton transfer to the pre-catalyst $[Co(CO)_4]^-$ is not favored, in agreement with Ref. 12 of the main paper. Additionally, Figure S15 shows that the resulting species $[Co(H)(CO)_4]$ does not exhibit an increased tendency toward CO dissociation. These results indicate that this alternative pathway would be significantly more energetically demanding and is therefore unlikely to contribute meaningfully to the formation of the active hydride species $[Co(H)(CO)_3]$.

2.6.2. Solvent Coordination to Metal Center

Figure S16 shows that solvent coordination to the Cobalt center is significantly disfavored. We therefore chose not to investigate further the effects of MTBE coordination on the system's properties.

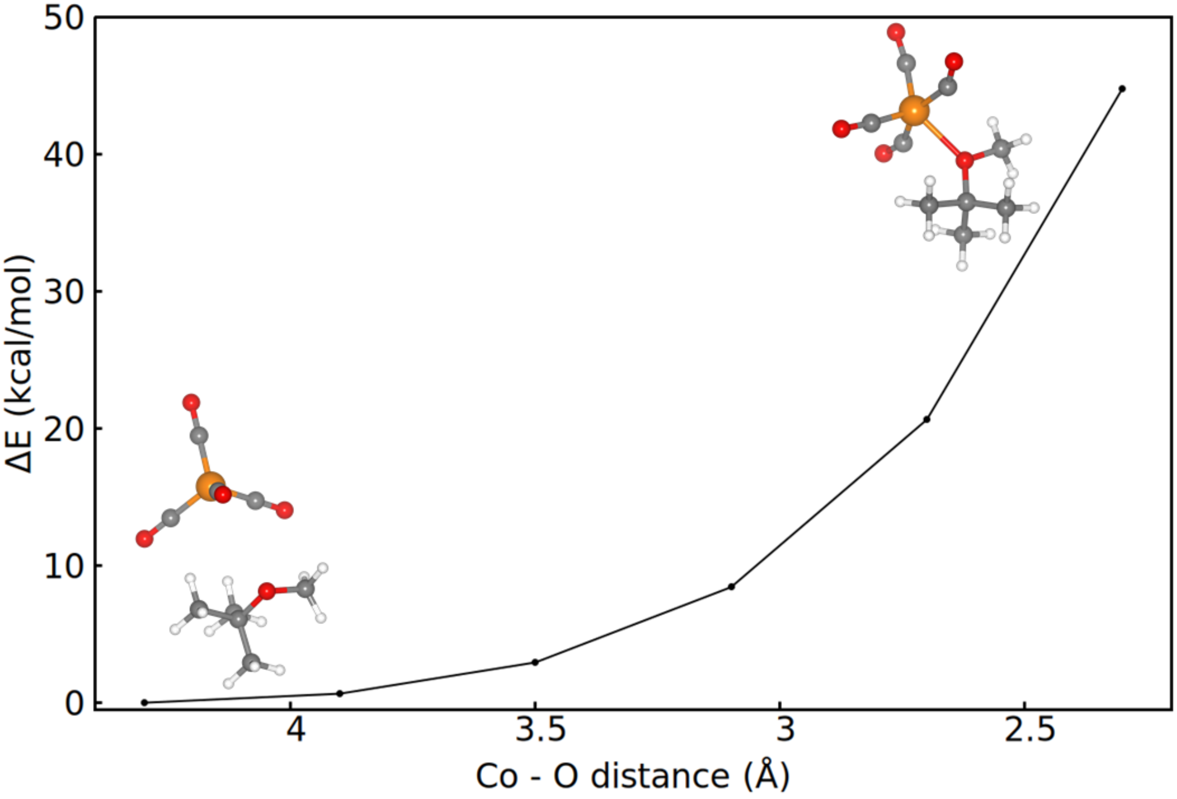

Figure S16. Relaxed surface scan performed on the Co - O distance to model the coordination of a solvent molecule (MTBE) to the Cobalt center. Electronic energies were computed at B3LYP-D3(BJ)/ma-def2-QZVP-CPCM(MTBE) level of theory, while at each step the geometry was optimized at B3LYP-D3(BJ)/def2-TZVP(-f)-CPCM(MTBE) level. Energy values are reported in kcal/mol and are relative to the first structure.

2.7. Optimized Cartesian Coordinates in Å

2.7.1. Equilibrium Geometries $[Co(CO_4)]^-$ in THF

Singlet (r_{Co-C} = 1.77 Å)

Co	0.00000000000000	0.00000000000000	0.00000000000000
C	1.02120232775966	-1.02120232775966	-1.02120232775966
O	1.68885268349404	-1.68885268349404	-1.68885268349404
C	-1.02120232775966	-1.02120232775966	1.02120232775966
O	-1.68885268349404	-1.68885268349404	1.68885268349404
C	1.02120232775966	1.02120232775966	1.02120232775966
O	1.68885268349404	1.68885268349404	1.68885268349404
C	-1.02120232775966	1.02120232775966	-1.02120232775966
O	-1.68885268349404	1.68885268349404	-1.68885268349404

Triplet (r_{Co-C} = 1.81 and 1.91 Å)

Co	-0.00326467684238	0.09058490937372	0.00582544714432
C	1.17161642935901	-0.85482748719455	-1.15794841070617
O	1.57771868333544	-1.87402445063745	-1.55559083182505
C	-1.17723510540211	-0.84459448437138	1.18132486179638
O	-1.57713577903813	-1.86365425520692	1.58638917165303
C	1.10169872008701	1.00274267708741	1.11102454177942
O	1.76267734024313	1.67723664319869	1.77075644862456
C	-1.11058441539764	0.98702747712357	-1.10983163514645
O	-1.77287219634433	1.65196897062691	-1.77788859332005

2.7.2. Equilibrium Geometries $[Co(CO_4)]^-$ in MTBE

Singlet (r_{Co-C} = 1.77 Å)

Co	0.00000000000000	0.00000000000000	0.00000000000000
C	1.02141079841605	-1.02141079841605	-1.02141079841605
O	1.68936942045717	-1.68936942045717	-1.68936942045717
C	-1.02141079841605	-1.02141079841605	1.02141079841605
O	-1.68936942045717	-1.68936942045717	1.68936942045717
C	1.02141079841605	1.02141079841605	1.02141079841605
O	1.68936942045717	1.68936942045717	1.68936942045717
C	-1.02141079841605	1.02141079841605	-1.02141079841605
O	-1.68936942045717	1.68936942045717	-1.68936942045717

Triplet (r_{Co-C} = 1.81 and 1.91 Å)

Co	0.02209805545970	0.14228071401965	-0.00142932845848
C	1.25530967224622	-0.70703036318583	-1.18793834018968
O	1.71030387319862	-1.69316342671990	-1.61482883926902
C	-1.12829238768752	-0.87056412001015	1.13342503332240
O	-1.50384006069295	-1.90648265214330	1.51594262602307
C	1.07532058484812	1.06451212289314	1.14369528305737
O	1.69925567611159	1.74773297446859	1.83158800262686
C	-1.10752353851212	1.02897212864285	-1.10146934910572
O	-1.78862887497165	1.69156862203495	-1.75439908800681

2.7.3. Singlet Potential Energy Surface of $[Co(CO_4)]^-$ in MTBE Along a Co–C Bond

r_{Co-C} = 1.57 Å

Co	0.00127258036735	0.00020783941811	-0.00116250079621
C	0.90772456849511	-0.90711481407441	-0.90670675440976
O	1.58280384863601	-1.58424876189936	-1.58349507610157
C	-1.02101444365451	-1.04246731317027	1.02113052570245
O	-1.64238439316224	-1.79205140506830	1.64197561841504
C	1.04429746981094	1.02157099351366	1.02202213788731
O	1.79360059275210	1.64163427646986	1.64451651297522
C	-1.02188223541453	1.02105279379448	-1.04461050437687
O	-1.64441798783021	1.64141639101624	-1.79366995929562

r_{Co-C} = 1.67 Å

Co	0.00281300047286	-0.00281356852175	-0.00280907860881
C	0.96698446427506	-0.96698630669963	-0.96698972189904
O	1.63877587219232	-1.63878303746728	-1.63878912417973
C	-1.02036933971376	-1.03264659182524	1.02037323707716
O	-1.66854189274401	-1.73660288502004	1.66854424020038

C	1.03264307148250	1.02037336908603	1.02037296493529
O	1.73659740619061	1.66854763387673	1.66854516474773
C	-1.02036726578069	1.02037077624115	-1.03264296269196
O	-1.66853531637489	1.66854061033002	-1.73660471958103

r_{Co-C} = 1.87 Å

Co	-0.00517585785126	0.00517873973526	0.00517380880263
C	1.07447449470084	-1.07446147460364	-1.07447063403951
O	1.73918860431952	-1.73916517777060	-1.73918062314663
C	-1.02504085282730	-1.01189395921094	1.02503639013761
O	-1.70915948009382	-1.64798395315318	1.70915431182913
C	1.01191001615012	1.02502948256939	1.02504032916600
O	1.64801499028177	1.70913282204631	1.70916025140272
C	-1.02504535642638	1.02502881037342	-1.01190546934951
O	-1.70916655825347	1.70913471001397	-1.64800836480243

r_{Co-C} = 1.97 Å

Co	-0.01186896803746	0.01536356112549	0.01387099716775
C	1.12564603426764	-1.12177442729020	-1.12361606071821
O	1.78640419776756	-1.78471830642870	-1.78608085374704
C	-1.03096407387440	-1.00031850742895	1.03056084804283
O	-1.73073093867952	-1.61172078694124	1.72285285153966
C	1.00706705004879	1.03100053333525	1.03174718599871
O	1.61997212778122	1.72320631604981	1.73021461532770
C	-1.03188967490733	1.02942215973327	-1.00399316199070
O	-1.73363575436648	1.71953945784529	-1.61555642162068

r_{Co-C} = 2.07 Å

Co	-0.02950436673899	0.03706660398293	0.03341671455586
C	1.16390884581798	-1.15970290691012	-1.16174337848116
O	1.82150911740189	-1.82128413280321	-1.82179123936667
C	-1.03966466207550	-0.99182013204294	1.04167664404161
O	-1.74412897392945	-1.60483611955262	1.72926238088705
C	1.00339886826589	1.04428495807419	1.04222010815676
O	1.61784971616008	1.73118812315190	1.74591869563391
C	-1.04325489898448	1.04036069946654	-0.99761003095283
O	-1.75011364591741	1.72474290663333	-1.61134989447453

r_{Co-C} = 2.17 Å

Co	-0.03705009429144	0.03960467828821	0.03839339423322
C	1.21692655712470	-1.21227888174999	-1.21429575651821
O	1.87396352876217	-1.87090877611656	-1.87195591911760
C	-1.04663793683137	-0.98596234421015	1.04537527524751
O	-1.76109514703643	-1.56471843305904	1.75348792021178
C	0.99224817876465	1.04543317759626	1.04648660320029
O	1.57281330504838	1.75358727820280	1.75934517113556
C	-1.04736949982190	1.04456521354032	-0.98906111222104
O	-1.76379889171876	1.75067808750814	-1.56777557617153

r_{Co-C} = 2.27 Å

Co	-0.05441108126013	0.05720959706492	0.05600525650288
C	1.25638579441294	-1.25318016004384	-1.25456340845799
O	1.91145663689155	-1.91044331148018	-1.91074308491817
C	-1.05613777473803	-0.98115615286941	1.05536710495258
O	-1.77255281807993	-1.55582426920604	1.76602015014108
C	0.98586184778001	1.05646342106737	1.05697263234069
O	1.56159205498506	1.76745107551472	1.77211850860640
C	-1.05708331917971	1.05533780147776	-0.98314311796988
O	-1.77511134081176	1.76414199847469	-1.55803404119760

r_{Co-C} = 2.37 Å

Co	-0.07210588809759	0.07477179137252	0.07363016928266
C	1.29610258474617	-1.29363500945323	-1.29471496584612
O	1.94972096671295	-1.94960306708510	-1.94954841222073
C	-1.06630532735720	-0.97510027445879	1.06577311525293
O	-1.78392168760204	-1.54708736985116	1.77849420776945
C	0.97883635887942	1.06712255085069	1.06743683772755
O	1.55121792673083	1.78032818292363	1.78418814246953
C	-1.06724803879109	1.06600670794978	-0.97660100573050
O	-1.78629689522145	1.77719648775166	-1.54865808870476

r_{Co-C} = 2.47 Å

Co	-0.09047495268780	0.09283766132012	0.09181365671496
C	1.33538985693844	-1.33337920076578	-1.33427014338867

O	1.98788260564029	-1.98820463027919	-1.98797132282972
C	-1.07712415245224	-0.96868312721619	1.07672265289419
O	-1.79502252269624	-1.54004780965358	1.79065671257774
C	0.97157714478357	1.07809114975540	1.07832370228928
O	1.54292510266969	1.79253914044947	1.79566925897988
C	-1.07800859543570	1.07706207248516	-0.96982692163721
O	-1.79714448676001	1.78978474390457	-1.54111759560044

r_{Co-C} = 2.57 Å

Co	-0.10954673308701	0.11156307189232	0.11068513143128
C	1.37407559766826	-1.37237000370151	-1.37313001725805
O	2.02567817624862	-2.02622342931688	-2.02590974978709
C	-1.08846770022395	-0.96193614871063	1.08814468791459
O	-1.80575249264383	-1.53486926268607	1.80229924873801
C	0.96409273407626	1.08945759287915	1.08964299726560
O	1.53678280338835	1.80410579982205	1.80658806097982
C	-1.08926802700474	1.08852584354404	-0.96277600436742
O	-1.80759435842195	1.80174653627753	-1.53554435491674

r_{Co-C} = 2.67 Å

Co	-0.12929524363191	0.13102124113890	0.13025694375896
C	1.41209981656247	-1.41061585471645	-1.41128654896464
O	2.06299861081877	-2.06371114675065	-2.06331752354948
C	-1.10040245577916	-0.95515480867035	1.10014925130151
O	-1.81619703282196	-1.53187357354338	1.81346632698594
C	0.95672301591560	1.10141106184197	1.10155365651329
O	1.53304467789273	1.81518967272454	1.81717631857465
C	-1.10114337477128	1.10056934239150	-0.95575477131691
O	-1.81782801418525	1.81316406558392	-1.53224365330331

r_{Co-C} = 2.77 Å

Co	-0.15033606128880	0.15170107318304	0.15108246193692
C	1.44886681702887	-1.44760385831466	-1.44819046262842
O	2.09933715646062	-2.10024338805700	-2.09974157404493
C	-1.11294081583623	-0.94810576433098	1.11276853017699
O	-1.82564532699542	-1.53203909472650	1.82374282465317
C	0.94897945246574	1.11396044438732	1.11404296448922
O	1.53235855855788	1.82536158252386	1.82675294200045
C	-1.11360493858537	1.11323109121457	-0.94841494697838
O	-1.82701484180728	1.82373791412034	-1.53204273960501

r_{Co-C} = 2.87 Å

Co	-0.17496053997361	0.17575659214028	0.17534913523420
C	1.48209518207786	-1.48116909390671	-1.48165526781036
O	2.13198809151426	-2.13340272846456	-2.13257853054280
C	-1.12707522884122	-0.94119045163077	1.12709404251312
O	-1.83245554089698	-1.54181702516166	1.83191994451581
C	0.94086767907640	1.12822685235897	1.12816488750668
O	1.54064942648187	1.83344015779904	1.83386704692972
C	-1.12765065179332	1.12769163536513	-0.94099026531777
O	-1.83345841764524	1.83246406150027	-1.54117099302860

r_{Co-C} = 2.97 Å

Co	-0.19416266280282	0.19508662337194	0.19460500957568
C	1.52057744603233	-1.51963744342084	-1.52012177152025
O	2.16975802612148	-2.17180815674498	-2.17067320106501
C	-1.13956192871090	-0.93302820515064	1.13966388608887
O	-1.84271671270030	-1.53924584568990	1.84230673775358
C	0.93249777420406	1.14103382847282	1.14089493833207
O	1.53768786931277	1.84412557201247	1.84448348456980
C	-1.14023291923614	1.14041520391569	-0.93273854735171
O	-1.84384689222048	1.84305842323343	-1.53842053638303

r_{Co-C} = 3.07 Å

Co	-0.21439794018960	0.21547722537919	0.21490869968333
C	1.55804593003816	-1.55701406237530	-1.55755219397721
O	2.20653108076358	-2.20937175520720	-2.20782641348025
C	-1.15255826797404	-0.92449468528380	1.15273383705429
O	-1.85207173668301	-1.53936509469761	1.85175699518888
C	0.92378990390549	1.15439200651761	1.15418210520664
O	1.53739194844536	1.85395981083005	1.85426217307269
C	-1.15334905383322	1.15366716594924	-0.92412796165651
O	-1.85338186447272	1.85274938888782	-1.53833724109187

$r_{Co\text{-}C}$ = 3.17 Å

Co	-0.23483578980372	0.23612177971436	0.23543185003460
C	1.59535210607742	-1.59411627813389	-1.59474333537598
O	2.24303131103745	-2.24690034086579	-2.24480459042884
C	-1.16579492565029	-0.91556361285563	1.16604236709409
O	-1.86100284114630	-1.54033711864205	1.86075184275976
C	0.91467734035529	1.16807167926595	1.16777874591196
O	1.53789546255254	1.86345120689252	1.86370713275280
C	-1.16675062877786	1.16722221445118	-0.91510430374459
O	-1.86257203464452	1.86205047017334	-1.53905970900380

$r_{Co\text{-}C}$ = 3.27 Å

Co	-0.25517165118972	0.25674059815716	0.25590694512236
C	1.63277257265149	-1.63120421047444	-1.63201021855838
O	2.27945209272014	-2.28471346870437	-2.28189139532386
C	-1.17912557833016	-0.90627940906832	1.17946374479177
O	-1.86966958302221	-1.54145300317953	1.86946328920763
C	0.90516547480923	1.18198662618491	1.18159264191005
O	1.53841682253500	1.87284128213264	1.87304130729577
C	-1.18028313124061	1.18094443428636	-0.90570096602222
O	-1.87155701893314	1.87113715066559	-1.53986534842313

$r_{Co\text{-}C}$ = 3.37 Å

Co	-0.27525575649954	0.27720989874833	0.27617535779757
C	1.67044897359183	-1.66843427724695	-1.66948704408008
O	2.31587155121652	-2.32299382462696	-2.31919885170059
C	-1.19243766378219	-0.89665525628488	1.19289959980898
O	-1.87811804786773	-1.54240042054938	1.87797219314755
C	0.89524571421639	1.19608517154955	1.19556128656152
O	1.53856719205822	1.88226045318415	1.88240984799599
C	-1.19386869052455	1.19478439089393	-0.89592850751208
O	-1.88045327240894	1.88014386433221	-1.54040388201887

$r_{Co\text{-}C}$ = 3.47 Å

Co	-0.29485527197871	0.29724319414536	0.29598346779649
C	1.70869662359814	-1.70601604588348	-1.70742173857016
O	2.35257856850655	-2.36196064762988	-2.35699172439162
C	-1.20571443399272	-0.88667817918226	1.20631787315404
O	-1.88656962752478	-1.54259438571067	1.88648871862612
C	0.88491730899739	1.21026782803328	1.20960053248345
O	1.53784312815639	1.89183183372096	1.89192681199598
C	-1.20747084759281	1.20867075177089	-0.88577340329477
O	-1.88942544816945	1.88923565073579	-1.54013053779951

$r_{Co\text{-}C}$ = 3.57 Å

Co	-0.31397778997926	0.31690391932405	0.31534489653322
C	1.74753888633795	-1.74388072638919	-1.74577515642988
O	2.38944921180023	-2.40170294863754	-2.39518702568966
C	-1.21886624869600	-0.87642063613495	1.21961196168901
O	-1.89500752898193	-1.54207958838577	1.89496355250220
C	0.87424462128936	1.22452224444790	1.22367309548052
O	1.53622945523722	1.90162047119753	1.90166100470810
C	-1.22105054577917	1.22257731600036	-0.87528696173506
O	-1.89856006122841	1.89845994857761	-1.53900536705844

$r_{Co\text{-}C}$ = 3.67 Å

Co	-0.33184815942665	0.33533256745831	0.33347131186443
C	1.78766431770989	-1.78292683829102	-1.78538315795163
O	2.42747738090660	-2.44276948045101	-2.43464924223563
C	-1.23189300581671	-0.86573103498818	1.23280371171197
O	-1.90403210641180	-1.53931098350385	1.90404314876425
C	0.86311910024877	1.23869906608607	1.23767170548429
O	1.53231770057247	1.91204636309148	1.91205582881168
C	-1.23451281604424	1.23638098554937	-0.86437195311561
O	-1.90829241173834	1.90827935504883	-1.53564135333375

$r_{Co\text{-}C}$ = 3.77 Å

Co	-0.35026460411837	0.35438695851597	0.35217729189315
C	1.82750589432662	-1.82109043459983	-1.82440581069240
O	2.46443312249832	-2.48374546202112	-2.47347879880278
C	-1.24478818472457	-0.85506747137266	1.24588431129724
O	-1.91245288347225	-1.53769313438683	1.91253109983270
C	0.85188239184589	1.25299941296856	1.25174306848705
O	1.52922498602462	1.92224310446094	1.92216467914538

C	-1.24794297385190	1.25022995410361	-0.85339765483069
O	-1.91759774852837	1.91773707233137	-1.53321818632964

r_{Co-C} = 3.87 Å

Co	-0.36879114586555	0.37344015341992	0.37093872147090
C	1.86788575884170	-1.85857713059350	-1.86340135680841
O	2.50093883192550	-2.52514548348489	-2.51227964803129
C	-1.25803806461708	-0.84427800282769	1.25930405510161
O	-1.92065680977240	-1.53646986816870	1.92083247693170
C	0.84048683005939	1.26754907084338	1.26610004346238
O	1.52651650205715	1.93216482545908	1.93199734217488
C	-1.26170507031506	1.26435264462366	-0.84228912092696
O	-1.92663683231364	1.92696379072873	-1.53120251337481

r_{Co-C} = 3.97 Å

Co	-0.38634213257893	0.39161088181161	0.38877671896146
C	1.90950031729313	-1.89667470640998	-1.90333079009246
O	2.53788666620913	-2.56781463763511	-2.55192038824118
C	-1.27089877119972	-0.83318732831636	1.27234441763568
O	-1.92928921887113	-1.53342860455424	1.92953168161487
C	0.82869620728860	1.28189383547678	1.28023286890040
O	1.52181790420478	1.94271279927375	1.94246201402048
C	-1.27515201617193	1.27819284980134	-0.83082327757772
O	-1.93621895617392	1.93669491055221	-1.52727324522153

r_{Co-C} = 4.07 Å

Co	-0.40322572655325	0.40911738984566	0.40596494720447
C	1.95191127396434	-1.93527479419130	-1.94394044073089
O	2.57522203372398	-2.61117393503968	-2.59214167122268
C	-1.28356611352887	-0.82196411032832	1.28521177694243
O	-1.93828999867559	-1.52905974037682	1.93861133707438
C	0.81674690724366	1.29606969248195	1.29420749990219
O	1.51574665899534	1.95367544465210	1.95335583953203
C	-1.28838953173787	1.29183092572941	-0.81923212616300
O	-1.94615550343174	1.94677912722700	-1.52203716253892

r_{Co-C} = 4.17 Å

Co	-0.41966228774888	0.42631665316568	0.42278175151950
C	1.99524524458498	-1.97370369668191	-1.98491929542744
O	2.61220681990662	-2.65577821715961	-2.63283482657130
C	-1.29609086867702	-0.81072864070682	1.29798644924160
O	-1.94726963433362	-1.52439750428023	1.94769120529414
C	0.80458616725943	1.31049104027848	1.30838312967358
O	1.50890239237462	1.96513257651041	1.96472254902493
C	-1.30162455572944	1.30556131428331	-0.80752848526402
O	-1.95629327763669	1.95710647459069	-1.51628247749099

r_{Co-C} = 4.27 Å

Co	-0.43432886153390	0.44159502836234	0.43774398104155
C	2.03966997047492	-2.01479629964708	-2.02769142342065
O	2.65188721328572	-2.70075757825608	-2.67510253346253
C	-1.30867002659386	-0.79798786612844	1.31073129727859
O	-1.95732895242564	-1.51633294107700	1.95777889562121
C	0.79125433986238	1.32437565896145	1.32209646309362
O	1.49942293169489	1.97686000757430	1.97642522623254
C	-1.31471142853116	1.31898107681006	-0.79449022170495
O	-1.96719518623334	1.96806291340043	-1.50749168467938

r_{Co-C} = 4.37 Å

Co	-0.44955302131102	0.45733231975051	0.45323680653562
C	2.08369236712619	-2.05527545445374	-2.06992992366198
O	2.69123493358750	-2.74548921313985	-2.71716857924858
C	-1.32127468560661	-0.78618605132749	1.32350500711729
O	-1.96755938417577	-1.50884183678808	1.96805747084943
C	0.77886182595804	1.33822897524134	1.33578695161656
O	1.49053138573643	1.98872498549439	1.98823041264407
C	-1.32776460976773	1.33236164626885	-0.78239791086720
O	-1.97816881154704	1.97914462895406	-1.49932023498519

r_{Co-C} = 4.47 Å

Co	-0.46432814098579	0.47259226339598	0.46827580815633
C	2.12828612814358	-2.09612659124326	-2.11260292821771
O	2.73095158821892	-2.79065372555224	-2.75972418330554
C	-1.33386357969857	-0.77417126347071	1.33626140290187

O	-1.97809347757364	-1.50054428597668	1.97864274682092
C	0.76628693490392	1.35199883682284	1.34942140936963
O	1.48090253658379	2.00080675698352	2.00027782680871
C	-1.34076038304230	1.34565763040312	-0.77013099386718
O	-1.98938160654990	1.99044037863742	-1.49042108866703

2.7.4. Geometries $[Co(CO_3)]^-$ in MTBE

Singlet

Co	0.17105268920345	-0.17160101813674	0.16986399551373
C	1.04014043381735	-1.04057781329148	-1.07793296811470
O	1.68202303072711	-1.68152677495759	-1.80730880675690
C	-1.07996302545774	-1.03738297663991	1.03699938556483
O	-1.81209033454925	-1.67601366353454	1.67805958122450
C	1.03529991347059	1.08153255977379	1.03585553598252
O	1.67431751166170	1.81478946791325	1.67524349545923

Triplet

Co	0.38750204436101	-0.38635492067889	0.38891479736616
C	1.09566912949961	-1.11283678885294	-1.09342007500086
O	1.54849757944683	-1.57940362109404	-2.05800404378398
C	-1.04126317741537	-1.14921679161608	1.16461087933918
O	-1.96671306353765	-1.64450839328565	1.66617101010925
C	1.10789400489956	1.10099494840845	1.09191945879120
O	1.57919370161921	2.06054534824594	1.55058819205227

MECP

Co	0.38719678618992	-0.38690454203393	0.38816335993266
C	1.16237009879449	-1.18120713818512	-1.23728818646830
O	1.61921487169523	-1.64885382171363	-2.20084898879093
C	-1.17805151833668	-1.22222590985666	1.23962145514349
O	-2.10462246091803	-1.71609343374315	1.74301552467515
C	1.17752209259846	1.24127910212310	1.15995363723180
O	1.64715034884980	2.20322552453619	1.61816341714935

2.7.5. Reaction Mechanisms

3,3-dimethyl-1-butene

C	-1.39966326847985	1.18137883296429	1.36207749490480
H	-0.50706965824795	1.57949753211078	-2.03877330018891
C	-0.27899341379912	1.42973528813275	0.69456483240942
H	1.02181055379238	2.29068097713335	-1.50406636532385
H	-1.97088350096756	0.27237566117733	1.22015920013820
H	-1.78721100167667	1.89130752957365	2.08266277514507
C	0.39421900001344	0.55459059899978	-0.33653959882708
H	1.02458685156076	0.77048003893054	-2.40819216627573
H	0.24282667380871	2.36523569182665	0.88877035175458
C	0.48797824464949	1.34939103550671	-1.65276208283139
H	2.35732408387213	-0.35024980265561	-0.58820996859570
H	2.38240648622711	1.15602508804958	0.33807407412338
C	1.81805479208658	0.23818620823004	0.15793967812651
H	1.78942984009858	-0.33103638946968	1.08923009041813
H	-1.37298621357769	-0.56377716236188	-0.95029343667372
C	-0.36201338686044	-0.75270375069762	-0.58401657692724
H	0.16119420861718	-1.34902620017104	-1.33427093341473
H	-0.43555629111708	-1.34865917727964	0.32782293203826

Piperidine

C	-4.29507900294890	1.07933584553717	0.27782899608885
C	-4.25340429225459	-0.34013294559287	-0.29436364889958
H	-4.48476172614087	1.03583036654851	1.35469997478836
H	-2.97712840836786	2.78204485514229	0.50774914820844
H	-2.83298000853337	1.94314922789860	-1.04428459074185
N	-1.87137396190301	1.01581287682254	0.62283590424879
H	-1.57881275022388	-0.22835239114284	-1.08907707557388
H	-0.89757881568867	-0.82039637719209	0.43375477242343
C	-3.03507106942839	-1.10305477603786	0.23234834560042
H	-5.17442959826471	-0.87707363550934	-0.05443636215509
H	-4.19481440900138	-0.28326027403667	-1.38733178871818
C	-1.75572879382291	-0.30448505526289	-0.00189785214627
H	-3.14857905503535	-1.27892183971393	1.30648838375509

H	-2.95412396684625	-2.07913021394923	-0.25280734381900
C	-2.96800533256327	1.79468379692333	0.04145425425375
H	-5.10791908941384	1.65245986725812	-0.17547325147424
H	-1.00073371956274	1.52147567230715	0.50896613416094

Protonated piperidine

C	-4.28328279475284	1.06698389510149	0.28644826803544
C	-4.25041043121189	-0.34549362355100	-0.29999334302890
H	-4.46638553656037	1.02283033301766	1.36455308154450
H	-2.94795917975141	2.77856961154622	0.51736440955804
H	-2.79591179904620	1.93865165226167	-1.03802440736139
N	-1.81764556824030	1.00435960699808	0.55887528664666
H	-1.56010267322308	-0.25337889918470	-1.09401326607505
H	-0.88614881399418	-0.87717538123728	0.42407283025735
C	-3.04651197305455	-1.12561033081057	0.23069925564047
H	-5.17241884410660	-0.87212276565529	-0.05339749796619
H	-4.19603659542385	-0.28693015987056	-1.39113568716723
C	-1.74546491873859	-0.38633625031609	-0.02859800530531
H	-3.15551483742195	-1.30055966228504	1.30558827611814
H	-2.97481330177810	-2.10532665503824	-0.24330574336297
C	-2.98257701043379	1.80752458557640	0.02740287828130
H	-5.09383846918177	1.65305181476587	-0.14848118525957
H	-1.89568845921801	0.93402937445807	1.57595732477820
H	-0.94530479386249	1.50096385422332	0.37242152466652

Carbon monoxide

C	-3.31649876823340	-0.70240454982173	0.00000000000000
O	-2.19105123176660	-0.70966545017827	0.00000000000000

$[Co(CO)_3]^-$

Co	-0.39663881242688	0.24810871280990	0.00000254335374
C	-0.44016794568997	2.00491227663493	-0.00000184515486
O	-2.89428394835705	-1.27249489664017	-0.00000131275774
O	2.17996306272327	-1.13353624461739	-0.00000130167501
O	-0.46764432265851	3.16946831014264	-0.00000332603406
C	1.15208797795810	-0.58568102754177	-0.00000088668550
C	-1.89834801154897	-0.66859113078815	-0.00000087104656

$[Co(H)(CO)_3]$

H	-0.00023924171140	1.78471372498733	0.07711326142961
Co	0.00014803861554	0.27735716961974	-0.01785132062319
C	1.75708715673276	0.61687444612643	-0.00642836025976
O	2.85677002959857	0.90654173926729	0.00246113233919
C	-0.00020222058844	-1.52047278180600	-0.13386852918353
O	-0.00030989798916	-2.65647852965522	-0.20687907851017
C	-1.75676582512864	0.61663531804451	-0.00641288215052
O	-2.85648901051722	0.90614400372891	0.00247837002536

Amide product (4,4-dimethyl-1-(piperidin-1-yl)pentan-1-one)

H	-1.90478263404349	-0.49803108336990	-5.03658688439722
H	1.87807898031465	-0.69155452457107	-4.53119767328342
H	4.87028834540690	-1.98422660349999	-2.28151282955193
C	4.86828595001560	-1.93753624787904	-1.18936112022898
H	0.23772665562297	-0.66660191172157	-6.37624547910584
H	-0.56631755008652	0.34944117538925	-4.26553963245026
C	4.23649546981025	-0.59587617177109	0.83477058870342
H	3.75582467102243	0.31280715094553	1.20545008230076
H	2.03895790901622	-2.18690071730974	-5.45016361382623
C	-0.93564587616466	-0.63983480922612	-4.55228153632878
H	5.90296074841373	-2.01734152737846	-0.84796032542948
H	4.33011249931859	-2.80951725532819	-0.81343880894877
C	0.05420557981363	-1.30593729817648	-5.50982671604152
O	-0.40957261884982	-0.60681436984084	-0.77950057227549
H	5.25914728305666	-0.61808569667571	1.21840344426673
H	6.10675534893419	0.48350806155208	-0.86387052836188
H	5.09637110365886	0.56296324511959	-2.31288914955482
C	1.79140099619682	-1.51980002856246	-0.79850566233566
H	2.05254857248765	-2.60282528900179	-2.99100729740371
C	2.81079004201665	-0.46714291329422	-1.25720388764806
H	4.66232212517488	1.50347608884401	-0.87988753372073
H	1.64613689423484	-1.45136374386221	0.27763990268607
H	2.14239284179240	-2.52699656250721	-1.02027692960566
C	4.24033731979499	-0.62616888978658	-0.69894901525881
H	2.87327531956634	-0.46926223511798	-2.34881879624180
H	2.43081907750217	0.51795045994428	-0.97155879310384

C	5.07620161055598	0.55120448285780	-1.22016724600070
H	3.70684277580493	-1.45276554320911	1.25426317170062
N	0.16580588070264	-1.74952421926111	-2.65100807615320
H	-1.58152800151631	-2.44001719457495	-3.54860628299180
H	-1.75514456870777	-0.97832679644123	-2.56413033826113
C	1.36928854179601	-1.62320242251717	-4.79593244589179
C	1.12233534278578	-2.43020827356099	-3.52004716941888
H	-0.38258804606971	-2.23680601728424	-5.88894448741746
H	0.70754737025716	-3.41019565071955	-3.78353064477264
C	-1.12225343105895	-1.47921549231477	-3.28953096437374
C	0.42503347142328	-1.26162717588876	-1.40848674927330

IM1

C	-4.76468268874592	1.34138564593284	-0.13557425634697
C	-4.74545840823822	-0.10003373908176	-0.64729113482111
H	-5.51818385896267	1.44792029968578	0.64776845447289
H	-3.43704620896158	2.73537847523835	0.88286570864657
H	-2.64337537880818	1.74730084110582	-0.35905774623113
N	-2.96555750564781	0.78346290086720	1.47186484703213
H	-2.13892093497623	-0.69225058939076	0.23812787256088
H	-2.61799223750696	-1.24405775014846	1.85493867068458
C	-4.26911281471728	-1.05702067748385	0.44693549911558
H	-5.74215209216094	-0.38797933218581	-0.98407330922101
H	-4.07944809642055	-0.17304164982090	-1.51358165000809
C	-2.91680816949812	-0.63970140123251	1.00089203655549
H	-4.99670866932480	-1.08488602560913	1.26031271246452
H	-4.17742419284821	-2.07409585266870	0.06268512242947
C	-3.40990034476282	1.75251854605564	0.41688900241070
H	-5.02971586582032	2.03607346549812	-0.93403044262373
H	-3.60542689685445	0.85978596973181	2.32149037747180
H	-2.04803570727997	1.05770142104911	1.82191405801124
C	-4.03033664979921	2.75378244633774	3.83099250784943
Co	-4.54799464845448	1.10767988088610	4.17411256179833
C	-6.02885403429650	0.54946635611800	3.40094311949042
C	-3.33199746701938	-0.10180779315962	4.56793061546351
O	-3.64848753929073	3.79134515161941	3.47438802322860
O	-6.94176607867019	0.17288433922116	2.79239728232826
O	-2.49942720682180	-0.90330239726270	4.68570896484309

TS1

C	-3.46035574716639	-0.72574220459241	-0.34364937919845
C	-2.11010509523383	-0.23796752252878	-0.86296012190912
H	-4.22813659940541	0.03339920472848	-0.52126325857745
C	-2.96700373095591	0.14056834273163	1.90036026438203
H	-3.77759141932511	-1.63424832863248	-0.85521627569185
N	-3.45135817486110	-0.99684291102699	1.10258747039619
H	-1.33364731589225	1.53608152492029	2.01810336166032
H	-2.89708245666176	-1.82505765829004	1.29976743539053
C	-1.61977310461596	0.96870761365426	-0.06001631788353
H	-1.38079747650095	-1.05158117242768	-0.78634227854384
H	-2.19949936256627	0.01168316877111	-1.92316749592669
C	-1.60471860474356	0.65118828060198	1.43682372629184
H	-0.62514858828018	1.27023500058854	-0.39498872304258
H	-2.28814259674206	1.81754237216100	-0.24106929945966
H	-0.84652377892905	-0.11173981936898	1.64384561350621
H	-2.94138513102865	-0.16729545478802	2.94553918856639
H	-3.71616301024921	0.93317579772128	1.81254805927359
Co	-6.55280489192257	-1.32372874396214	1.85464164803051
C	-5.78290504602768	-1.09106492002122	3.41878201704840
C	-8.32495333952085	-1.24625345249309	2.15097358734671
C	-6.33308469036264	-2.38496210574535	0.47338595347687
H	-4.90643983553903	-1.32996939664689	1.54181791787999
O	-5.25561893453246	-1.05777834621516	4.44216799444526
O	-9.45017821585702	-1.24998902513323	2.36206124023348
O	-6.15991629528450	-3.16643214500793	-0.35484253643594

IM2

C	-2.05709064399651	-1.24613607965898	-0.29808381507317
C	-2.15740270476536	0.20542146748708	-0.74002252116684
H	-3.04694315705710	-1.71643150503908	-0.37190649716670
C	-2.51071867205554	-0.64295748724093	2.02149215052123
H	-1.36871547089137	-1.80553608572144	-0.92953209386935
N	-1.57752812382633	-1.34361682401299	1.10312136494115
H	-3.38846891890795	1.28345821846560	2.32222066948359
H	-1.59851043711056	-2.32439230254789	1.35534719092916

C	-3.07417350312386	0.99791384179054	0.19481587838574
H	-1.15877183914946	0.64811398206962	-0.75274169976370
H	-2.53494009374306	0.23751106155854	-1.76430068496030
C	-2.65277057133366	0.82721840238019	1.65618951285099
H	-3.07313505455298	2.05525066361950	-0.07796692660054
H	-4.10226064965928	0.63945602296706	0.07415419950480
H	-1.70659670623619	1.34021112361757	1.83485490436852
H	-2.13547830680604	-0.75982378915795	3.03712879304808
H	-3.49539409683748	-1.12566746753730	1.96506480115807
Co	0.51900211234939	-1.01837008622805	1.40859012825833
C	0.82807065149366	-1.14033229187299	3.14888518115953
C	0.72610395462392	0.76079289026987	1.24525686489495
C	1.46359823436635	-1.46662689904951	-0.01655176588873
H	0.61558774527342	-2.50152535265593	1.57430734009842
O	1.04828967157942	-1.28984293708147	4.26468722316487
O	0.95040950387618	1.87786307944833	1.15266412602813
O	2.10692107649044	-1.81617264586935	-0.90034832430625

IM3

Co	-3.63452283591629	1.95486616735419	-0.55654883385727
C	-2.15401326341961	1.21747154984418	0.10513272402046
O	-5.51744012352603	0.03436630746781	0.65601017997175
O	-4.38901506265377	1.66860943801607	-3.36314561858213
O	-1.16731926060268	0.76883986533740	0.46810628980569
C	-4.14121556978056	1.77592688731375	-2.25205878530333
C	-4.82053063737037	0.81757152313841	0.21054775532387
H	-2.59317046785192	2.75270953542141	-1.25726149402761
C	-3.77537870338178	3.65565611522618	0.85667472538973
C	-4.63764499493846	3.98289868037084	-0.15729794773819
C	-6.15154763214963	4.09276497329201	-0.05278384192263
C	-6.84624525592161	3.47666624846345	-1.27507948048250
C	-6.44631461865012	5.61093152326600	-0.04519081862400
C	-6.71982021173152	3.48473279849249	1.23200094210347
H	-2.75172440816786	3.99974071756729	0.84009758528800
H	-4.14437465357816	3.30476020819019	1.81051288853720
H	-4.22941877781035	4.52764060327699	-1.00180178541324
H	-6.75102579208428	2.39114551425177	-1.27934272921880
H	-6.41705788045384	3.85778380175035	-2.20376680153111
H	-7.90970966872845	3.72491419689015	-1.26528446995270
H	-6.08402967781648	6.08509775551052	-0.95959886530358
H	-7.52301394339482	5.77991595902830	0.02254416055522
H	-5.96739710233713	6.09787530311997	0.80633665959478
H	-7.80256148241761	3.62156834065682	1.25132003199659
H	-6.30797950151780	3.96955091765733	2.11849301271293
H	-6.52424047379834	2.41612206909576	1.30655151665741

TS3

C	0.55685060003342	0.53305193221301	-2.95562023561400
Co	-0.17277457398485	-0.11460128779228	-0.09952065753650
C	4.37827256781311	1.60414600604780	0.93185092378429
H	4.30527017657553	2.42335423427129	0.21194896895234
O	0.59498278087791	1.52273435937815	-3.49160696711107
C	-0.91840955307329	1.49443965568419	-0.10849094034114
H	3.74899527536676	-1.80831943681872	0.88071596575250
C	4.05119673151942	-0.85517713393875	1.32174647806463
O	-1.41593594987497	2.51682828787222	0.00693207872323
C	0.18990409983942	-1.45374379855411	1.03386256309233
H	5.07854685053059	-0.95965547653942	1.67821879845018
H	3.41820707036061	-0.66979153064783	2.19080652769271
O	0.34728973364933	-2.24036271307983	1.84569110227844
C	-1.46831657195860	-0.96691328473329	-1.02705228209010
H	3.75119538408337	1.85201004654532	1.79002319370789
H	5.41341805574914	1.55045711555625	1.27753607535288
O	-2.28708393356750	-1.50472620320689	-1.60773246045510
C	1.43427171403592	0.79146874440560	0.73862115228923
H	5.92248664380462	-0.14197243747911	-0.54265687215633
H	4.60054030828214	-0.97574442892836	-1.36900354633114
H	4.85768859863287	0.75439357077160	-1.63294288553506
C	2.51128379815393	0.38466051200884	-0.26668082490561
H	1.33566856791486	1.87203773846305	0.80814825507117
H	1.64152041595117	0.42320872510938	1.74037505108661
C	3.95612686303397	0.27704209473295	0.29228740463904
H	2.28070627229890	-0.58987725027310	-0.71857712360988
H	2.52182097052409	1.10161803898361	-1.09120719352259
C	4.88927410342732	-0.04016408005163	-0.88348154972898

IM4

C	0.31170588613758	0.92391426208511	-2.04425039056444
Co	-0.36739527474500	0.07378867795148	-0.59992107294398
C	4.28741862039119	1.80219760817523	0.46662476274807
H	4.23548339909460	2.27983129804291	-0.51531263778271
O	0.75947606878599	1.48724410537362	-2.92864720824362
C	-1.06634443028317	1.10708817662740	0.71679667581274
H	3.87886204624507	-1.41788190097494	1.69145821193402
C	4.07400606874393	-0.34402130779914	1.74986427395260
O	-1.44648664833157	1.76472295787604	1.56596485466323
C	0.08065563859731	-1.62288491908147	-0.16424982449998
H	5.05095330468983	-0.20447361624738	2.21885701579949
H	3.32516446419823	0.10041610033091	2.40743273684297
O	0.38781893751880	-2.67491368414258	0.14998817762095
C	-1.94873706276582	-0.35692335525341	-1.38003166154796
H	3.54866358963578	2.27819600114099	1.11352378737345
H	5.27555295695584	2.00436457878371	0.88687523020023
O	-2.93591221102110	-0.63095584915357	-1.87187962640649
C	1.47298041884054	0.56424134100760	0.30647765018936
H	6.14875808191631	-0.15219760822521	-0.03163828018991
H	5.03727702850674	-1.40703529846576	-0.59393135970535
H	5.19043575293588	0.10659925781998	-1.49523690861525
C	2.70953968332639	-0.00883551873343	-0.36471472937131
H	1.49546968706488	1.65096359574007	0.31730541187781
H	1.37479544404594	0.21422141769351	1.33095764935495
C	4.05232317688079	0.29087266889719	0.35371575796756
H	2.62500423167314	-1.09473281304820	-0.45423751323212
H	2.79194606826654	0.37404777785384	-1.38516296296267
C	5.17358281928242	-0.32744926458494	-0.49244014513436

TS4

C	-0.30056125577997	1.08049390409479	-2.27509987035436
Co	-0.22091300455412	-0.14810587354577	-0.94344748003428
C	4.27895653214880	1.80654356766641	0.12171857697231
H	4.25761785105964	1.92998277955931	-0.96396532781203
O	-0.39552840500909	1.87269470785440	-3.08922518234613
C	-0.43427004559462	0.97928419450498	0.37712549392538
H	3.75019190348226	-0.81713983801709	2.33368913510701
C	4.00905209093732	0.20185734760168	2.03458312726226
O	-0.90757959876184	1.71388109419162	1.14626378451072
C	0.64720123882682	-1.53730838462980	-1.77444193964171
H	5.01280294453133	0.41703829616614	2.40763356576419
H	3.31963518416510	0.88698570570505	2.53082711616911
O	1.18048528690776	-2.40851939537256	-2.27324587708322
C	-1.69110104124636	-1.08686270023841	-0.55747585103137
H	3.56567665859452	2.50890386831828	0.55679074566758
H	5.27340462399657	2.08968531366219	0.47335120744845
O	-2.65645250623734	-1.65402366681728	-0.33451725358038
C	1.40179733510158	0.64249369381008	0.57800294897295
H	6.02463181652268	-0.29052678698066	0.21773704427284
H	4.84560338573053	-1.60727880462102	0.15844409696643
H	5.00085909991258	-0.49689532269981	-1.20889291369719
C	2.59248496984224	-0.07746551141617	-0.06770099887595
H	1.58131782236141	1.71499743835621	0.57165459424125
H	1.27724164198440	0.31402464057526	1.60633571809330
C	3.96712288568827	0.35590485223533	0.51022134656850
H	2.49196698643767	-1.15416415282647	0.07681443239856
H	2.62908675909522	0.10907251376415	-1.14061980608388
C	5.02226658644127	-0.56514879121153	-0.11837255866315

IM5

Co	-0.90037638409105	0.21063744253103	0.66153912949488
C	-2.59289077768732	0.69782033189382	1.16731174760712
H	4.52184968051493	0.01088657885840	-2.39658131310632
C	4.61128347582014	-0.88447228269487	-1.77629542287061
O	-3.63829685345195	1.01318253389948	1.48455269689256
C	-0.54409589633086	-1.00594910548539	1.91388193384997
C	4.73800053042974	-1.79776285917086	0.55737238945373
H	4.73838352737075	-1.56207881446226	1.62441713564599
O	-0.32158573600404	-1.87585882863722	2.62332196611436
C	-1.20302341161883	-0.17166129607731	-1.05350853148318
H	5.52073305774829	-1.41103839219122	-2.07405502384595
H	3.76600324147097	-1.53829246347261	-2.00046478138044
O	-1.40955933231544	-0.54173834149310	-2.11649883627997

C	0.92948481648521	0.48201963222209	0.36356187806477
H	5.65277648691685	-2.35134174745853	0.33488428346506
H	6.82630001407321	-0.23858503257217	-0.28953287579401
H	5.91401576456333	1.24124656864930	-0.61305456897932
C	2.09289750839943	-0.34123161323356	-0.07436500917039
O	0.92188303569808	1.65097473456499	0.67337479400864
C	3.44793549824782	0.33628186707322	0.11337742555011
H	5.99776822518431	0.59601728418958	1.03089600405451
H	2.02245474338211	-1.28979400656171	0.46224936644388
H	1.89424563204330	-0.58942607463905	-1.12261044791633
C	4.66843277077885	-0.51791551339027	-0.28746708831755
H	3.46131123018822	1.26501243721978	-0.46222540135525
H	3.55237257007183	0.62133785840473	1.16373009469755
C	5.92644346004950	0.32083492027017	-0.02423838417128
H	3.89655546232038	-2.46335048281400	0.35721122233072

A-IM6

Co	0.00164774995950	-0.09673261691984	-1.84275018423765
C	-1.80674341633511	-0.16689233544570	-1.85780763668849
H	4.36134973028410	-2.15015428507987	-1.51985340511316
C	3.96270335203243	-2.28517508982650	-0.51323841214952
O	-2.94406456117135	-0.17637652802761	-1.76478385981413
C	0.11751947767613	-1.38815717388349	-0.62056671986879
C	3.84119154272993	-1.39058557119955	1.82204355302129
H	4.16037753975302	-0.60607903820781	2.51292179897670
O	0.17696678826255	-2.19348478992709	0.19432047201858
C	0.02704577354097	1.67694711334402	-1.71522035790254
H	4.29000100609224	-3.25898494067449	-0.14085426338936
H	2.87487688733449	-2.31309717718848	-0.58458510226643
O	0.03175438625160	2.81886834171320	-1.60222950776681
C	2.00798304673493	0.06821747900483	-1.66145981545345
H	4.16560266920370	-2.35083774192046	2.22978287597400
H	6.29157594636013	-2.20862559708741	0.93176182780516
H	6.43941271700159	-1.11088736727364	-0.44683944235024
C	2.59081666365974	0.51386443170278	-0.33098856135157
O	2.72573840409832	-0.12161255229840	-2.61997914469950
C	4.07800037355692	0.22805702740335	-0.11072954263620
H	6.35654609962338	-0.46174574950248	1.19582154476048
H	2.42279169307849	1.59765434972283	-0.32546380552404
H	1.96958493179099	0.13353703840149	0.47807355180493
C	4.44394098559788	-1.17281827410995	0.42698189833739
H	4.60778470992250	0.39663624752632	-1.05081087239913
H	4.46157932782115	0.96424574883494	0.60134493279862
C	5.97413754353460	-1.24216901023889	0.53314428509081
H	2.75017764819707	-1.39821881927567	1.79746908214204
N	0.36008199766321	-0.79349147173556	-3.82638334549595
H	0.73382843705556	-2.53039087338703	-4.96777587899605
H	0.60884422233435	-2.78272067813296	-3.22261090090716
C	-1.64620687375127	-0.28869555369245	-5.21524086308398
C	-0.17133654767520	-0.00730293209988	-4.96546086897729
H	-1.45259371358036	-2.11073069345695	-6.34496360145888
H	0.39949797265437	-0.26491201478522	-5.86769574592170
C	0.17677571964821	-2.24483936813037	-4.06520568503497
H	-0.00288026377558	1.04794282197396	-4.75406073054488
H	1.36462228295717	-0.62035547649313	-3.78229032304618
C	-1.90184798394190	-1.78661127805709	-5.40008646481348
C	-1.29016148033229	-2.59909603907678	-4.25622150220458
H	-2.97370742864464	-1.98154644822303	-5.47528690807184
H	-2.23935187958966	0.09251974842234	-4.38255771797496
H	-1.96288856896225	0.26343130127255	-6.10303435942895
H	-1.83541350299551	-2.41407731791463	-3.32771579626559
H	-1.37222901306959	-3.66859986395736	-4.46242882135770

A-TS6

Co	-0.50748349081694	-4.31003605176024	-1.80894404193413
C	-2.00560985884717	-5.07543985480403	-2.44003611449981
H	3.06829371865840	-1.92692701321353	2.39808816938765
C	2.54886973517328	-1.03931957206080	2.02891881620853
O	-2.95472915112001	-5.61373169038098	-2.79331166229721
C	0.24689353368221	-5.69015034382498	-1.02497161508428
C	2.35440810249928	0.63673282116914	0.17547557850288
H	2.74767652336580	0.98186551138971	-0.78426847963951
O	0.64279920739629	-6.57070502304449	-0.39867649018235
C	-1.15341753318040	-2.87886594550694	-0.99098635606146
H	2.65168835406686	-0.25127458999871	2.77863667036419

H	1.48728018056016	-1.27765308002467	1.94646745041170
O	-1.66076827504934	-2.07122368252073	-0.34326283656053
C	1.75851532204520	-3.50161705343812	-1.57962727562473
H	2.43115387970358	1.46455363167105	0.88421039971040
H	4.67851097990822	0.64018662262662	1.62696980403624
H	5.18857501202497	-1.01672052296421	1.27893053883096
C	1.72464919351547	-2.25936008264499	-0.69726180404861
O	2.62707060999664	-4.34757528925833	-1.45115394605310
C	3.11866593811452	-1.72903106456320	-0.34340192098654
H	5.05672260898403	0.16149761324433	-0.03283905982601
H	1.09953461433246	-1.49479592499272	-1.14767955985282
H	1.22440185855491	-2.57938384617146	0.21528360472719
C	3.13747133468637	-0.57982160236987	0.68816943446941
H	3.70915021675527	-2.55981230360056	0.04779684899504
H	3.63166821409358	-1.38418187601150	-1.24315283509652
C	4.60260704941982	-0.17454892838292	0.90290437171855
H	1.29456628971588	0.41032405509490	0.04666176647598
N	1.71466868139820	-3.11479562053030	-3.33474803161511
H	0.52153703948411	-3.08125386950414	-5.04843775504114
H	-0.35629298615144	-3.44770790114835	-3.57794233288031
C	2.46731722628709	-0.74751283066019	-3.73966414939918
C	2.81376293472525	-2.22858096650796	-3.80908205550881
H	1.26144491213191	-0.63667295800741	-5.52708421141474
H	3.01843700677459	-2.50596419796923	-4.84861320132872
C	0.42663773810265	-2.79705747419846	-3.99517644741559
H	3.69947201016793	-2.47254000497084	-3.22501049409522
H	1.98943501485452	-4.06828340646983	-3.66407866849343
C	1.15001089084396	-0.44762124455440	-4.45412985223253
C	0.03683557817302	-1.33323331034998	-3.89542330078881
H	0.88964950204374	0.60605649416586	-4.33907654261557
H	2.39619369426647	-0.42606634407687	-2.70109241661242
H	3.28902474918497	-0.18519585352074	-4.18801281527934
H	-0.16572067777578	-1.06789139162873	-2.85682896189008
H	-0.89395079152016	-1.18749693759315	-4.44659602474087
N	2.78239638636763	-5.59833938267937	-4.38212039426535
H	-0.11202155812886	-6.89286356492696	-6.59334763875916
H	1.36316192785711	-7.83110190575397	-6.41974507897973
C	0.71767271512926	-6.89904346003750	-4.58851689096316
C	3.03101842204967	-5.64539711369527	-5.82858578841355
H	1.89944304173193	-5.77699841591311	-7.65529178539923
H	1.97147754537041	-6.72183768484936	-2.82928483781643
C	2.07739078328003	-6.79866141101420	-3.90956785835362
H	2.65978363868722	-7.70753408011130	-4.12615404881048
H	3.66067593969856	-5.51431702244527	-3.88230967587275
C	1.70623441389922	-5.71172856825735	-6.58190940875342
H	3.59252032132373	-4.75340615646133	-6.11334415362836
H	3.64192301621844	-6.52120662542438	-6.09741352507698
C	0.86837363934662	-6.90347371638677	-6.11127050302794
H	1.15325099468117	-4.78335003911037	-6.40919172831439
H	0.20454775665051	-7.80103564427479	-4.24785806713102
H	0.10441432460660	-6.04844030479116	-4.27735681117306

B-TS5

Co	0.24190505728940	-0.98336502153497	-3.19751173534415
C	0.66766277405005	-1.39472758204192	-4.91715459353306
H	3.56843679426457	-3.23493765196233	1.49670854125082
C	3.61618202556987	-2.16065807695596	1.69233148427023
O	0.95946247356820	-1.66794858633469	-5.98655921190247
C	-1.47696750722144	-0.41216266236659	-3.35439809980198
C	3.90354934872688	0.12595455622739	0.69954989786438
H	4.06790405946382	0.70431460049636	-0.21252620960607
O	-2.45597855703782	0.14587204231382	-3.57189295893100
C	1.35562034501571	0.34717656154998	-2.94710552433684
H	4.40946853098456	-1.98597062147846	2.42269878969191
H	2.67337681798866	-1.85759423243011	2.15171659952120
O	2.04390571436979	1.24378720293668	-2.73732126561403
C	0.46977166550491	-1.66319093793787	-1.43598872750531
H	4.70254855626545	0.36900222275827	1.40362226389778
H	6.05656434279990	-1.55998424239279	0.58262048617882
H	5.30680894573433	-2.85686517755155	-0.35736412458192
C	1.39778785074573	-1.42826409483989	-0.28338579569192
O	0.30144303451704	-2.73071162556873	-2.02177400175511
C	2.85062162137778	-1.71510503869114	-0.68354226769367
H	5.50058703664231	-1.24884734532801	-1.06674111199401
H	1.27484427616011	-0.40394770309831	0.05655691149000

H	1.10089137658373	-2.09664168706409	0.52292397389210
C	3.89550260722682	-1.37934875782537	0.40153378491823
H	2.93490652048944	-2.77362857530718	-0.94196994625169
H	3.10080145456666	-1.15282508427595	-1.58507810915183
C	5.27184136468986	-1.78597359486730	-0.14301856823509
H	2.96304529310953	0.45673224762082	1.14389653427141
H	-1.44090296437021	-0.31704404586393	3.37026998082026
H	-1.43813552319015	0.67291327676362	-0.81757257136135
H	-1.74539991231959	1.41023642899931	1.55318439998001
C	-1.80643564610999	-2.37721445457471	0.60416721580946
H	-2.91473218753479	-0.57997265562516	2.44798141639518
H	-1.74205489432189	-2.73943666002406	2.71004677451866
H	-1.87608300601855	-1.62118859701769	-1.27719838913149
H	-2.93918036291569	0.08959673371061	-0.09439534816865
C	-1.84474763543053	0.00709578788440	-0.05614059607460
H	-0.27171666015829	0.48087538774094	1.33840676413567
H	-0.26147595437297	-2.04486377130182	2.07809961362408
C	-1.36231380729094	0.41220989242676	1.33071183979007
C	-1.82104452863366	-0.59416066554736	2.38478710127926
C	-1.35049605721238	-1.99859680475503	2.00963948982613
N	-1.43534614285451	-1.36465613653701	-0.40234856889779
H	-2.89694595482626	-2.50669491935576	0.60838524278834
H	-1.36613358588837	-3.32602493096993	0.29133361935022

B-IM6

Co	0.22044307335012	-0.99340400930287	-3.02260498952414
C	0.86759956582168	-1.59923918196880	-4.59272560780804
H	3.50582630012437	-3.04952409784730	1.62718558353909
C	3.63359063460898	-1.96899853237266	1.73178778553342
O	1.29892933596695	-1.97860485020382	-5.58363821767244
C	-1.44652538127285	-0.44926686935100	-3.43133803015838
C	3.97008867640778	0.21389714330700	0.54285038397549
H	4.07914535433871	0.71300089560180	-0.42259630217039
O	-2.36677349449438	0.10743649571992	-3.85069654069150
C	1.22586766279865	0.42519423191564	-2.80048924458994
H	4.49977014793391	-1.79285201636355	2.37381212602652
H	2.75689113710062	-1.57225483697367	2.24754054564966
O	1.84074174991568	1.39074019125000	-2.66288100804108
C	0.20301237056956	-1.55303089303246	-1.12193311313426
H	4.84831964133837	0.45083108882600	1.14795513758471
H	5.99002522554152	-1.63366972046965	0.37768772642835
H	5.07182778789815	-2.92608630547075	-0.40685843175594
C	1.29520394103900	-1.20759814911409	-0.12650246899916
O	0.23397750035453	-2.62969377314485	-1.80878849261338
C	2.67888228402644	-1.64431046902899	-0.59761166945867
H	5.31321774481581	-1.38441874327484	-1.23687905450280
H	1.27406690458967	-0.13792166037583	0.07447072581995
H	1.06876719953899	-1.71227455016074	0.81426746860233
C	3.83721689110009	-1.30474596037564	0.36317405179012
H	2.66260849983760	-2.72374562104122	-0.76359008429836
H	2.88977527381694	-1.18940078796915	-1.56668993915227
C	5.13040552193519	-1.84446903575316	-0.26289091813771
H	3.10020764148456	0.64063257067612	1.04581682348138
H	-1.62952678009044	-0.64263285272105	3.37551503770772
H	-1.06989473584807	0.72446152716733	-0.67864607282983
H	-1.66429968274899	1.24998545371281	1.70751098334990
C	-1.71218922764749	-2.44867932405072	0.41990537619864
H	-2.98309204370298	-0.76939296071206	2.26014163337638
H	-1.90650630923509	-2.98190001598886	2.45948950088319
H	-1.78938160728897	-1.48118743320867	-1.36813347086625
H	-2.66682860988432	0.15421204665792	-0.18918283343772
C	-1.59512002554536	0.03285646029403	-0.02368293692056
H	-0.19627513001651	0.30049860923183	1.60155767155944
H	-0.34084582864183	-2.29710423436362	2.08449844485619
C	-1.27465815916226	0.26461742178107	1.44512515560358
C	-1.89129041757543	-0.81602914204949	2.33067344035932
C	-1.40881001521904	-2.19541140547987	1.88831000427530
N	-1.25586434108266	-1.34829160253427	-0.50994291472943
H	-2.79167119663992	-2.52428342064338	0.27766876033250
H	-1.25458869105855	-3.36250962385259	0.04752749378436

B-IM7

Co	2.66181296257828	-1.86197229451632	-0.43828243814551
C	3.18903345419377	-2.47468527849942	-2.04011786137052
H	7.18234118815822	-3.53178555647948	3.10751796503617

C	7.20465291992413	-2.45075944554195	3.26858038591598
O	3.53052023500120	-2.84067019411762	-3.07541918045565
C	0.94197297904627	-1.44825771105090	-0.59970336923281
C	6.94269137028256	-0.18514570013816	2.22638501509284
H	6.73153497189286	0.37430556169784	1.31207443897896
O	-0.06908662861599	-0.93815111602817	-0.85955881891463
C	3.46640966399004	-0.31070881617167	-0.31881111338075
H	8.18614167076672	-2.19050333494312	3.67258571355836
H	6.46103811615837	-2.20684985684190	4.02963928813312
O	3.93204830694982	0.74401973045074	-0.24686223708867
C	3.06724695482211	-2.41002761043675	1.52360999349311
H	7.91559024294403	0.13841145372207	2.60475776340544
H	9.04976950534647	-1.72563282905883	1.38278686428677
H	8.11410720822998	-3.08928959617723	0.75409002970636
C	4.36716922632583	-1.88544452448121	2.13173545885346
O	3.11904044840679	-3.44429827417826	0.75653347714424
C	5.62163175665466	-2.14417077915985	1.30587623075954
H	7.93720860858308	-1.48755322574039	0.02826319251076
H	4.25536654509772	-0.82629072620547	2.35916331227247
H	4.48103235470578	-2.39147374895461	3.09340418574656
C	6.94756806825867	-1.69621666000592	1.95668588518715
H	5.68178552477116	-3.21424958217385	1.09333684370196
H	5.53061718877952	-1.64892280301179	0.33860197184286
C	8.08033472983396	-2.01887595758046	0.97215444460100
H	6.19169593017885	0.08985695162215	2.96912917822636
H	2.06838624333027	-0.69050419864708	6.01785507200879
H	1.32863949356254	-0.48277946034076	1.77625083691736
H	1.12653271943605	0.56383693163828	4.03492707659793
C	1.99882770824987	-3.21161020280447	3.62989717922408
H	0.63805545758951	-1.47050612416658	5.35581182141017
H	2.36484791545344	-3.19101163662328	5.73949028623207
H	0.88761110368393	-3.48911334874937	1.49048187598347
H	0.16381221270664	-1.28924967975998	2.81922992852701
C	1.23274618578661	-1.08687374470410	2.67368180043236
H	2.76645767174339	0.00949115897715	3.73606476335103
H	3.53904020375269	-2.25780799679583	4.82943464521264
C	1.74098439338361	-0.33017494107831	3.90093083436112
C	1.68340649422625	-1.21780495579341	5.14226359988336
C	2.47745729915914	-2.49977459310488	4.89949690341621
N	1.88266400354049	-2.38007326086687	2.40875732762316
H	0.99856573858651	-3.60976380434948	3.83874955820745
H	2.63705947496628	-4.06424679738529	3.39510741793119
H	-1.23336999726471	-2.73304882889141	0.77108621672823
H	1.78801100817926	-5.59388017622889	0.86613867320772
H	0.75414662619427	-3.70889857860411	-0.09693933296787
C	-1.39274816090025	-5.87315463208437	2.25214436403896
H	-1.56337970411733	-4.27001025673624	-0.03727471749208
H	-2.76460110698584	-4.19233700907918	2.12464970813103
H	0.61834469240826	-5.71809749176245	3.02514140042951
H	0.30225025495401	-6.02491051256390	-0.00003834645699
C	0.70287926180103	-5.53334741930333	0.88805432236563
H	0.31822679381538	-7.20781773391490	2.14867496421810
H	-1.27055407585935	-3.83955177479880	2.97869402926678
N	0.36021767925621	-4.08494036185095	0.77357653663181
C	-1.10456988753731	-3.81056328315562	0.83968882001475
C	-1.68933262835953	-4.37805585402966	2.12312091541450
C	0.11108128880188	-6.13626016724937	2.15262577722231
H	-1.91171153927095	-6.41469490190896	1.45456162031286
H	-1.77840216832188	-6.25351789928818	3.19949160045396

PC

Co	3.42391705795245	-3.76314291086965	-0.67974613454917
C	2.94808061269290	-5.36451460514100	-1.19827119516701
H	5.76792773894386	0.74881370310564	3.64884111132949
C	5.66272410792040	0.93103618547064	2.57583316843659
O	2.69163069473594	-6.39194822588899	-1.69076947324261
C	2.71010331873431	-2.44940703726085	-1.57863872074798
C	5.92102258641961	-0.01649329363383	0.26616678180233
H	6.17901695369212	-0.89286249681678	-0.32959062636152
O	2.30202501030040	-1.62449891296480	-2.29977955002392
C	5.17682115390909	-3.64494985387106	-0.77307631056706
H	6.29075077687125	1.78685598075607	2.31816132108374
H	4.62692689319043	1.21516575571448	2.37861407100650
O	6.31950010935362	-3.58554827368698	-0.97644009628928
C	3.02488984595894	-2.68870945968182	2.31649186191024

H	6.57799938500143	0.80398633254458	-0.03181661926410
H	8.19066133333928	0.23025916899443	1.80343602788858
H	7.70115017464781	-0.84528108979985	3.11906401433517
C	3.75222207123527	-1.45371541654751	1.85615016345643
O	2.81327097279527	-3.62088502333092	1.51009878329253
C	5.25057529782076	-1.53271370088459	2.18659423970728
H	7.88736819000439	-1.48163300247490	1.47874987895059
H	3.62049787010401	-1.43038183632637	0.77603099024826
H	3.30208409147582	-0.55563535400028	2.27726841954456
C	6.08066375109503	-0.30269427856757	1.76490156226135
H	5.38522301385674	-1.68278762045656	3.26155893400730
H	5.65588750795021	-2.41784593164153	1.69246183312457
C	7.55336926110356	-0.61924520972994	2.05968529640901
H	4.89880726859298	0.27130197918133	0.01500798234422
H	3.60986004128380	-4.06641184471849	7.12767652131210
H	3.34528835876541	-0.92017958159091	4.21119884365008
H	3.74162282123091	-1.60017142392005	6.57225678549961
C	1.95192726522778	-4.00257722970744	4.08152771991011
H	2.03164090280852	-3.36018476253771	6.81009635318724
H	2.19493822736162	-5.50248546501428	5.59655544106058
H	1.00408707286670	-4.14779831780633	1.08465619663120
H	1.86161487991910	-1.44536176228694	4.99746341019655
C	2.83982181147468	-1.77965532846841	4.63437075314021
H	4.65580775736494	-2.59382544532363	5.44040012734365
H	3.69866965191165	-4.99480836891634	4.83301855243311
C	3.64740648309223	-2.36096568929107	5.79418214094167
C	2.98838059641710	-3.62485023259293	6.34616702573350
C	2.74329481318845	-4.63401375925326	5.22482231228690
N	2.61405912992173	-2.78810155037943	3.59611776874020
H	0.94983923309361	-3.72632462345645	4.42808239823262
H	1.85273509525340	-4.69134614970941	3.24954560144656
H	0.49847572590498	-1.93220122499545	0.54323475111645
H	0.11898472457535	-6.03909080172482	-0.05661039597290
H	1.17615169353514	-3.93973813507003	-0.49142996016028
C	-2.26429226021526	-3.90304268590956	1.31557194166613
H	-0.74902501202877	-2.48136015518385	-0.57979885459509
H	-1.81212156959458	-1.79052310783075	1.56057961022467
H	-1.00631407050375	-5.49266389630267	2.07129142133556
H	-0.97592503434100	-4.97373952871632	-0.94739733441516
C	-0.49584345449568	-5.14413643770857	0.01674746561695
H	-2.21352023651504	-6.03942489509379	0.91850185516543
H	-0.75023086682962	-2.86421433820006	2.46040177578939
N	0.42449692520099	-3.99598465388858	0.24904628114474
C	-0.27104948912338	-2.68385305461414	0.37885460481985
C	-1.28348218613036	-2.74382766550122	1.51162728707565
C	-1.51814226998045	-5.22832587796638	1.14031818194183
H	-2.87588450943522	-3.71158382470134	0.42825529505263
H	-2.94553114169083	-3.96614921381290	2.16566450622369

C-TS6

Co	3.40628890123922	-2.26133729650552	-0.66955193407702
C	3.60705478215609	-3.17488792259360	-2.23293417949367
H	7.06115844709448	-2.34211688082888	4.47374464462558
C	7.03047222209759	-1.29693571611885	4.15509348631601
O	3.72749300143082	-3.71603435404037	-3.22502888826603
C	2.06115513503025	-1.13864062277624	-0.73076559040131
C	7.09167320785573	0.28899174149142	2.20967640856261
H	7.20760107056454	0.39458820108842	1.12889028107668
O	1.15406604871806	-0.44515196738135	-0.82820472795681
C	4.81372810257185	-1.07971093361050	-0.92781858345588
H	7.83008248333211	-0.76492940195420	4.67439663229248
H	6.08376707791177	-0.86554004172253	4.48772085508547
O	5.56144716463861	-0.27132478610129	-1.20302630668085
C	3.75982617893260	-2.60263066196571	1.42367124239724
H	7.86858956239220	0.88667126191155	2.69063128386795
H	9.38369679415601	-1.10637163771701	2.74928916673859
H	8.73432731505852	-2.73909491812280	2.55830514709540
C	4.72267982236105	-1.72267703710188	2.19228977138614
O	4.11128790733360	-3.60458123928821	0.75788261476729
C	6.18217652581341	-2.05610076019973	1.89130857330454
H	8.76756452404040	-1.63318467960417	1.17889505996796
H	4.49467574820513	-0.67329515432686	2.01032800922829
H	4.53937165231884	-1.89167292564242	3.25159669818568
C	7.21211421761326	-1.18035240112938	2.63543362586141
H	6.35363452435415	-3.10187998127801	2.15689609454483

H	6.37523572809824	-1.98318778408809	0.82097861884038
C	8.60756398238951	-1.69630440040951	2.25770266547949
H	6.12827679551580	0.71827471938959	2.49217958812830
H	2.19298134402878	-2.04035101388422	5.98822983075765
H	1.89830462720764	-0.75166405673961	1.89189569628675
H	1.32006239781184	-0.36278979324805	4.30678213796205
C	2.33916179602031	-3.93397908985283	3.09910173503393
H	0.83737914184500	-2.65164228190843	5.05257151213527
H	2.58070081449528	-4.38909331711242	5.14635982075867
H	1.81870452735211	-3.13067367496520	1.30284739144165
H	0.60215030624082	-1.76910110169177	2.51836218295818
C	1.66608455737285	-1.54575454219298	2.59562410674687
H	3.01149403638036	-0.73448205431873	4.08110983092793
H	3.79323887308939	-3.27804173160933	4.55646159620195
C	2.01026171996800	-1.16033645910296	4.02643288445954
C	1.88483904854109	-2.34130221093288	4.98678814230902
C	2.73038048645575	-3.51869049127754	4.50503351036543
N	2.36598413970720	-2.79124599163370	2.09416011677130
H	1.30860445109477	-4.29167450949042	3.08939335206885
H	2.97961881917826	-4.71003749714885	2.68793484393037
H	-0.38189107336121	-3.46943104378190	1.14925457481025
H	1.44634299549634	-4.97989273876831	-2.27899029588505
H	2.01850723781350	-3.12509050851735	-0.59341021106949
C	-1.95802040003682	-4.66721573041291	-1.69318112543892
H	-0.63923074518611	-2.58489349947773	-0.35295262613792
H	-2.65576932588843	-3.87330847624044	0.20642541851398
H	-0.56898478391602	-6.31387921325548	-1.83778203474251
H	0.48721908020443	-3.51482287583879	-2.46883666690896
C	0.54008654604714	-4.47499934328851	-1.94801835359453
H	0.92869902365011	-5.03474220684710	-0.01685707491865
H	-1.72154643507001	-5.35833748185052	0.33889078538441
N	0.68926202829567	-4.17757107097074	-0.50847752961544
C	-0.53127659347560	-3.58504517314443	0.07374658499837
C	-1.79091489860669	-4.40567064374129	-0.19652952175297
C	-0.69547412556792	-5.31291330983618	-2.26351789887868
H	-2.14693467928929	-3.71925824239148	-2.20818561972750
H	-2.82622465160799	-5.30379007742206	-1.87217006288897
H	-0.77452672588932	-5.43434746113973	-3.34605600108697

D-TS6

Co	0.13868887573059	-0.95520592360251	-3.07112249827776
C	1.36446349124093	-1.79510875250520	-4.05223936464548
H	4.06888328728933	-2.37106885960058	1.97643489641647
C	4.17137190058987	-1.32627321084213	1.67209165835734
O	2.13707348879507	-2.33632523757005	-4.71567120967486
C	-1.52190713316729	-1.17271494635478	-3.61624790970555
C	4.16985128446522	0.29361536751098	-0.24746199696699
H	4.06663494388432	0.41658680483828	-1.32759490248284
O	-2.59338877570563	-1.21691274388361	-4.05784558351649
C	0.64038596670208	0.61406243935502	-2.49490962832243
H	5.13939056088052	-0.96627035384020	2.02746063043218
H	3.40003334017415	-0.74558851394544	2.18293557297379
O	0.98917667643910	1.69146446496660	-2.22822107373382
C	0.26927569162113	-1.79767667538745	-0.43326879565124
H	5.13774527228937	0.70267898614512	0.05029524772065
H	6.19694831692475	-1.54574584075788	-0.16965729375420
H	5.20604406188230	-3.00935183147934	-0.23837079171987
C	1.48996804716846	-1.19358697106618	0.18370752077581
O	0.18170195273009	-2.87642526042035	-0.94015898644493
C	2.76792845415038	-1.80977835352599	-0.38179573056048
H	5.19501522308631	-1.87043151688211	-1.59036828069191
H	1.48289707713299	-0.11504299089538	0.06017405300582
H	1.40866079776012	-1.38520590898309	1.25953222107727
C	4.07487807903359	-1.18662195585921	0.14727722389726
H	2.76974153934548	-2.88041772395951	-0.16830484877437
H	2.74223170415212	-1.70411179491612	-1.46834809897981
C	5.23799151481714	-1.94941866867521	-0.50180028355131
H	3.39800318095069	0.89686986598202	0.23442839274397
H	-2.47089756462556	-1.52148405510229	3.29142056645741
H	-0.26753785092291	0.80453428150066	0.39876294160041
H	-1.37834512786806	0.67392002370896	2.64045950377754
C	-2.25222315531707	-1.91772157942174	-0.14134881415256
H	-3.38011996460458	-0.63662188857905	2.07502210250958
H	-3.24431709175025	-3.00439023991641	1.39329590901575
H	-1.10723817627914	-0.64141790712910	-1.32489448398431

H	-2.01577959897988	0.73847772949824	0.26844061376334
C	-1.13486760198972	0.18071129249471	0.58827188403167
H	-0.34088563728025	-0.71377945610980	2.38173493146135
H	-1.49788470067729	-2.99508197137131	1.55613117002119
C	-1.26231116092989	-0.23373153410013	2.04574692942484
C	-2.44645799308362	-1.17821092504978	2.25628193110813
C	-2.36028957233518	-2.37104924444792	1.30395247513275
N	-1.06897842557649	-1.00578404412332	-0.32971859742489
H	-3.12185554193367	-1.33513169900690	-0.44524925302290
H	-2.12743326711164	-2.74380061974780	-0.83309043044026

Acyl-DMAP (PC, in the presence of $[Co(CO)_3]^-$)

H	3.82389982128902	1.16125667441924	-2.24655037852087
C	4.00968361276422	0.10007483723087	-2.06329581192491
C	4.68191331538884	-1.63245750660367	-0.37703231187703
H	4.97344322974066	-1.82454219863195	0.65888828525072
H	4.77934509032672	-0.23293975801121	-2.76339568656561
H	3.09537685676133	-0.44787178503618	-2.29711888013293
C	1.07367317239307	0.46751084842603	1.24435903363172
H	5.47528722030757	-2.01153337538272	-1.02516916417204
H	6.56244275363832	0.23824546800162	-1.07764949524873
H	5.67910274932899	1.67739240030846	-0.55340750144440
C	2.05841399698600	-0.18906215730611	0.31694982370412
O	1.36698417508048	1.29878579119952	2.07779153254793
C	3.44160283955433	0.44420483909100	0.38195139545524
H	6.15559097570234	0.45368829303977	0.63020637388234
H	2.10329507327312	-1.25267116852094	0.56999403360318
H	1.64189410718414	-0.14453921372261	-0.69203187219348
C	4.46719447972910	-0.13197210651307	-0.61651844602720
H	3.34441548590070	1.51761394481009	0.20149153060478
H	3.83621034195785	0.33836786631664	1.39519628459668
C	5.79553556107808	0.60315541279681	-0.39060141268402
H	3.78112878388727	-2.21129609578649	-0.59014815475327
N	-0.28077979362487	0.08299253437653	1.07161271151140
C	-0.65874588433166	-0.94171243182773	0.14755717738043
C	-1.24974081058180	0.82581857791963	1.67583729535201
C	-2.03588569136125	-1.31852518512119	0.07932096616471
C	-2.57171292129942	0.60389552860573	1.49029674527926
C	-3.02496149979189	-0.50190092534615	0.69509130910624
N	-4.32839290571391	-0.78855533826926	0.60545953705482
C	-5.33204064705175	0.01798823309401	1.29791971559382
H	-5.17002896109345	0.00877311668289	2.37700822553361
H	-6.31214628609118	-0.40131816513401	1.09408871623581
H	-5.31515371585701	1.05022218524614	0.94432520648520
C	-4.78354343932550	-1.93773109064621	-0.16912206439996
H	-5.83035712003787	-1.79953052302609	-0.42376643035109
H	-4.67533552945662	-2.86708265326748	0.39650857244555
H	-4.20940441393685	-2.01695415187908	-1.09032794901523
H	0.07213214206726	-1.73081363721478	0.09237239613099
H	-0.88329414494176	1.63275046786819	2.29076242992815
H	-3.27033365575363	1.25957085464839	1.98192981343105
H	-2.27402139168585	-2.33984856847892	-0.16720127718656
Co	-1.37403646546327	-0.39968507771124	-1.67875507628206
C	-2.83030558920104	-0.35099297441123	-2.70537180418640
C	-0.94426398649341	1.31957998859269	-1.54306039451147
C	-0.24337099924231	-1.43372977364020	-2.58374954507939
O	-3.76924175080541	-0.28568803947607	-3.36756211741814
O	0.51931445587966	-2.04674093158866	-3.19324200915396
O	-0.62030062627740	2.42082478517905	-1.44752051918077

Carboxylic acid product (4,4-dimethylpentanoic acid)

H	4.89656648133332	0.56098316730245	-1.40538228408869
C	3.84201387977953	0.79342178045233	-1.57056375884680
C	1.46725300442728	0.09332270236282	-1.16260106388213
H	0.80667457762631	-0.67379326281716	-0.75283563522056
H	3.67716525075481	0.86332787820772	-2.64760876020270
H	3.64043630366256	1.77716877562500	-1.14299531837723
C	3.38507369349605	0.42008960899609	2.84953499397484
H	1.24307047805413	0.19304196319519	-2.22652141380475
H	2.99030059436662	-1.54025535537601	-2.74137464602262
H	4.25025077263491	-1.92365259123469	-1.56304436442710
C	3.11218814994392	0.76211166297631	1.41444424806528
O	4.45164477133638	0.45713192026457	3.40184887598258
C	3.25929596617475	-0.49197899378280	0.53208884739939
H	2.57926539984242	-2.41803448592167	-1.26286580854653

H	2.11197586356518	1.18531594116056	1.32636954127609
H	3.83674795212988	1.51815172382908	1.11951576223645
C	2.93984769262757	-0.28826890079864	-0.96380281621038
H	4.28606982849955	-0.85430243803566	0.62505388970028
H	2.61222011884413	-1.28436947286492	0.91821924805618
C	3.20629840078650	-1.62270952870469	-1.67423146645436
H	1.22330376876142	1.04459902360793	-0.68618970688422
O	2.25931735599900	-0.00106862136201	3.51061498085383
N	-0.12650887636667	-0.81264951712916	2.07227968736338
C	-0.15571844419968	-2.10766937921096	1.68982497300788
C	-1.04083658218363	0.06621501860499	1.60823136088361
C	-1.10719490597080	-2.56815378880689	0.83190304543734
C	-2.02166123057915	-0.32531442569786	0.74897453241828
C	-2.09566911583601	-1.68080285848940	0.31468152390912
N	-3.03845894112978	-2.09718407357701	-0.53513421371401
C	-4.04141954968216	-1.16331360137036	-1.04821242620884
H	-4.62597301237506	-0.73304901227423	-0.23308334092144
H	-4.71367510450976	-1.70091616478962	-1.70807531170328
H	-3.57084352307993	-0.35696589148181	-1.61386038342993
C	-3.08241527967322	-3.49394130415615	-0.96937034109646
H	-3.91291032284938	-3.62041217892121	-1.65526660218580
H	-3.22931905667599	-4.16196582392198	-0.11875390514432
H	-2.16106461698246	-3.77089425728372	-1.48499755405021
H	0.61502773812264	-2.74420083524265	2.09820307191142
H	-0.94426656112390	1.08474727196736	1.95362987212350
H	-2.72777408745099	0.41258745624586	0.40633933196547
H	-1.08853018295182	-3.60929296110017	0.55591432269994
H	0.62509362316760	-0.48992679294086	2.68185377648356
H	2.51884204268315	-0.23638800250497	4.41821869847376

Ester product (Ethyl 4,4-dimethylpentanoate)

H	1.14671718038389	-1.55510250081112	1.17420756117491
C	1.84854361532336	-0.79473443239540	1.52450747146906
C	4.13868491595273	0.19793659295651	1.27096415536806
H	5.06163065617702	0.21098181404254	0.68657863768018
H	2.04248317524742	-0.98279677407824	2.58250044434931
H	1.36266323797454	0.17977485946033	1.44938089135239
C	2.08017939270311	0.82209551283203	-2.63243010490971
H	4.39916395736323	-0.02997012273006	2.30652092979508
H	3.96929784115842	-2.46732030577018	1.93316264344641
H	3.10307616491512	-3.01502534091471	0.49237213457746
C	2.36308419414172	0.76792740596198	-1.15716217332094
O	2.88585557280005	1.07722369386678	-3.48925918885377
C	2.89742053790201	-0.62440640689733	-0.77728286732611
H	4.72076012089749	-2.31711204393433	0.34074224496336
H	3.11070956464695	1.52956638743047	-0.94355023883960
H	1.45841920661228	1.00627564474220	-0.59910150336124
C	3.15825971649074	-0.84820426635624	0.72694401171437
H	2.19409322205797	-1.38806444096109	-1.12195943938801
H	3.82935929615561	-0.79071557794943	-1.32310672603095
C	3.77480305602808	-2.24557703397638	0.88178584517532
H	3.71154106565207	1.20217984283654	1.25276905169638
C	0.71666502797173	-0.96693767215699	-4.87282547898290
C	0.40881535544966	0.40795941249975	-4.32210238894009
H	0.19553537660089	-1.74115901810653	-4.30640136376723
H	1.78752005643785	-1.16742693421446	-4.84710773611118
H	0.38290084909703	-1.02396743381875	-5.91052137880257
O	0.78367961962870	0.50092348137421	-2.91283114943821
H	-0.65884427233216	0.61846830116126	-4.32723493985392
H	0.93432113110777	1.19018237389452	-4.86407266775731
H	-0.28381203026779	-0.39781005150939	-1.73024393364357
N	-0.78187716502707	-0.95506906914695	-1.03037722232723
C	-0.69015696176885	-2.30118867804841	-1.06914938282992
C	-1.47201293511215	-0.33818214863202	-0.04787435694232
C	-1.29199038587069	-3.07746574645098	-0.12598028056741
C	-2.09963730226176	-1.05052979312097	0.92823951498274
C	-2.03241560172071	-2.47399426167479	0.93196589396647
N	-2.62857990458432	-3.20414079933169	1.87906066482460
C	-3.37746730122972	-2.55540690363513	2.95570621135458
H	-2.72886329480787	-1.90391510176634	3.54437359479660
H	-3.78288797038773	-3.32052601920918	3.60880614207176
H	-4.20487684037205	-1.96685061430058	2.55524922975041
C	-2.53644193348073	-4.66422035019862	1.85921604268466
H	-3.08242678851304	-5.06019627506221	2.70862998085065
H	-1.49722158792392	-4.99030459659776	1.93173484556835

H	-2.97430331497461	-5.07015451983762	0.94543308575995
H	-0.11334477154618	-2.71703612332133	-1.88189067735110
H	-1.49078323886367	0.74101642676293	-0.08192443672438
H	-2.63701427446015	-0.51233820597096	1.69120553797594
H	-1.18915742077274	-4.14731806463412	-0.19961429857868

TS connecting acyl-DMAP to carboxylic acid product

H	3.46088761029340	3.10702268656788	-0.18392680373281
C	3.25691938983151	2.53885749762589	-1.09454497086498
C	3.47694367604127	0.32777739009544	-2.26713826984393
H	3.84820790307796	-0.69769895141936	-2.20577701029920
H	3.71355199098686	3.07063006607208	-1.93095700366011
H	2.17785454750518	2.53673868234984	-1.26338416057127
C	1.36919317814022	-0.44226298696681	1.50336576947243
H	3.93293920373373	0.79869919008431	-3.13960822696969
H	5.78916057040275	1.69870849439473	-1.72381416949673
H	5.63930997107451	1.75029568910870	0.03614542375824
C	1.76325020702939	0.26542224853049	0.28982458068022
O	1.27909916315374	-0.41264333759323	2.64100555738620
C	3.30284186559593	0.39596147861488	0.25986382352984
H	5.79270779234675	0.19555945571919	-0.79487064181755
H	1.38751215667879	-0.26339640928767	-0.57818147130141
H	1.27257957001328	1.23924571556412	0.36034783322777
C	3.82721404829484	1.11866658894190	-1.00075304150139
H	3.62672200458192	0.94055566281112	1.14867437582022
H	3.73911107033909	-0.60332043445730	0.30384399100940
C	5.35433042635774	1.19387732131172	-0.85942452524315
H	2.40018548405662	0.29238465618555	-2.44594982325430
O	1.54184914636997	-2.31802347118995	0.87377080602167
N	-0.88631606060782	-1.12771931134286	0.79056458075607
C	-1.70463780227216	-1.12412935054733	1.85752345439738
C	-1.45297163069776	-0.88445423728186	-0.40569424459375
C	-3.05758238352804	-0.90100185076708	1.78854876002718
C	-2.79322860735686	-0.64814418031347	-0.58711486363716
C	-3.66487209346428	-0.65044746707293	0.53167049883789
N	-4.98738448596080	-0.42496359995119	0.40831025341333
C	-5.57318947323703	-0.17430144349824	-0.90374524836436
H	-5.15517287641357	0.72676644892459	-1.35937515832194
H	-6.64356970664299	-0.03551402594000	-0.78994927669716
H	-5.40499759893370	-1.01711374474920	-1.57826579285225
C	-5.84796842439923	-0.42817688153077	1.58609898988520
H	-5.82867396314103	-1.39896562664865	2.08718747627761
H	-6.86765603780022	-0.22223688736475	1.27670761901134
H	-5.54258911484924	0.33993197411815	2.30059620696680
H	-1.23780954147972	-1.30722116895946	2.81906957497324
H	-0.78946085000493	-0.88780766551335	-1.26316369031149
H	-3.16064510151420	-0.46610320860909	-1.58453825049958
H	-3.63676025720668	-0.91877173777799	2.69807889032159
H	0.55516724284159	-2.29091042413690	0.76492893099769
H	1.72794779076325	-2.89177284410113	1.63404924706288

TS connecting acyl-DMAP to ester product

H	4.20769414355725	-1.17648203344682	-1.02780253979992
C	4.20678391604908	-0.97657311623427	0.04635074451048
C	4.12044842348363	0.78087964008581	1.83891060462328
H	4.05940037715516	1.84923890755806	2.05942315094588
H	5.11069787472824	-1.41756000832097	0.47031775365972
H	3.35505358964222	-1.49602844148084	0.49036560129707
C	0.54780898228763	1.35696488881415	-0.67280175722549
H	5.02154855338884	0.39117530925942	2.31588053811116
H	6.33223974272644	0.74456506181815	0.21734865380895
H	5.51357064147431	1.01506498981914	-1.32509036787563
C	1.60171025411361	0.68225204295073	0.09869425869760
O	0.08101369644227	2.40379778636022	-0.81244402037596
C	2.97462324517574	1.19580445670214	-0.38302937734033
H	5.45481717510213	2.25271375970457	-0.06224474933843
H	1.41884261630328	0.93977213559957	1.14288871187622
H	1.51943280929882	-0.39283503261173	-0.02231597492436
C	4.17390914541545	0.53106685238992	0.32670922282501
H	3.06018078684888	1.01750296128728	-1.45724558153952
H	3.01894224125758	2.27543102758859	-0.22604446578452
C	5.44367978027685	1.17623681539604	-0.24690827503169
H	3.26624664038254	0.28628542313316	2.30612884109997
C	-0.56924398039054	0.06338889618630	-4.38302632663144
C	-0.01238170068329	1.04031060155878	-3.37398106891349

H	-1.46009292703661	-0.43537533558920	-3.99583016344018
H	0.17176795743473	-0.68916848098534	-4.65253471305537
H	-0.85321976361702	0.60865131011769	-5.28457357478294
O	0.40317372331710	0.32076084444756	-2.16719029805969
H	-0.74485726066197	1.79459327819106	-3.08662945351201
H	0.89006014053379	1.53247073545491	-3.72836923166562
H	-0.36481090666061	-0.16859348305364	-1.79320252739443
N	-1.27142270423821	0.00959239304272	-0.06258093866357
C	-1.28875505678590	-1.14035879253421	0.63405810211178
C	-2.40132295846820	0.73590458223709	-0.06493733015446
C	-2.38544774032250	-1.60381602995419	1.31884733034669
C	-3.55413726976335	0.36308574588985	0.58118745075410
C	-3.58729215177602	-0.85089939895121	1.31337436020540
N	-4.69155877707144	-1.26163985316059	1.96777206715429
C	-5.90675682548425	-0.45563594988480	1.93948538241610
H	-5.74544968468438	0.52332323247873	2.39798763728217
H	-6.68342775927431	-0.97133299004907	2.49508684660200
H	-6.25745759699997	-0.30807878775991	0.91553584817525
C	-4.67448000132982	-2.50110061704934	2.73628357887096
H	-5.64774137256197	-2.64364004037946	3.19503810922761
H	-3.92208878919835	-2.46522422461505	3.52782237630440
H	-4.46648028452498	-3.36131835432795	2.09533058807970
H	-0.37187598552932	-1.71768161218187	0.63287609633744
H	-2.36618784550489	1.67211567242369	-0.61109400286130
H	-4.41534941958837	1.00984061572743	0.52612928333016
H	-2.31180769423929	-2.53944738365235	1.85004359971692

2.7.6. Benchmark: Geometry of Key Intermediates

A-TS6 PBE0

Co	-0.56834478798037	-4.10880873341117	-1.81963792743718
C	-2.17607716319625	-4.70410078275061	-2.31147860751319
H	3.18168519824922	-1.98855559463092	2.33594375976360
C	2.65255698925051	-1.08947736566468	2.00800672444753
O	-3.20895675260482	-5.12982037761083	-2.55030673208519
C	0.06873706982800	-5.62014584490301	-1.22424366400925
C	2.38876899164254	0.61464221286823	0.20670076158070
H	2.73698239730779	0.97208394375324	-0.76728724018927
O	0.40793260460904	-6.58595304353778	-0.70949669378411
C	-1.12312155065444	-2.75795945926591	-0.84385882790287
H	2.80023975464875	-0.31460128021277	2.76532259804846
H	1.58472324904908	-1.31707273824775	1.97617340738578
O	-1.58229368733797	-1.99838008594087	-0.11548272646532
C	1.61696724824731	-3.49094115884976	-1.53550143170153
H	2.51277888977856	1.42993091332586	0.92485054164611
H	4.77233433922509	0.56244796715755	1.53931545549402
H	5.23985454406050	-1.09460967779065	1.12965961923798
C	1.67491292025965	-2.21492604431990	-0.70413863046913
O	2.46772922257052	-4.35915876017165	-1.36444201697123
C	3.08760973609853	-1.73713628331686	-0.39178431409901
H	5.05831617990689	0.11508376847592	-0.14916785315298
H	1.07020663393749	-1.43423448675905	-1.16264146209506
H	1.18354299150077	-2.48208031774079	0.23327865799977
C	3.17573023600534	-0.61488271772013	0.65430709579837
H	3.66571217927084	-2.59487706882127	-0.03729287236079
H	3.58379906753952	-1.38623594710232	-1.30114325376853
C	4.64737207893741	-0.23565205822687	0.80211848035815
H	1.31963416415105	0.40324065656747	0.12896969079997
N	1.64333551432250	-3.13715827294480	-3.28230090446788
H	0.48290200804772	-3.16029933385043	-5.01195283894333
H	-0.42220272431448	-3.53737065112679	-3.57893939016189
C	2.36031421225504	-0.78415526095805	-3.75272396646903
C	2.72315020993273	-2.25577942611444	-3.77270652692774
H	1.17709678666059	-0.75901026929719	-5.55214282846025
H	2.93855570662292	-2.56005854736876	-4.80485688075611
C	0.37037774496875	-2.85958846130293	-3.96201218220440
H	3.61068540131695	-2.47243924881293	-3.17749542361978
H	1.94496006217419	-4.09402214772095	-3.57601384676515
C	1.05289038177419	-0.52966586003602	-4.48717347498449
C	-0.04556771301784	-1.40627471833598	-3.90588528073681
H	0.77533670092759	0.52443037389044	-4.41650613942912
H	2.27396008217296	-0.43067091038745	-2.72329211710579
H	3.18015566884454	-0.22500343810786	-4.21041311834522

H	-0.25344211129474	-1.11143079108428	-2.87497045784840
H	-0.97848258167296	-1.29276974588306	-4.46271558647820
N	2.81601855984049	-5.58092321899013	-4.32014658394518
H	0.09002139903813	-6.92475446691603	-6.68797932918102
H	1.58867746988846	-7.81380728645702	-6.45774442323804
C	0.82282916763019	-6.94255320873630	-4.65047737388849
C	3.13677167748320	-5.59699151031850	-5.74324259008396
H	2.10639398899976	-5.72040517533183	-7.62426064150240
H	1.98544691965913	-6.76839971646997	-2.83360800767026
C	2.14080803723415	-6.80951598329553	-3.91157393870843
H	2.76484440646885	-7.69262858938207	-4.12673264690791
H	3.66512604734990	-5.47855248170084	-3.77643132889381
C	1.85970646893831	-5.68702536588246	-6.55959023822616
H	3.68891927631493	-4.68622975174895	-5.98904523912665
H	3.78427745294583	-6.45317988267162	-5.99520146066286
C	1.04556586559099	-6.90971997878468	-6.15664696668538
H	1.26901435540934	-4.77927882048556	-6.39327477208878
H	0.32820757356639	-7.87073238061114	-4.35254202165740
H	0.16390323962027	-6.11942310792782	-4.35147001238478

A-TS6 TPSSh

Co	-0.50255237100607	-4.30116013581613	-1.41941523155452
C	-2.14167253966035	-4.96506240724349	-1.70940992290812
H	3.59100582956574	-1.69112867409740	2.19292921916973
C	2.98045025725115	-0.84520466845081	1.86393933094077
O	-3.17804793002978	-5.45425125884610	-1.82185418343435
C	0.32733101955687	-5.80077073985716	-1.06020333666258
C	2.42205198777540	0.70569199525703	-0.02414396291218
H	2.65358483305317	1.01197652605892	-1.04896268916502
O	0.82893195482084	-6.78125666058121	-0.70937577739127
C	-0.94339188474210	-3.03822065125752	-0.26974302690503
H	3.16721848195546	-0.00721165916463	2.54158157512260
H	1.92870283916074	-1.12398778303724	1.96506589068642
O	-1.31848806084897	-2.33804436385959	0.57163674958959
C	1.72649488177794	-3.53590464828917	-1.42894661291089
H	2.56528062923333	1.57405075413694	0.62524089318062
H	4.93968036092681	0.88218282454574	1.03957862497270
H	5.46690721112360	-0.77433305400390	0.69713713619335
C	1.78955955652076	-2.24184850730099	-0.62463871179301
O	2.64573642092436	-4.34441312615284	-1.37226904846174
C	3.20315901473376	-1.65787319779064	-0.52707895897170
H	5.07029400404730	0.32229282169157	-0.63655465950351
H	1.07139907649883	-1.52158739624341	-1.01040179583790
H	1.45883433005454	-2.52110790698524	0.37658459451331
C	3.32999148577579	-0.44795089574025	0.42332325801294
H	3.87407227157174	-2.44895921914844	-0.18217483294962
H	3.56050315601229	-1.35559400605592	-1.51534533139357
C	4.79017705680220	0.02370520367644	0.37825582005225
H	1.36600538555400	0.42819098639757	0.02079755950971
N	1.44123305356420	-3.21952683256477	-3.19505046340248
H	-0.00573225864824	-3.27404707101552	-4.70322729577038
H	-0.64175094033877	-3.67223327154487	-3.13305998869390
C	2.01064003147052	-0.84687757588461	-3.79010134488438
C	2.40876376622546	-2.31567943387778	-3.87471131528619
H	0.52201382100622	-0.86282845284114	-5.35722907695434
H	2.45066005205257	-2.62219555084207	-4.92672961006670
C	0.05397902444036	-2.97360847650041	-3.64984383858415
H	3.38725653911220	-2.50514427844095	-3.43273705281811
H	1.67974889352957	-4.19829394263012	-3.49903488860981
C	0.58236947982125	-0.62593132197172	-4.28868236252360
C	-0.38189049109179	-1.52532050498995	-3.51446842048512
H	0.29899656401943	0.42259632552376	-4.17167765294348
H	2.09054171622155	-0.49600945032660	-2.75995075229242
H	2.72361494205396	-0.26702611296604	-4.38170946787435
H	-0.40587351802571	-1.23528949715669	-2.46196983951887
H	-1.40085894911159	-1.43390007232005	-3.89741714008947
N	2.08763284667499	-5.92341704364607	-3.95274351153708
H	1.74597682598260	-6.17518553921823	-7.80302386435603
H	2.28746233631451	-7.55007696040267	-6.84486649817676
C	0.70515672206047	-6.42268687085779	-5.91368608961590
C	3.33245575697004	-6.06311070967370	-4.72386221595421
H	4.01380805621492	-5.79016252408227	-6.74821803447405
H	0.14551961247635	-6.67301385550012	-3.83379106117500
C	1.02398429964505	-6.79704014115484	-4.47035977875876
H	1.32566474721342	-7.85551675649420	-4.42714220489504

H	2.27700688338792	-6.15193898267727	-2.97992654345599
C	3.09004327004569	-5.66619160324448	-6.17632449892594
H	4.08856020337167	-5.42327175502727	-4.26370680057031
H	3.70327141199329	-7.09969791959771	-4.68935156454601
C	1.96247269526158	-6.50470466587000	-6.78298532444840
H	2.82020378313090	-4.60523968713328	-6.21663932628768
H	-0.07459032066366	-7.08537508665726	-6.29912797815938
H	0.30832988520706	-5.40184253025196	-5.93752276305170

A-TS6 wB97X-D3BJ

Co	-0.58529790521780	-4.09613771256190	-1.84411016864641
C	-2.21353496840028	-4.69426263834120	-2.30415758360908
H	2.99973724916403	-1.96298293157726	2.34370564462589
C	2.54039290710581	-1.03524779821815	1.98934591038690
O	-3.25116634155340	-5.11158696890228	-2.53949511542960
C	0.09784317638373	-5.61337038316905	-1.29937198189302
C	2.43613970549420	0.65745864226348	0.14943595845902
H	2.85924403412967	0.99373678747927	-0.80256099491937
O	0.47273074186905	-6.59009372700047	-0.83004972258754
C	-1.10307947158857	-2.76673835545066	-0.81118187634668
H	2.69719883343847	-0.26567949402011	2.75103454974141
H	1.46365936963236	-1.19917619946730	1.90323755193764
O	-1.52484646567590	-2.00300618311946	-0.06351931753768
C	1.65231756849423	-3.47256662622205	-1.59688961433248
H	2.53935986120166	1.47472011220118	0.86942717434113
H	4.73887565191659	0.55396048808816	1.61820646558788
H	5.18003052306333	-1.12568645631996	1.26628606150561
C	1.68605050376296	-2.21085642199573	-0.72660876337982
O	2.49874123883417	-4.35649191294110	-1.37921076539984
C	3.09912782028394	-1.72965800639732	-0.38387997057787
H	5.11216539626218	0.06513819592990	-0.04330517382045
H	1.08172701989027	-1.41957263079074	-1.16596868023756
H	1.19727416325128	-2.50485203512608	0.20110501234715
C	3.15883096912160	-0.59515942819385	0.65784878937349
H	3.66249795921734	-2.58587521596025	-0.00435487568342
H	3.62360925638659	-1.38544828337428	-1.27936943353594
C	4.63574478900493	-0.25489518074487	0.88871362970162
H	1.36789461229495	0.47612947685286	0.00538450339244
N	1.66417469822195	-3.17193586599122	-3.22635638106635
H	0.52424129493612	-3.17481576301080	-4.95806367683997
H	-0.41178772142859	-3.58338592467800	-3.56911948175761
C	2.37940702012751	-0.80211434139997	-3.72178011400149
C	2.74972778765012	-2.27887323486431	-3.72735637236805
H	1.18104912684159	-0.79806871713729	-5.51922501331030
H	2.95300498871669	-2.59939892974670	-4.75459824164112
C	0.38173634144989	-2.88685169600409	-3.91067463160615
H	3.63535220222641	-2.49206853926347	-3.13037756015785
H	1.96407721884399	-4.11823937652402	-3.55429533604590
C	1.06305433686965	-0.55529729841930	-4.45664670882680
C	-0.03683723679348	-1.42818981830624	-3.85603637262808
H	0.78739891640437	0.49984117026718	-4.39337939667888
H	2.29909674244009	-0.43274851399002	-2.69910962099029
H	3.19680054387386	-0.25221131482636	-4.19544472675246
H	-0.24106855888076	-1.12575557488764	-2.82808627364231
H	-0.97126625928572	-1.32308145668263	-4.41234966954381
N	2.83995542079927	-5.54565198418349	-4.39500957879865
H	0.02583274869012	-6.98382002987863	-6.62323284054439
H	1.55367243440096	-7.84015807312100	-6.44401294721517
C	0.84917781932839	-6.94907276668950	-4.61465582678195
C	3.09391547529367	-5.57659210814000	-5.83978824720239
H	1.97469215887127	-5.75101369424267	-7.67081833974998
H	2.10340199242789	-6.73832155216200	-2.85625580081072
C	2.20535613583221	-6.78993814291377	-3.93978509219362
H	2.83468997177194	-7.66098332846284	-4.18203539687531
H	3.71748508012925	-5.43210906136164	-3.89944138575241
C	1.77612274150510	-5.70431997494207	-6.59621753233591
H	3.61381982882951	-4.65771368664360	-6.12393025693093
H	3.74509580575957	-6.42421563198570	-6.10626207043328
C	1.00419867443817	-6.94233558563599	-6.13612409621527
H	1.17351085537780	-4.80749348568249	-6.41540681543400
H	0.37917410176816	-7.87721254739035	-4.27793284109858
H	0.19835911479516	-6.12522026401936	-4.30121456723262

A-TS6 R2SCAN

Co	-0.55575663282947	-4.19993365919983	-1.80297733362721

C	-2.13638611041639	-4.82010802124455	-2.35121900113331
H	2.89606782211116	-1.86985410041026	2.37235230329653
C	2.50640757707861	-0.93602793804650	1.95564163956414
O	-3.16330788471229	-5.27846032843193	-2.60127168260157
C	0.09762284939086	-5.68961098940911	-1.17850247385761
C	2.55065743234043	0.65395852224691	0.02353782065718
H	3.05277155414675	0.93921191999304	-0.90599275585643
O	0.44061710013683	-6.65172060126881	-0.63734470491678
C	-1.12587393384563	-2.88858968233658	-0.78866883939661
H	2.68643857122727	-0.13967898552586	2.68341439536083
H	1.42442945676811	-1.03935383035724	1.84218782507440
O	-1.59605888375813	-2.16061710657656	-0.02050595326504
C	1.65990163707362	-3.53627982421410	-1.51776748387359
H	2.63284807902671	1.49403906846814	0.71921378885483
H	4.79267011067099	0.53882583422299	1.55911854162202
H	5.15883957939739	-1.18213714902206	1.35415262516442
C	1.69493523979913	-2.28017367328244	-0.65533312627828
O	2.51921484785478	-4.39730899996597	-1.39976480531998
C	3.11248813388399	-1.78920209678907	-0.35309253400437
H	5.20011786640666	-0.09526567716784	-0.04355472359506
H	1.06807114403447	-1.50328169366981	-1.09149408991352
H	1.21945563311952	-2.58078026325593	0.28157087597582
C	3.19104016406650	-0.60046349419855	0.62694197442611
H	3.67803523057770	-2.62808007060256	0.06455803791885
H	3.62412960607771	-1.50062653883407	-1.27710482848797
C	4.67431467996853	-0.31836715600883	0.88990406098461
H	1.48877372284568	0.50593623232762	-0.19105005837695
N	1.63128003198493	-3.13227277680076	-3.31027793808788
H	0.42262047245548	-3.11577248399865	-5.01868503255451
H	-0.44176223490702	-3.53758850170612	-3.56119149782413
C	2.32671505155200	-0.75904712667328	-3.72197769662268
C	2.69878321431151	-2.23002664790654	-3.81104254837909
H	1.09934090597120	-0.68572408142795	-5.49589645591981
H	2.87493773265670	-2.49802528650811	-4.86279594826670
C	0.33429924510252	-2.84587166023603	-3.95618075767137
H	3.60770609591194	-2.46783135860527	-3.25509779750447
H	1.93796966163762	-4.08766777983135	-3.62319655132379
C	0.99664483955066	-0.48885726024571	-4.42137667217703
C	-0.08858420573065	-1.39312615312241	-3.84143599331359
H	0.71413000027680	0.56110619954228	-4.31040552157416
H	2.25728802073028	-0.45033203824300	-2.67594170900518
H	3.13173773780724	-0.17244129306987	-4.17348936341617
H	-0.27202511027431	-1.13505695829903	-2.79479048342750
H	-1.03538585166283	-1.26500638890263	-4.37254543432659
N	2.83403871387474	-5.55599082858342	-4.33575258201247
H	0.08357966598323	-6.89258631655437	-6.69596040924957
H	1.57374069799094	-7.79799301075732	-6.45280529011108
C	0.81494138160344	-6.89435077901873	-4.65232226653578
C	3.15709622632262	-5.59558997164341	-5.76765038456617
H	2.11332650964599	-5.71459409325311	-7.64677575471115
H	1.98944233461845	-6.72741839986711	-2.83510341800056
C	2.14003560373336	-6.78114428162172	-3.91409371924149
H	2.75249431922285	-7.67374862579701	-4.12838095780514
H	3.68918247732900	-5.46501785438981	-3.79446696286081
C	1.87170882061160	-5.67104584563180	-6.58062865498078
H	3.72670392826738	-4.69708866576272	-6.02110735195076
H	3.78667253702013	-6.46983780210797	-6.00725052634305
C	1.03954703379176	-6.88361234894904	-6.16397225494240
H	1.29467836605168	-4.75320041015528	-6.41440439908663
H	0.29818319269221	-7.80724547747648	-4.34223762583719
H	0.17635799142450	-6.04961138983564	-4.36303553476495

A-TS6 r2SCANh

Co	-0.56349678568528	-4.15830975560997	-1.81385859192683
C	-2.15703755402436	-4.76567059915473	-2.32975664030043
H	2.92255273740129	-1.90440280795021	2.35744707255805
C	2.52410168266777	-0.96771783386919	1.96099648269600
O	-3.18599084702824	-5.21149566472773	-2.56503156758955
C	0.08500024242703	-5.66027609642896	-1.22158553804211
C	2.53530512418781	0.64965650099771	0.05847985290582
H	3.01606361916144	0.94601675602287	-0.87666383814302
O	0.43020909154603	-6.62703936325099	-0.70405297893436
C	-1.12185529283387	-2.84883986416934	-0.79479983601823
H	2.70983799117407	-0.18325121861511	2.69739132796604
H	1.44312017377353	-1.07409167579353	1.86074507496671

O	-1.57980396421239	-2.11871268232627	-0.03064898675076
C	1.63360558663816	-3.51791286312165	-1.52690331812618
H	2.63190346708831	1.47996960554582	0.76107022820016
H	4.79511036448914	0.51479930263291	1.56249507192044
H	5.16476953533299	-1.19650830632236	1.31420004892186
C	1.68186575697685	-2.25858914235440	-0.67015015525385
O	2.48991524479488	-4.38120959811689	-1.39164929371112
C	3.09918765836050	-1.77957898235887	-0.36502197137984
H	5.17978327657638	-0.08081087130284	-0.05692435113847
H	1.06388987434722	-1.47715698609966	-1.10642732293106
H	1.20510626080260	-2.55134807818279	0.26597867568581
C	3.18748717550631	-0.60941880754852	0.63100697988521
H	3.66063992870276	-2.62612239765891	0.03674808728262
H	3.61003825180588	-1.47863930558543	-1.28300900549384
C	4.67006925431508	-0.32647856506954	0.87757671477115
H	1.47101958629362	0.50361892064671	-0.13429696598830
N	1.62459662699081	-3.13193932316922	-3.29043953429602
H	0.44176486279197	-3.12286601642460	-5.00839749560982
H	-0.44283238323719	-3.53658513923979	-3.56904579519679
C	2.32882680988837	-0.76792912097863	-3.72088596475872
C	2.69675783717328	-2.23839323370514	-3.78787461120251
H	1.11945944447616	-0.71058727557420	-5.50295503164074
H	2.88254771530511	-2.51839179058885	-4.83214476157461
C	0.33838005448465	-2.84740608368060	-3.95120753120223
H	3.59731712301825	-2.47141405255053	-3.22095711170701
H	1.92916194292079	-4.08408980328667	-3.59857105640693
C	1.00846664569238	-0.50213303037440	-4.43356303678433
C	-0.08181193737839	-1.39540434005604	-3.85450092607642
H	0.72874385738839	0.54784624278711	-4.33687161361432
H	2.25227885307228	-0.44533496901834	-2.68181437105413
H	3.13841890255393	-0.19175391185380	-4.17266056081188
H	-0.27331932144212	-1.12571546079675	-2.81456108967562
H	-1.02184041110779	-1.27136606859646	-4.39431500544171
N	2.83126075841159	-5.54717276468163	-4.34402253661168
H	0.09912450259622	-6.94112054006364	-6.68188338339083
H	1.60520942271080	-7.81417353835961	-6.44293453499693
C	0.83871747052933	-6.92042384989195	-4.64562720615466
C	3.14462716795776	-5.58296333057627	-5.77352505435686
H	2.09583229234040	-5.72685689109423	-7.64361206869379
H	2.01722044634361	-6.72574328683223	-2.83858243793314
C	2.16284964010644	-6.77910359489429	-3.91617987513810
H	2.78854553407703	-7.65986083189499	-4.13022014482602
H	3.68577620748613	-5.44018250460341	-3.80968771367528
C	1.85930100745516	-5.68599021149292	-6.57830389717171
H	3.69353312475756	-4.67587946858097	-6.03346458611099
H	3.78916141772314	-6.44286068445263	-6.01531359822291
C	1.05532956115575	-6.91128702347860	-6.15526765414841
H	1.26513532858107	-4.78184022308512	-6.41142738299224
H	0.34256931078652	-7.84043890692774	-4.33012357966557
H	0.18638274380465	-6.08868659221024	-4.35774010488830

B-IM7 PBE0

Co	2.67063768303888	-1.84200704802837	-0.39711611735771
C	3.34730969672164	-2.30051690225257	-1.97446717245082
H	7.15892468136735	-3.71074983080052	2.86287234638561
C	7.19120116127927	-2.65503017670132	3.14821747253826
O	3.78833925750792	-2.55233076998890	-3.00266498918458
C	0.95566841534153	-1.52342590540043	-0.67395067709077
C	6.92979546418078	-0.29461784415814	2.38281666065184
H	6.70081746150897	0.36631252670535	1.54202375681566
O	-0.05043409487002	-1.05512743734010	-1.00471271110897
C	3.33962954728200	-0.26203516400440	-0.17035201272647
H	8.18197183157278	-2.44900692902424	3.56325389098356
H	6.46128125406592	-2.49692358659952	3.94608937948432
O	3.73650178056938	0.80874397961909	-0.01794512988854
C	3.04818276309093	-2.40331073146889	1.50690330048730
H	7.91353437279796	-0.01803993330697	2.77284250102842
H	9.00676679947955	-1.73064069637592	1.33678161244469
H	8.04828800824013	-3.00798809964918	0.57168371184783
C	4.35440575961343	-1.94483392186086	2.13737264683624
O	3.08895918719626	-3.43170034003653	0.72536127225176
C	5.58731075284242	-2.11639814319048	1.27076566916956
H	7.87071170146082	-1.33074610954241	0.03899231958863
H	4.25056402121975	-0.91128352358465	2.47159971064047
H	4.48535974489373	-2.54877643321453	3.04181147611518

C	6.91997091365432	-1.75706129694944	1.94345154849489
H	5.63361709208911	-3.15585582291036	0.93207045446567
H	5.48461468306740	-1.50906230626992	0.36818761811785
C	8.02692751576462	-1.96900962354791	0.91315092274982
H	6.19704879528264	-0.10407280987699	3.17071803567099
H	2.05026533256867	-0.54035972634783	5.91420768394571
H	1.22896332970564	-0.54267628632741	1.69036646385739
H	1.04101869395608	0.59562141147072	3.89665968895237
C	2.01931203389211	-3.14854921531710	3.63841998766538
H	0.63522337533146	-1.39154381052505	5.30553579556482
H	2.42303285563806	-3.03447371874856	5.73266525452132
H	0.92059575195312	-3.48640684484472	1.52161931699333
H	0.12181460267877	-1.34763558108660	2.80195552526210
C	1.17929572411192	-1.10994890768033	2.61759294851622
H	2.69366040949484	0.08001589602284	3.59469684233486
H	3.54963509820513	-2.10130988532528	4.76040679827515
C	1.68135433294832	-0.28514765811617	3.79316721375201
C	1.67013688545844	-1.11539694903746	5.06597999595697
C	2.49687995129565	-2.37491634843108	4.86251819554162
N	1.86987327155016	-2.37951559299621	2.39325316220272
H	1.02854694267420	-3.55795500948767	3.87977067173729
H	2.67090446345234	-4.00117564273095	3.43238602665439
H	-1.19780012037676	-2.77653130194546	0.72897319772369
H	1.82772539379276	-5.60850078154642	0.95246466240419
H	0.81282207396683	-3.75594230392452	-0.06123423504519
C	-1.35858989703040	-5.85857442257599	2.30161712333160
H	-1.49917108657158	-4.34622558391948	-0.02767349627848
H	-2.73143113223042	-4.19090768841570	2.10876211367208
H	0.63426243702639	-5.66778179658635	3.09958616727566
H	0.34922949097791	-6.06316086215792	0.08480581883211
C	0.74090589136179	-5.54418290421544	0.96347276893039
H	0.35610210589582	-7.18428133673599	2.26130648129684
H	-1.24785739007678	-3.81098436523909	2.97195614645954
N	0.40060100655455	-4.11148731507046	0.80886351117270
C	-1.05537352782012	-3.85072209063011	0.83956314332471
C	-1.65535724673102	-4.37612481587574	2.12630645783612
C	0.14025752666394	-6.11395976591878	2.23123245561154
H	-1.86314904090824	-6.42430410213883	1.51003089494681
H	-1.75873763045398	-6.21585527384069	3.25299181851476

B-IM7 TPSSh

Co	2.67021273315248	-1.81526214469305	-0.37786567047602
C	3.38320677681198	-2.26103035618109	-1.95411010902428
H	7.19039931186426	-3.70482927901098	2.88356259615114
C	7.21886901395589	-2.64489275357251	3.15439127052236
O	3.85040494642741	-2.50070136952363	-2.98302930253281
C	0.94461588219281	-1.51842103235695	-0.66729225028699
C	6.93583516118253	-0.28611618915030	2.35016404178782
H	6.70415663885546	0.35908692702833	1.49795377519583
O	-0.07120735368040	-1.06211807587361	-1.01626966035048
C	3.33438896040625	-0.22312224529803	-0.12672562001397
H	8.21202856223471	-2.42447999875545	3.55690626490433
H	6.49205241104291	-2.47749811459865	3.95369558527867
O	3.73153926565089	0.85409493262682	0.03853833341231
C	3.04357560930232	-2.40977299711744	1.53083759217335
H	7.92014503941813	-0.00287805012033	2.73482851500217
H	9.01498004170784	-1.73292926913459	1.29828949861762
H	8.05604663092498	-3.03097452990014	0.56510006097072
C	4.36074916065837	-1.97553117145816	2.16737870742229
O	3.07322769655054	-3.43108681136338	0.72326802871589
C	5.58646905311422	-2.14281631320365	1.27606094696348
H	7.86033012751129	-1.36261859366894	0.00568907638446
H	4.26934016405001	-0.94702574319880	2.51905046978981
H	4.49219027131983	-2.60041091846715	3.05781959485049
C	6.92994722356172	-1.76297311770052	1.93226831722852
H	5.63777554144065	-3.18452562889558	0.94586957693904
H	5.45906620603344	-1.53988377593746	0.37389537099322
C	8.03268074803781	-1.98581753800839	0.88798444122014
H	6.20217030831726	-0.08721956284320	3.13560953969044
H	2.07115655169458	-0.50183429343370	5.92432828031650
H	1.21483442285324	-0.54919133651794	1.69430773163280
H	1.06216652908107	0.62637083362093	3.89694372301830
C	2.00692427630135	-3.14906663605433	3.67725030917413
H	0.64507661737052	-1.35113441163270	5.33121222079605
H	2.43467326440972	-3.00272926101482	5.77069787731169

H	0.90283735656016	-3.49474882970747	1.51532087028277
H	0.11114894205450	-1.33335025306434	2.83087260878691
C	1.16536822718290	-1.09910254172390	2.63148396466620
H	2.70761933130063	0.08493052098464	3.58421491187405
H	3.55561986708790	-2.08867894982669	4.76881918580599
C	1.69215969736239	-0.26253193431870	3.79673453709645
C	1.68049891093839	-1.08343977186905	5.08519730758468
C	2.50366690395407	-2.35615184677207	4.89010043235818
N	1.85190777070423	-2.38651104870423	2.41310099400193
H	1.01420204495909	-3.54707464740094	3.92445392184361
H	2.65598135735778	-4.00288886157262	3.47107309093841
H	-1.22371240809174	-2.78646589779468	0.70870504664560
H	1.82487400682579	-5.61533310843609	0.92653257185384
H	0.80440887874526	-3.74851025794343	-0.07836144850051
C	-1.37158849761262	-5.88481448232245	2.27856044069130
H	-1.51440449451782	-4.36033551570490	-0.05187640438932
H	-2.75458192515463	-4.21698902388331	2.09271109089605
H	0.62544699757782	-5.67598343045081	3.07927255742867
H	0.34334152580160	-6.07158455887739	0.05744621026851
C	0.73831385495741	-5.55931384588663	0.93752629917812
H	0.35898052246231	-7.20017816646321	2.24448212903750
H	-1.26926659564127	-3.83237976881569	2.95610910836686
N	0.39046843213949	-4.11361569084190	0.78981220204342
C	-1.08090341532455	-3.86003253480643	0.81672410643815
C	-1.67715286494547	-4.39511600290872	2.10977644031592
C	0.13720609402939	-6.13096806727478	2.21270290478236
H	-1.86875520621426	-6.44871626051602	1.48140189243280
H	-1.77085494903980	-6.24717185769372	3.22847799219593

B-IM7 wB97X-D3BJ

Co	2.66813940922632	-1.78019185875299	-0.36704404319154
C	3.50632644165005	-2.13373364112619	-1.91131702698150
H	7.14375518064778	-3.81716430341217	2.69530483632156
C	7.20297051386772	-2.78516768812297	3.05527391030265
O	4.06271378551244	-2.33048885698972	-2.89401416341331
C	0.94538826867178	-1.54579299060636	-0.73469459770218
C	6.95044452284984	-0.36455396671485	2.46807072689221
H	6.70130974247391	0.35720865877012	1.68412627670586
O	-0.08015341862220	-1.14169291246620	-1.08669078557002
C	3.23359630734492	-0.17957481638034	-0.00816439449573
H	8.20684917697927	-2.62502102553240	3.46051540915273
H	6.49252826843763	-2.66977466509327	3.87786892320520
O	3.58700065075753	0.88404720847707	0.26095817495749
C	3.02858023356353	-2.38783325377411	1.49192045483947
H	7.94636469522726	-0.12147387949327	2.85102398714670
H	9.00519820677304	-1.73888028976945	1.28658612868233
H	8.02685559137816	-2.95330371416051	0.44423066220805
C	4.35030752641097	-1.97724115503819	2.14062137716063
O	3.06319736025038	-3.41302134095020	0.68848915390837
C	5.57301391206886	-2.09285305528655	1.23768789153064
H	7.85558869721450	-1.23969122029602	0.03356453675313
H	4.25639947020993	-0.96519525545242	2.53635158858905
H	4.49748064621733	-2.63862115448117	2.99921546313669
C	6.92175631882268	-1.79538378029480	1.91922952029008
H	5.60407075380875	-3.10384268175524	0.82096961074652
H	5.46401732046588	-1.41493121492324	0.38855231520996
C	8.01861535716425	-1.94025615449867	0.85804627919600
H	6.23867594194582	-0.23360318395433	3.28721812073308
H	2.07235742094457	-0.42123323022968	5.84927810782868
H	1.11541345267889	-0.58332824890517	1.64945457046592
H	0.98892099534992	0.63190113777756	3.81871384399274
C	2.01933863145035	-3.11323238339303	3.65775604867914
H	0.65061365632867	-1.31108857934227	5.30934715482905
H	2.46536852870764	-2.92346939997852	5.74522728524771
H	0.91353553389547	-3.51973203937589	1.50520065087964
H	0.07786936497727	-1.39119919231488	2.82490931712465
C	1.11861616410783	-1.12246975091120	2.59315812242616
H	2.64114829510032	0.12781825001876	3.48396518649226
H	3.56250826205768	-2.01059338226680	4.71795165281041
C	1.64059922705324	-0.24225208277461	3.72663684214915
C	1.67534379410039	-1.03116846977190	5.03318437330028
C	2.51648844553912	-2.29278312823679	4.85190934814699
N	1.84828072306505	-2.37978329941211	2.38913651344073
H	1.03293870323141	-3.51455939636662	3.92656847608926
H	2.67147195844887	-3.96965360616749	3.46946608734853

H	-1.19070243382474	-2.83464801998550	0.64057784587065
H	1.84900583899610	-5.65111194132576	1.02251542260613
H	0.84903618181053	-3.84178524784233	-0.07184667678622
C	-1.36403532202265	-5.85935716362732	2.33771442048563
H	-1.47358559205406	-4.43940320373624	-0.05643830527666
H	-2.73741043904546	-4.19577630609423	2.06632125463379
H	0.62060963688837	-5.62435677694161	3.15783467849658
H	0.38103353794478	-6.14481486506083	0.15266032015911
C	0.76193180788147	-5.59158530247444	1.01486368674500
H	0.36143337171670	-7.17953817564837	2.37993748928616
H	-1.25827426591480	-3.78031222433640	2.92652158560256
N	0.41766977016945	-4.15943802815582	0.79963140265574
C	-1.04684329283329	-3.90316791600583	0.79468369406713
C	-1.66116667169746	-4.37881101924288	2.10194106273112
C	0.14312165709458	-6.11183529914724	2.30318927733428
H	-1.85252684386324	-6.45486848360697	1.55794605154336
H	-1.77824282038627	-6.18072649304406	3.29616697098365

B-IM7 R2SCAN

Co	2.67212634962762	-1.84343568080867	-0.43610077309167
C	3.37735462844222	-2.29726664179080	-2.00175104141492
H	7.17009036120624	-3.75012856154701	2.78739371259376
C	7.20715890070390	-2.70700952293723	3.11670642730598
O	3.84088520706065	-2.52575583488445	-3.03538097490209
C	0.95062882243081	-1.57495574697381	-0.71209709952958
C	6.94690979769375	-0.30830982267601	2.45146622787105
H	6.70371956900213	0.38808884406392	1.64319172399097
O	-0.06858212914928	-1.12577869503661	-1.06362595768895
C	3.32866690904736	-0.25629813162020	-0.19918674796508
H	8.19946809572756	-2.52139101361580	3.53866088275172
H	6.47843708406736	-2.57753194795283	3.92147430934634
O	3.72729480624607	0.82176431529908	-0.03974112639646
C	3.05907354750338	-2.40820934303065	1.49760343517847
H	7.93485070289585	-0.04507943654774	2.84108633145915
H	9.03011178104992	-1.70738946776692	1.34451523006340
H	8.07000450553903	-2.94837478335990	0.52130897438051
C	4.36388500381615	-1.93183338691059	2.12608122753112
O	3.10231921478110	-3.43572538896632	0.71080346186197
C	5.60196900058272	-2.08506793349353	1.25309052652392
H	7.89802566179209	-1.24786012155633	0.06127484634656
H	4.24584776142332	-0.90046844483524	2.46549440014596
H	4.50213721769443	-2.54136787255067	3.02753593163777
C	6.93856760676588	-1.75480333763743	1.94665716505187
H	5.64267720168256	-3.11433289691795	0.88104056469753
H	5.50624506986526	-1.44426149704580	0.37176433837334
C	8.05115721643041	-1.92462482411142	0.90698522952858
H	6.22325685921914	-0.15843033087140	3.25717978632359
H	2.02462957385196	-0.52961493469141	5.91241310575563
H	1.21883737033867	-0.54092676188869	1.67307563257167
H	1.02529233634154	0.60370003558876	3.88431749150531
C	2.01922444532594	-3.15061479744752	3.64065941334728
H	0.62031768028783	-1.39613132261814	5.29184790511328
H	2.43431628858509	-3.02074759352219	5.73556812828554
H	0.92200567835098	-3.47883841793569	1.52799308053911
H	0.11233957849838	-1.35091032770397	2.78832742047105
C	1.16896443367995	-1.10487335658652	2.60362657924474
H	2.68236416109393	0.09037447765645	3.58885902826903
H	3.54853899162443	-2.07825421918135	4.75297586301177
C	1.66887770128444	-0.27587479510297	3.78394639611125
C	1.65510629968618	-1.10847166178952	5.06138880015988
C	2.49874427851476	-2.36378592079902	4.86177329099407
N	1.86616358696833	-2.38411200400668	2.38102832632997
H	1.02476046496290	-3.55272414296544	3.88303240867626
H	2.66980019082159	-4.00540452858619	3.43451304691956
H	-1.20901742538472	-2.77398937132664	0.74261842493705
H	1.81333582349921	-5.63006190168762	0.95934345043728
H	0.79175158937025	-3.76789722272499	-0.06274266799315
C	-1.37667742826668	-5.86083854626869	2.32525334383088
H	-1.52463394817171	-4.34883143949134	-0.00700567633886
H	-2.74131108717813	-4.17743388838716	2.14809164924099
H	0.62324143911433	-5.67012873928904	3.11775354073930
H	0.32406745336230	-6.07931553833912	0.10053825493321
C	0.72604046838670	-5.56153353076319	0.97609394909625
H	0.33937756710836	-7.19378750608228	2.28908858024610
H	-1.24207012443861	-3.80907886952478	2.99463184359800

N	0.39004368983924	-4.11767472371138	0.81741423821708
C	-1.07519312568088	-3.84974163241583	0.85617752893054
C	-1.66592896621444	-4.37101825520076	2.15352293964911
C	0.12639711472071	-6.12237680947878	2.25323339877312
H	-1.88360488898647	-6.42115651428876	1.53034231784680
H	-1.77752980522897	-6.21848319336212	3.27685205327943

B-IM7 r2SCANh

Co	2.66477412448490	-1.83609562228298	-0.42067869467152
C	3.34893315959654	-2.29954830737125	-1.98921600053771
H	7.14788704421361	-3.74846299574501	2.79908306652133
C	7.19084610101552	-2.70482716508354	3.11959378962882
O	3.79752924954939	-2.54372923854559	-3.01946880160700
C	0.94820807964220	-1.54610722260373	-0.68352215222987
C	6.94364728703435	-0.31489420499610	2.43718418169747
H	6.70611459370353	0.37526564929278	1.62447469702628
O	-0.06936220065912	-1.09370051521263	-1.01466480228547
C	3.33518141651125	-0.25832840190848	-0.19401285125268
H	8.18281480414581	-2.52190849696394	3.53859767516084
H	6.46488071556005	-2.56557081101502	3.92254882674953
O	3.74044812392311	0.81223068890071	-0.04022596389055
C	3.05018272901944	-2.39265963524573	1.49916435615150
H	7.93088588788096	-0.05470824828844	2.82531271173436
H	9.01502261873847	-1.73005237462802	1.34301989367940
H	8.05022555913217	-2.97044222502462	0.53126111670064
C	4.35660890802657	-1.92382846008799	2.12532493235206
O	3.09086388038980	-3.42141683459066	0.71880228650345
C	5.59155619515210	-2.08987165999068	1.25550728555332
H	7.88677800138408	-1.27579105762157	0.05892761307367
H	4.24653366398623	-0.89184252227173	2.45923058773099
H	4.49072646358833	-2.52858456126412	3.02779373107042
C	6.92716761672189	-1.76233686773692	1.94474102611612
H	5.62653652660309	-3.12029520924888	0.89223998257481
H	5.50085931071576	-1.45722611007463	0.37039541932814
C	8.03647756391424	-1.94545307353848	0.90842504755074
H	6.22105674092688	-0.15448887877316	3.23913289530228
H	2.02602226360715	-0.53609062403419	5.91859954811495
H	1.22509546336549	-0.52236529678243	1.68793875929121
H	1.03228085256513	0.60760286313852	3.89934771715592
C	2.01754560752440	-3.13820736964252	3.63512287136696
H	0.62360130618668	-1.39550168304405	5.29403670497205
H	2.43241579109640	-3.02248369868284	5.72746643831228
H	0.92019957774730	-3.48586172956086	1.52237726372437
H	0.11750978499945	-1.33387957970055	2.79420145009174
C	1.17360307350130	-1.09204838108234	2.61244235930137
H	2.68539988019045	0.09315184552810	3.60342407031952
H	3.54521605597557	-2.07671138002152	4.75262263157593
C	1.67280773113337	-0.27127060869613	3.79524961355384
C	1.65696425783895	-1.10875053490008	5.06572362813950
C	2.49710627839373	-2.36154758806762	4.85925437327820
N	1.86627729420905	-2.36654939330107	2.38489335861979
H	1.02530520659977	-3.54106998112506	3.87681977508852
H	2.66611218827765	-3.99124956082230	3.42573307275078
H	-1.20054663560226	-2.78833752340925	0.72547552836855
H	1.82931549732171	-5.62225516200889	0.96556208840879
H	0.80986554904887	-3.77235866040780	-0.06090962978550
C	-1.36024546640494	-5.86524857121614	2.31293757463849
H	-1.50087730908707	-4.36413843571707	-0.01992777799001
H	-2.72956676147493	-4.19221939902018	2.12305339252542
H	0.63069460392456	-5.66332613479893	3.11426906433613
H	0.34945294280282	-6.07891325649270	0.10216533660526
C	0.74377084207965	-5.55824657621708	0.97691127776097
H	0.35949837243819	-7.18705678796259	2.28853525187927
H	-1.24037646204404	-3.81489214741593	2.97594039361545
N	0.40214839345010	-4.12072207874329	0.81357039520130
C	-1.06017848706735	-3.86079436315955	0.84263370281818
C	-1.65537188553198	-4.38046375427298	2.13551245633371
C	0.14148333735195	-6.11885649363818	2.24970627375411
H	-1.85931671487630	-6.42958981334948	1.51869244525812
H	-1.76373843522696	-6.22146923945751	3.26163083358685

2.7.7. Proton Transfer to $[Co(CO)_4]^-$

$[Co(CO)_4]^-$ · protonated piperidine

Co	-1.35183363943571	1.78715583659427	0.26228857337941
C	-1.27239341340133	3.46229623360696	-0.30911801039983
C	-0.44084586119499	1.56318048592777	1.76348907001132
C	-3.00863080991593	1.17330205313666	0.39019828572165
C	-0.55139777160349	0.79980249950795	-0.99479345561919
O	-1.20884015178976	4.55261377256564	-0.69020925148876
O	0.13609616642854	1.43776846435095	2.75937239426461
O	-4.08739564375006	0.76257751877434	0.46884458330517
O	-0.03493339857287	0.16607932360175	-1.80289091313750
H	-2.43228605522695	3.09511260817226	1.98539769400080
N	-2.71615174237969	3.55823302315245	2.84872832378768
C	-2.57053539177412	5.04684731362655	2.69748471975810
C	-3.60314745888503	5.58852674563805	1.72292302566731
C	-5.02078329010691	5.18716709381245	2.13089082514238
C	-5.12568337582881	3.66679571678656	2.25200603742015
C	-4.10179472458277	3.11696156996179	3.23094231720173
H	-2.05398429380707	3.22739011343035	3.55080621774405
H	-2.69751184973746	5.47536755452686	3.69183685093569
H	-1.55164432592888	5.22723363443378	2.36010068687448
H	-3.49466365333951	6.67361692323821	1.68955986585391
H	-3.38557472973621	5.21403841578183	0.72079903411859
H	-5.27681571353806	5.65299153422484	3.08813460129938
H	-5.73737148486438	5.55222614783597	1.39415963781095
H	-4.97554511721511	3.20403411652720	1.27441623535271
H	-6.11543252956904	3.36666451797541	2.59933562026037
H	-4.08930308619521	2.02894705028176	3.24646353985253
H	-4.27833465404382	3.48594973252206	4.24142949087939

$[Co(H)(CO)_4]$ · piperidine

Co	-1.39809181825408	1.73284540189458	0.18084689850484
C	-0.54815320622225	3.29135423682876	0.50880195149303
C	-0.94897980204285	0.30802228829470	1.19024887649738
C	-3.09445807936996	1.83443377685656	-0.42325377372919
C	-0.56456146306492	1.26307844231220	-1.37063818504884
O	-0.05076184184649	4.28232044960892	0.76767946688960
O	-0.69868704649345	-0.56501974587512	1.87793124666158
O	-4.18489504701666	1.91966798190980	-0.74202483740651
O	-0.04594815809253	0.97170801564440	-2.33792156294161
H	-2.06177323034924	2.12090660256039	1.44032819936612
N	-2.66290584552812	4.33030448260728	3.40949055172124
C	-2.89526355559586	5.53820430471680	2.61345747469198
C	-3.79267104366416	5.21284364759753	1.42316692791817
C	-5.10582891784796	4.57682773057584	1.88657342295512
C	-4.84413036688854	3.39112823397168	2.81885797160535
C	-3.91037372527876	3.79243337005228	3.95687890241139
H	-2.02054294894038	4.53917857820708	4.16436504880426
H	-3.37247733247285	6.33343937387824	3.21208255670062
H	-1.93070244976466	5.91783030319642	2.27201283349149
H	-3.98991285978717	6.12434568768860	0.85327276963802
H	-3.26437591366676	4.52395614159352	0.76003510449812
H	-5.69752749121634	5.32812814613008	2.42183541769146
H	-5.70017666699440	4.26051234332460	1.02605359880179
H	-4.38187325606215	2.57514079962953	2.25712660578261
H	-5.78387164072184	3.01328677544443	3.22939632202813
H	-3.67177826982054	2.92704900068625	4.57841144254766
H	-4.42601602299056	4.52895363065795	4.59758076842261

$[Co(H)(CO)_4]$

Co	-0.73004368328652	-0.29739924528329	0.32798859682650
C	-0.25760923766396	1.19040270302516	1.23991880726621
C	-2.22094064180736	-0.22681878656770	-0.68907237656546
C	0.50996278108178	-0.20748343541865	-1.00118374273809
C	-0.26435738955162	-1.89547820981263	1.02736561799401
O	-0.00352638386042	2.11530076950662	1.85312313259809
O	-3.19250162931982	-0.18858883651793	-1.28133051554082
O	-0.01686998583656	-2.89802141538218	1.50682810508298
O	1.28455391053855	-0.15301498041955	-1.82904636946304
H	-1.73824174029404	-0.37169656312983	1.40811474453957

$[Co(H)(CO)_3]$ · CO

Co	-1.05387526653317	-0.32119902841345	0.67008161223530

C	-0.50266046018781	1.18693775096326	1.51160593143471
C	-2.49221832169788	-0.24410948311524	-0.43023530910967
C	1.55584380811689	-0.13280807153969	-2.11313142747527
C	-0.50875749884537	-1.93159259838439	1.29886693571676
O	-0.23145908266283	2.11853436671905	2.11177554220690
O	-3.45718109637208	-0.20173036853775	-1.03745089032598
O	-0.24214125589322	-2.93763978537317	1.76622639921559
O	2.32681019746240	-0.07878379046041	-2.92996370023661
H	-2.02393502338690	-0.39040699185817	1.71493090633823

2.7.8. Solvent Coordination to Metal Center

$[Co(CO)_4]^-$ · MTBE

Co	-0.22984846376610	1.28382054669546	-0.05584882438056
C	-0.80081278378468	2.71069922401643	0.83017166412337
O	-1.17318250889120	3.64214925712459	1.40665450018729
C	1.52624495711675	1.13144275680160	0.14582749843264
C	-1.02952138934419	-0.15898490272595	0.58546646219662
C	-0.61363001016157	1.46806508953253	-1.77401001039528
O	-1.55962101260273	-1.10165605189057	1.00260793270186
O	2.67307430733773	1.03166189475083	0.27468510063917
O	-0.87411486180289	1.58929104232726	-2.89688781535207
O	-4.40342135876003	1.90571745898734	0.77151684949464
C	-4.67271981749004	0.77564020081566	1.58100608611471
C	-4.80768637800144	1.83922478924840	-0.61559479701863
C	-6.33378985074960	1.91390183049774	-0.71940919769736
H	-4.10006865519143	-0.10073340040428	1.26882670117985
H	-4.36523381954023	1.04532231831610	2.59113592310571
H	-5.73838746961843	0.51952596558681	1.59888290174836
H	-3.20126391621157	0.49131242037082	-1.13936184238473
H	-6.70215939564546	2.80968998092603	-0.21588419089573
H	-6.63695172913960	1.95533133391546	-1.76751050786054
H	-6.81025769335001	1.04059744457928	-0.27076130334642
C	-4.17155735638271	3.07850053082577	-1.23699951260406
C	-4.27570940897242	0.57729022406341	-1.29605865915728
H	-4.76116639411163	-0.32387185205353	-0.91828484794849
H	-4.46553064924249	0.63288638624425	-2.36926288030985
H	-4.51558132475670	3.97632456499122	-0.72021124553952
H	-4.43799187434435	3.15704201656099	-2.29231247270513
H	-3.08685122889141	3.02379089349571	-1.15130788842842

$[Co(MTBE)(CO)_4]^-$

Co	-1.20286571651416	1.59226618532788	0.21090962843956
C	-1.74119373326175	3.05633848983516	1.26696220451535
O	-1.51820543442114	4.05302887225228	1.82894347893958
C	0.17028760484769	1.21561127833450	1.23904513927743
C	-1.32808043443770	-0.12297416866691	-0.49931972256246
C	-0.68159272570121	2.47614858682303	-1.20825379456836
O	-0.90867788648714	-1.08148996805543	-1.01288113161332
O	1.17570395967194	1.03049872926401	1.79355056077982
O	-0.24746408460403	3.05557325942575	-2.11813118645346
O	-3.48637141415777	1.31833747488965	0.18714420714551
C	-3.92768374572755	0.71009528308798	1.39721984802319
C	-4.53542799467387	1.51906916166500	-0.80267909222357
C	-5.75228564121408	2.22151868149566	-0.19218383119418
H	-4.58699576239332	-0.14351524217670	1.21722583859541
H	-3.03453452646605	0.35936823213970	1.90617218777024
H	-4.44224474211100	1.42532002958360	2.04603629008430
H	-4.06048371992824	-0.31694028822701	-1.83849093890649
H	-5.44854015676000	3.15108322799536	0.29268918984899
H	-6.47038249771007	2.46195591093744	-0.97935936162566
H	-6.26069402729530	1.59578066878149	0.54185817372760
C	-3.93029897286858	2.41267264065914	-1.88069925426532
C	-4.93880145429659	0.17599543187384	-1.42146946298544
H	-5.39406303884425	-0.48994931599238	-0.68713556604732
H	-5.66590633730257	0.33301004062189	-2.22222875739176
H	-3.59718593389497	3.35832127775533	-1.45427873609289
H	-4.68146917608734	2.61437746130948	-2.64788337300569
H	-3.07228249365816	1.93248002265893	-2.34568691431076

References

(1) McKinlay, R. G.; Almeida, N. M. S.; Coe, J. P.; Paterson, M. J. Excited States of the Nickel Carbonyls Ni(CO) and Ni(CO)4: Challenging Molecules for Electronic Structure Theory. *J. Phys. Chem. A* **2015**, *119*, 10076–10083.

(2) Banerjee, A.; Coates, M. R.; Kowalewski, M.; Wikmark, H.; Jay, R. M.; Wernet, P.; Odelius, M. Photoinduced Bond Oscillations in Ironpentacarbonyl Give Delayed Synchronous Bursts of Carbonmonoxide Release. *Nat. Commun.* **2022**, *13*, 1337.

(3) Lischka, H.; Nachtigallová, D.; Aquino, A. J. A.; Szalay, P. G.; Plasser, F.; Machado, F. B. C.; Barbatti, M. Multireference Approaches for Excited States of Molecules. *Chem. Rev.* **2018**, *118*, 7293–7361.

(4) Feldt, M.; Phung, Q. M. Ab Initio Methods in First-Row Transition Metal Chemistry. *Eur. J. Inorg. Chem.* **2022**, *2022*.

(5) Becke, A. D. Density-Functional Exchange-Energy Approximation with Correct Asymptotic Behavior. *Phys. Rev. A (Coll. Park)* **1988**, *38*, 3098–3100.

(6) Lee, C.; Yang, W.; Parr, R. G. Development of the Colle-Salvetti Correlation-Energy Formula into a Functional of the Electron Density. *Phys. Rev. B* **1988**, *37*, 785–789.

(7) Chai, J.-D.; Head-Gordon, M. Long-Range Corrected Hybrid Density Functionals with Damped Atom–Atom Dispersion Corrections. *Phys. Chem. Chem. Phys.* **2008**, *10*, 6615.
